# *Trichoderma reesei* Isolated From Austrian Soil With High Potential for Biotechnological Application

**DOI:** 10.3389/fmicb.2021.552301

**Published:** 2021-01-28

**Authors:** Wolfgang Hinterdobler, Guofen Li, Katharina Spiegel, Samira Basyouni-Khamis, Markus Gorfer, Monika Schmoll

**Affiliations:** ^1^Center for Health and Bioresources, AIT Austrian Institute of Technology GmbH, Tulln, Austria; ^2^Department of Sustainable Agricultural Systems, Institute of Agricultural Engineering, University of Natural Resources and Life Sciences Vienna, Tulln, Austria

**Keywords:** *Trichoderma reesei*, *Hypocrea jecorina*, sexual development, pretreatment for biofuels and biogas, cellulases and xylanases, biocontrol, plant cell wall degradation, strain improvement

## Abstract

Fungi of the genus *Trichoderma* are of high importance for biotechnological applications, in biocontrol and for production of homologous and heterologous proteins. However, sexual crossing under laboratory conditions has so far only been achieved with the species *Trichoderma reesei*, which was so far only isolated from tropical regions. Our isolation efforts aimed at the collection of *Trichoderma* strains from Austrian soils surprisingly also yielded 12 strains of the species *T. reesei*, which was previously not known to occur in Europe. Their identity was confirmed with *tef1*- and *rpb2*-sequencing and phylogenetic analysis. They could clearly be distinguished from tropical strains including the common laboratory wildtypes by UP-PCR and genetic variations adjacent to the mating type locus. The strains readily mated with reference strains derived from CBS999.97. Secreted cellulase and xylanase levels of these isolates were up to six-fold higher than those of QM6a indicating a high potential for strain improvement. The strains showed different responses to injury in terms of induction of sporulation, but a correlation to alterations in the *nox1*-gene sequence was not detected. Several synonymous SNPs were found in the sequence of the regulator gene *noxR* of the soil isolates compared to QM6a. Only in one strain, non-synonymous SNPs were found which impact a PEST sequence of NoxR, suggesting altered protein stability. The availability of sexually fertile strains from middle Europe naturally producing decent amounts of plant cell wall degrading enzymes opens up novel perspectives for non-GMO strain improvement and biological pretreatment of plant biomass for bioethanol production. Moreover, the varied response of these strains to injury in terms of sporulation, which is independent of Nox1 and NoxR suggests that additional regulators impact this phenomenon in *T. reesei*.

## Introduction

The genus *Trichoderma* comprises fungi with widespread ecological functions, which are of high interest in research. Species of this genus include a considerable number of efficient biocontrol agents (Harman et al., 2004; Harman, 2011), the biotechnological workhorse *T. reesei* (Bischof et al., 2016) and species know to comprise pathogenic strains impacting mushroom farming (Komon-Zelazowska et al., 2007) or even threatening human health (Hatvani et al., 2013). Roughly 260 species are documented from this genus in current literature (Atanasova et al., 2013; Bissett et al., 2015), with a total of 443 species listed in Mycobank for *Trichoderma*^1^ (access on July 31st, 2020).

Among *Trichoderma* spp., *T. reesei* is among the best studied species (Schmoll et al., 2014, 2016), predominantly due to its broad application in biotechnology (Gudynaite-Savitch and White, 2016; Arnau et al., 2020). After the isolation of the wild-type strain QM6a from the Solomon Islands during World War II, its efficient production of cellulolytic enzymes was recognized (Bischof et al., 2016). Since then, decades of research, including mutation programs were aimed at improvement of cellulase production as well as optimization of *T. reesei* as a host for heterologous protein production (Seiboth et al., 2011; Paloheimo et al., 2016). The enhanced strains from these efforts are all derived from the original isolate QM6a.

The natural habitat of *Trichoderma reesei* is known to be a tropical rain forest with abundant decaying plant material (Druzhinina et al., 2011). The teleomorph of *T. reesei* was initially known as *Hypocrea jecorina*, a name which was frequently used in literature of this species as well, until the one fungus = one name nomenclature ended the century old practice of naming sexual and asexual forms independently (Bissett et al., 2015). Since then, only the name *T. reesei* is accepted. Phylogenetically the species *T. reesei* belongs to the *Longibrachiatum* clade, which comprises 26 putative species (Druzhinina et al., 2012) with the closest relatives being *Trichoderma parareesei*, *Trichoderma orientalis*, and *Trichoderma longibrachiatum*. Most analyzed isolates originate from tropical or subtropical regions like the Solomon Islands, French Guiana, Brazil, or Peru (Druzhinina et al., 2012). Genome analysis revealed that *T. reesei*, representing the *Longibrachiatum* clade is evolutionarily one of the youngest species of *Trichoderma* (Kubicek et al., 2011). The same study indicated that the rather poor performance of *T. reesei* in most biocontrol applications compared to other *Trichoderma* species may be due to the loss of multiple genes associated with antagonism and mycoparasitism.

As a highly efficient producer of homologous and heterologous proteins, manipulation of the genome of *T. reesei* is supported by optimized tools like non-homologous end joining deficient (NHEJ) strains and genome wide deletion primer design (Schuster et al., 2012), transient knock down of NHEJ (Chum et al., 2017), or CRISPR/Cas9 mediated genome editing (Liu et al., 2015). The genome of *T. reesei* is sequenced in high quality (Martinez et al., 2008; Marie-Nelly et al., 2014; Li et al., 2017) and manually annotated (Druzhinina et al., 2016; Schmoll et al., 2016; Li et al., 2017). Despite the broad array of versatile tools for genome manipulation, a common drawback for strain improvement lies in the fact that either the genes responsible for an improvement has to be known or that deleterious mutations are introduced by random mutagensis along with the desired mutations leading to an overall suboptimal result. The latter drawback can be avoided by the use of sexual crossing for strain improvement (Ashton and Dyer, 2016).

Breeding of industrial fungi for high efficiency production has been an aim for decades. Although the sexually propagating *H. jecorina* was identified as the teleomorph of *T. reesei* (Kuhls et al., 1996) and fruiting bodies were seen with an isolate from French Guiana (Lieckfeldt et al., 2000), fruitless attempts to achieve mating led to the assumption that *T. reesei* is a clonal species. Only about 11 years ago, after the discovery of peptide pheromone precursor genes in the genome, sexual development under laboratory conditions was achieved (Schmoll et al., 2004, 2010; Seidl et al., 2009; Hinterdobler et al., 2020). *T. reesei* is a heterothallic fungus with a common mating type structure of three mat-genes in the MAT1-1 locus and one mat-gene in the MAT1-2 locus (Seidl et al., 2009). Its pheromone system comprises two pheromone receptors HPR1 and HPR2 as well as two associate peptide pheromone precursors, HPP1 and PPG1 (Schmoll et al., 2010; Seibel et al., 2012). Additionally, also chemical communication is involved in mate recognition (Bazafkan et al., 2015). In contrast to many other fungal species, mating in *T. reesei* occurs on rich media like malt extract and is facilitated by light (Schmoll, 2013; Hinterdobler et al., 2020). Unfortunately, the parental strain of all strains currently used in industry and academia, QM6a, was found to be female sterile (Seidl et al., 2009). This defect is due to mutations in the *ham5* gene, causing erratic splicing and loss of function (Linke et al., 2015; Tisch et al., 2017). While this mutation can now be complemented, it still causes a considerable obstacle in industrial strain improvement by crossing.

Here, we identified and analyzed twelve *T. reesei* strains of both mating types unexpectedly isolated in the temperate climate zone in Austria (Central Europe), which readily mate with common *T. reesei* lab strains and each other. They show production of plant cell wall degrading enzymes and different inducibility of asexual development by injury. Their application for strain improvement by crossing renders them a valuable resource for diverse plant cell wall degrading enzymes using diverse substrates. Fungal strain improvement and/or GMO-free optimization of production is moreover crucial if the resulting strains are to be distributed in nature (biocontrol applications) or due to labeling requirements of products from GMO organisms by the EU^2^.

## Results

### *Trichoderma reesei* Occurs in Austrian Soil

**FIGURE 1:**
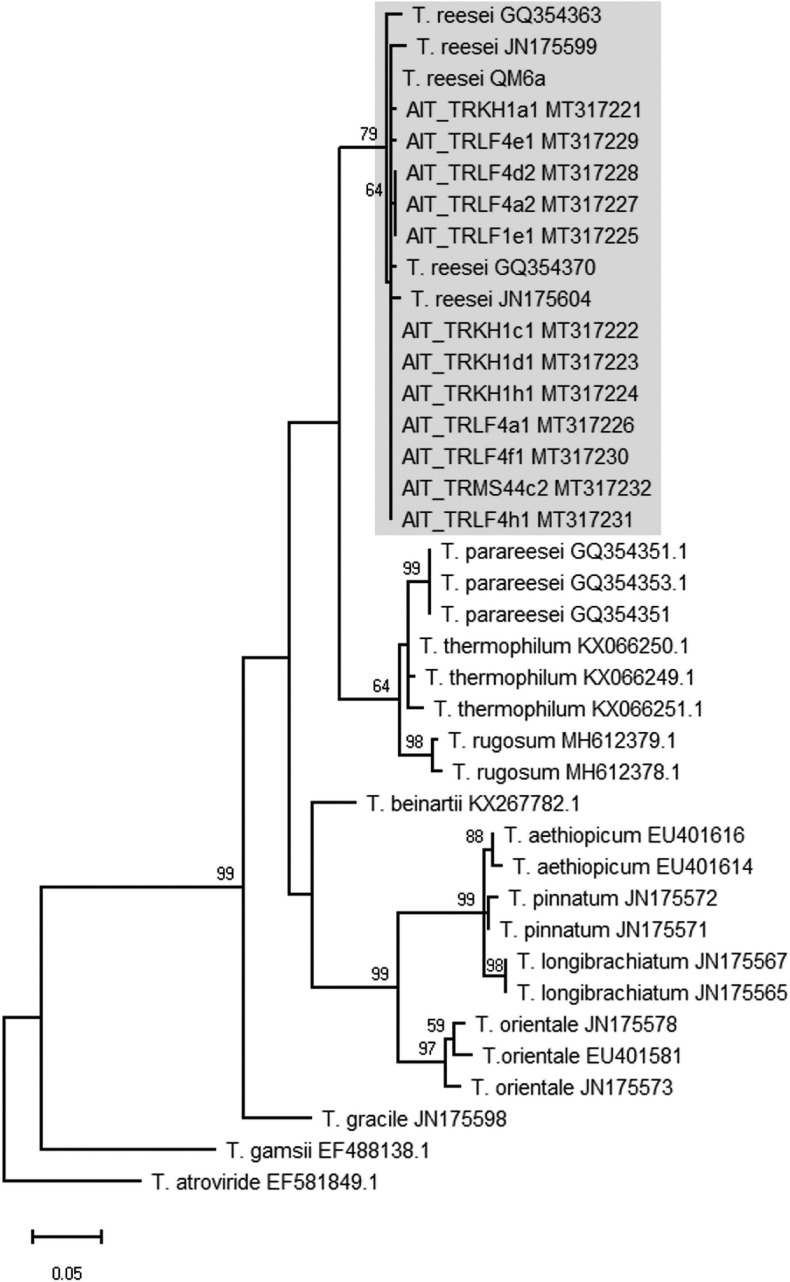
Phylogram obtained from an alignment of the *tef1* locus. The evolutionary history was inferred by using the Maximum Likelihood method and Tamura-Nei model (Tamura and Nei, 1993). The tree with the highest log likelihood (–1894.50) is shown. The bootstrap test was carried out with 1,000 replicates (Felsenstein, 1985) and values >50 are shown. Species names are given along with the GenBank accession number of the sequence (as retrieved from the NCBI nucleotide database) used for the analysis.

**FIGURE 2:**
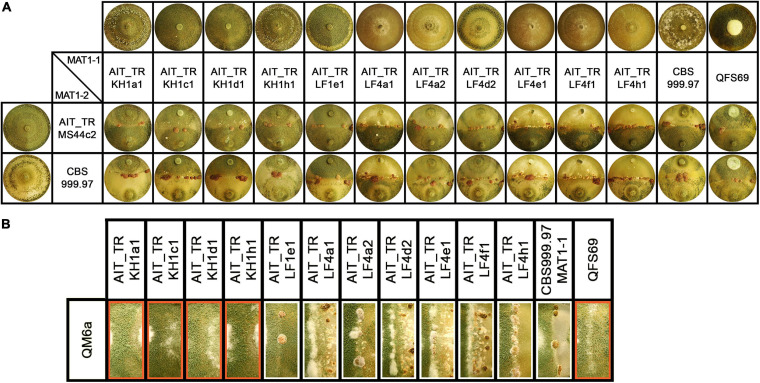
Growth and development of *T. reesei* strains isolated from Austrian soil. **(A)** Axenic growth and crossings of Austrian strains of MAT1-1 with AIT_TRMS44c2 (MAT1-2) and CBS999.97 MAT1-2 (wild-type control, female fertile). **(B)** Analysis of female fertility by crossing of Austrian soil isolates of MAT1-1 with the female sterile strain QM6a (MAT1-2). Abolished fruiting body formation indicates female sterility of the tested strains (Hinterdobler et al., 2020). Female sterile strains are indicated by red boxes. Strains were grown on malt extract agar plates (3% w/v) at 22°C with light-dark cycles. Phenotypes and fruiting body development are shown for day 14 after inoculation. One representative of three biological replicates is shown.

In the course of a collection project aimed at isolation of novel *Trichoderma* strains from agricultural soil, we surprisingly found *T. reesei* strains in four independent locations in Austria (for GPS data see Table 1). The phylogenetic species concept (PSC) determines a species as a monophyletic group based on evolutionary history (Lee et al., 2010). Hence, species identification for the isolates from Austria was performed by sequence analysis of the *tef1* diagnostic region (Samuels et al., 2002; Chaverri et al., 2003) and BLAST search against the NCBI nucleotide database. Phylogenetic analysis confirmed that the *tef1* sequences amplified from the 12 strains tentatively assigned to *T. reesei* indeed clustered with *T. reesei* QM6a and several other *T. reesei* strains. The closely related *T. parareesei* strains clustered separately (Figure 1). The diagnostic *rpb2* gene (Jaklitsch et al., 2008; Jaklitsch and Voglmayr, 2015) was used for additional confirmation for species identity, particularly because the pattern for AIT_MS44c2 in universal primers (UP-PCR) was divergent from the other strains (see also below). This analysis confirmed the species identity as *T. reesei* (Supplementary Figure 1 in Supplementary File 1). The 12 isolates showed clearly distinguishable phenotypes upon growth on plates with malt extract medium (Figure 2).

**TABLE 1:** Origin and identification of Austrian soil isolates.

				**GenBank Accession number**		
**Strain**	**Mating type**	**Isolation from**	**GPS Coordinates (isolation)**	***tef1***	***rpb2***	**INDEL/SNP region**
AIT_TRKH1a1	MAT1-1	Soil	47.03273, 15.37634	MT317221	MT809491	MW345895
AIT_TRKH1c1	MAT1-1	Soil	47.03273, 15.37634	MT317222	MT809492	MW345896
AIT_TRKH1d1	MAT1-1	Soil	47.03273, 15.37634	MT317223	MT809493	MW345897
AIT_TRKH1h1	MAT1-1	Soil	47.03273, 15.37634	MT317224	MT809494	MW345898
AIT_TRLF1e1	MAT1-1	Soil	47.45514, 16.25114	MT317225	MT809495	MW345899
AIT_TRLF4a1	MAT1-1	Soil	47.46148, 16.25278	MT317226	MT809496	MW345900
AIT_TRLF4a2	MAT1-1	Soil	47.45514, 16.25114	MT317227	MT809497	MW345901
AIT_TRLF4d2	MAT1-1	Soil	47.45514, 16.25114	MT317228	MT809498	MW345902
AIT_TRLF4e1	MAT1-1	Soil	47.45514, 16.25114	MT317229	MT809499	MW345903
AIT_TRLF4f1	MAT1-1	Soil	47.45514, 16.25114	MT317230	MT809500	MW345904
AIT_TRLF4h1	MAT1-1	Soil	47.45514, 16.25114	MT317231	MT809501	MW345905
AIT_TRMS44c2	MAT1-2	Soil	47.89873, 16.88850	MT317232	MT317233	MW345906

### Twelve Novel and Unique *T. reesei* Strains Are Distinguishable by Molecular Methods

**FIGURE 3:**
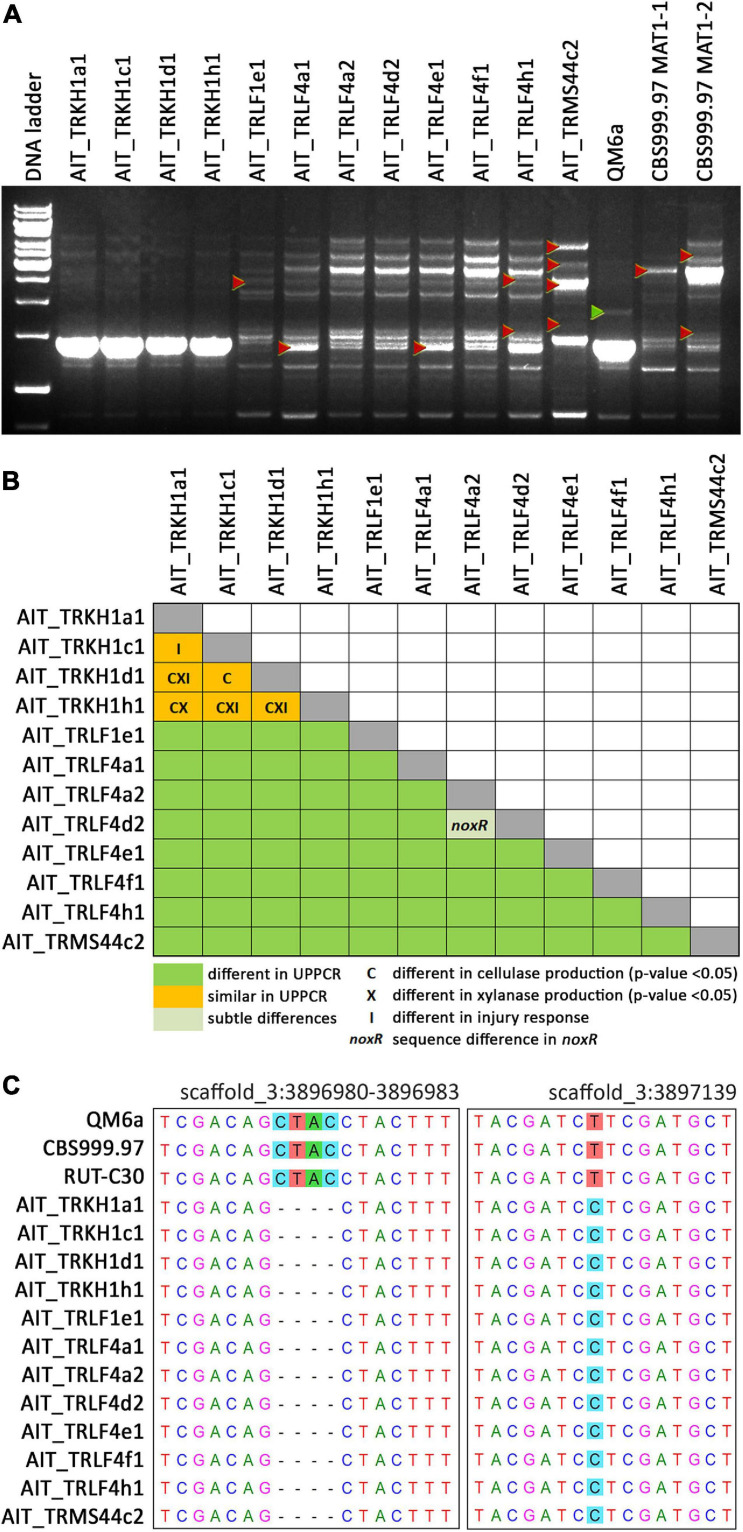
UP-PCR analysis, phenotypic differentiation and genetic alterations of strains. **(A)** UP-PCR of Austrian isolates along with QM6a and CBS999.97 MAT1-1 and MAT1-2 using as15inv as primer. For analysis with additional primers resulting in different patterns for separation of individual strains see also Supplementary Figures S2–S4 in Supplementary File 1. Triangles indicate bands indicative of differences to other strains. **(B)** Schematic representation of comparison of individual strains. UP-PCR band patterns and Supplementary Figures S2–S4 were used for evaluation. If no difference was found for band patterns, we considered sequence differences in noxR, altered enzyme production or injury response as well. **(C)** INDEL and SNP in the sequence flanking the mating type locus and specific for Austrian strains compared to laboratory strains in chromosome 3 (https://mycocosm.jgi.doe.gov/Trire_Chr/Trire_Chr.home.html; scaffold 3).

Previously, strains of the species *T. reesei* were not found in Europe and hence, *T. reesei* was assumed to be adapted to tropical climate. Consequently, we considered it of utmost importance to rule out any contamination by laboratory strains. In order to unequivocally distinguish these strains from our reference strains routinely used in the lab, we performed UP-PCR (Naeimi et al., 2011) using five different primers under different PCR conditions. All our isolates showed patterns distinct from the wild-type strains we are routinely using in the lab (Figure 3A, Supplementary Figure 2 in Supplementary File 1). The difference of QM6a from AIT_TRKH1a1, AIT_TRKH1c1, AIT_TRKH1d1, and AIT_TRKH1h1 is more subtle, but obvious in Figure 3A, showing one additional band migrating more slowly (green triangle) in QM6a. Moreover, these isolates also showed phenotypic differences with respect to cellulase regulation and injury response (see following sections). The differences between the individual isolates were not sufficiently clear in some cases and needed additional support (Figure 3A). Hence, we compared the banding patterns of all strains against each other and used additional criteria from our analyses for clarification (Figure 3B). Applying alternative primers, we could find characteristic patterns for most of the strains compared to the others (Supplementary Figures 3, 4 in Supplementary File 1). However, UP-PCR did not allow for clear separation of AIT_TRKH1a1, AIT_TRKH1c1, AIT_TRKH1d1, and AIT_TRKH1h1 from each other (Figure 3B). Therefore we compared the behavior of these strains in cellulase and xylanase production as well as injury response (see below). We found clear differences confirming that these strains are individuals rather than re-isolations of one and the same strain (Figure 3B). For further confirmation of the distinct origin of these strains, we screened the rapidly evolving region of the mating type locus for characteristics variations. We found a four base pair long INDEL and a SNP in the region adjacent to the mating type idiomorphs^3^, both of which are specific for the isolated strains and distinct from QM6a, RutC30, and CBS999.97 (Table 1 and Figure 3C).

### Austrian Isolates Are Sexually Fertile and Compatible Among Each Other

The biological species concept (BSC) supports the concept that sexually compatible individuals belong to the same species, while two populations that cannot mate represent different species (Lee et al., 2010). Sexual reproduction between strains of different species has not been shown for fungi previously and would contradict the BSC. Hence, the ability to mate with known and confirmed *T. reesei* strains can add further confirmation to species identity of the 12 strains investigated here.

Additionally, *T. reesei* strains with an origin in middle Europe and the potential of breeding for strain improvement would have a high potential for applications under natural conditions in this region. Consequently, we explored the prerequisites for that and found solid support for the strains belonging to the species *T. reesei*: Using mating type specific primers (Seidl et al., 2009) for *T. reesei*, we found that of the 12 new isolates, eleven strains comprised the MAT1-1 idiomorph and one strain was of MAT1-2. All strains showed efficient sporulation with a preference for asexual development in light, i.e., increased production of conidia in light compared to darkness. Due to the biotechnological importance of sexual development we tested all strains for mating (Schmoll, 2013) with fully fertile *T. reesei* strains under daylight conditions (Figure 2A). We found that all strains were able to undergo sexual development with CBS999.97 representing a fully fertile partner of opposite mating type under usual mating conditions.

Fruiting body formation of the new isolates with fully fertile strains started at 4–5 days after inoculation, which is roughly at the same time or even slightly earlier as seen with the control crossing of CBS999.97 MAT1-1 and CBS999.97 MAT1-2, our model strains for sexual development (Figure 2A). As female sterility was found to occur in natural strains of *T. reesei*, most importantly with QM6a (MAT1-2) (Seidl et al., 2009), we also tested whether our strains are female fertile by confrontation of the strains with the female sterile QM6a (MAT1-2). Indeed we found female sterility for four of our isolates (Figure 2B): AIT_TRKH1a1, AIT_TRKH1c1, AIT_TRKH1d1, and AIT_TRKH1h1. The only MAT1-2 strain, AIT_TRMS44c2, was crossed with a female sterile derivative of QM6a bearing the MAT1-1 locus, QFS69. Together with the fact that AIT_TRMS44c2 readily crossed with the obviously female sterile strains mentioned above, confirmed that AIT_TRMS44c2 is female fertile.

Interestingly, crossing of the MAT1-2 isolate AIT_TRMS44c2 with strains that could not form fruiting bodies with QM6a (Figure 2B, red boxes) was significantly delayed to 7–8 days for AIT_TRKH1c1, AIT_TRKH1d1 and AIT_TRKH1h1 as well as the control strain QFS69 or even 9 days with AIT_TRKH1a1. Also AIT_TRLF1e1 showed a delay to 6 to more than 7 days in all crossings. Consequently, the ability of all our new isolates to mate with different, known and confirmed *T. reesei* strains further confirmed species identity.

### Production of Cellulases and Xylanases

**FIGURE 4:**
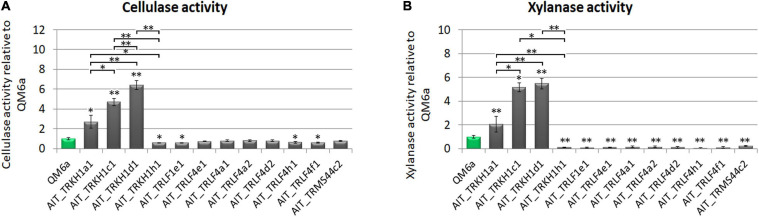
Enzyme production. **(A)** Cellulase activity and **(B)** xylanase activity represented by measurement of reducing sugars as released by enzymes secreted into the culture supernatant relative to wildtype strain QM6a. Strains were grown on Mandels Andreotti minimal medium with 1% (w/v) cellulose as carbon source in constant darkness for 72 h in 24-well plates with shaking. Error bars show standard deviations from three biological replicates. Statistically significant differences to wildtype are indicated by asterisks. Comparisons between individual strains are shown with brackets. *p-value < 0.05, **p-value < 0.01.

For *T. reesei* as an important species in biotechnological industry, production of enzymes is of particular interest. Therefore we tested our strain for production of cellulases and xylanases upon growth on Mandels Andreotti minimal medium with cellulose as carbon source. While the majority of the strains produced cellulases in the range of QM6a, some of the strains were considerably more efficient with up to a six-fold increase in cellulase efficiency (Figure 4A). Also for xylanase expression we found a considerably higher efficiency in some strains compared the QM6a (Figure 4B). As these strains showed also high cellulase production, they are the most interesting ones for further studies.

### A Strain Specific Response to Mycelial Injury

Asexual development is regulated in response to environmental conditions in fungi and is of high importance for application in biotechnology. Usually, sporulation in *T. reesei* is enhanced in light and diverse carbon sources modulate its efficiency (Friedl et al., 2008; Steyaert et al., 2010a,b). However, in some *Trichoderma* species, also injury was shown to induce sporulation (Casas-Flores et al., 2004). In the reference strain QM6a, we could not previously observe this phenomenon. Therefore we wanted to evaluate if this is a species specific characteristic or merely dependent on the strain.

**FIGURE 5:**
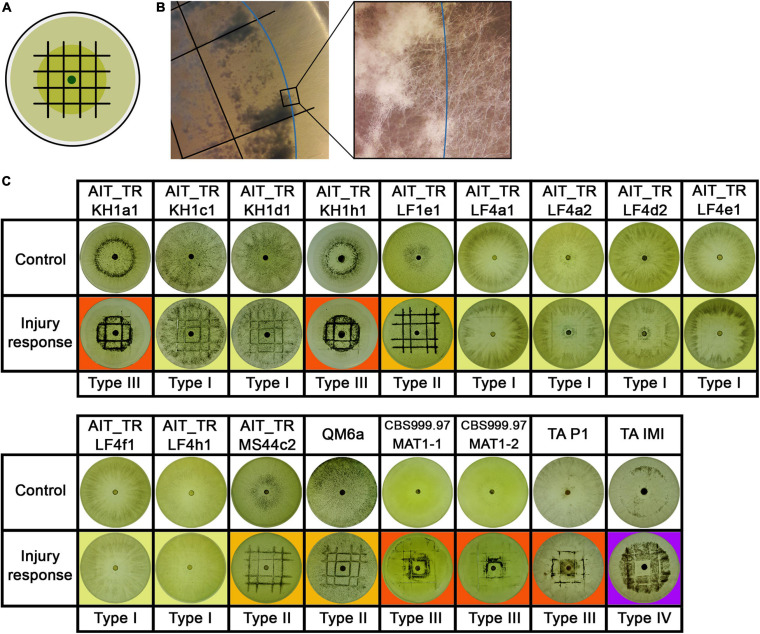
Injury response. **(A)** Illustration of the experimental setup. Strains were inoculated with an agar plug in the center of the petri dish. After 2 days of growth at 28°C in darkness, the mycelium (dark green) was injured under red safety light (black lines). Phenotypic changes of the young and developing (light green) mycelium were monitored 24 h after injury. **(B)** Shows the control strain *T. atroviride* IMI206040 (TA IMI). After injury (black lines) the young mycelium (blue line marks growth margin at timepoint of injury) grew dense, formed aerial mycelium and sporulated. The developing mycelium (right of blue line) shows undifferentiated morphology without sporulation after injury. **(C)** Shows response to injury of the tested strains. Four types of injury responses were observed: *Type 1* (yellow background) strains show slight to no response to injury. Strains of *type 2* (orange background) show clear sporulation close to the injury site. In *type 3* (red background), the young mycelium shows a widespread response to the injury. *Type 4* (purple background), represented only by TA IMI, shows a pattern similar to *type III* but with no sporulation around the inoculum.

**FIGURE 6:**
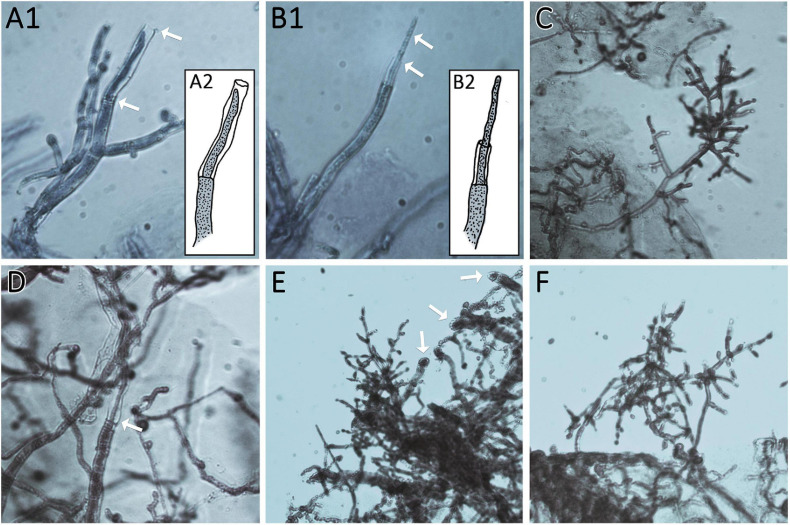
Thin regeneration hyphae and conidiophores emerge at the agar margin as response to previous injury. Edges of cut agar plugs are shown. (**A1,2** and **B1,2**) Show regenerating hyphae of AIT_LF4a1 growing from the last intact septum of the cut hyphae. Arrows mark thin regenerating hyphae and empty cell walls of cut hyphae. **(A2,B2)** Show illustrations of **(A1,B1)** for better visualization. **(C,D)** Show AIT_LF4a2. **(C)** Shows an emerging conidiophore from the agar margin. **(D)** Shows a regenerating hypha growing in the empty cell wall of a cut hypha (arrow). **(E,F)** show AIT_LF4e1 with emerging conidiophores. Arrows in **(E)** point at the cutting sites of hyphae at the agar margin. All images were taken at 400× magnification. Samples were stained with methylene blue solution.

**FIGURE 7:**
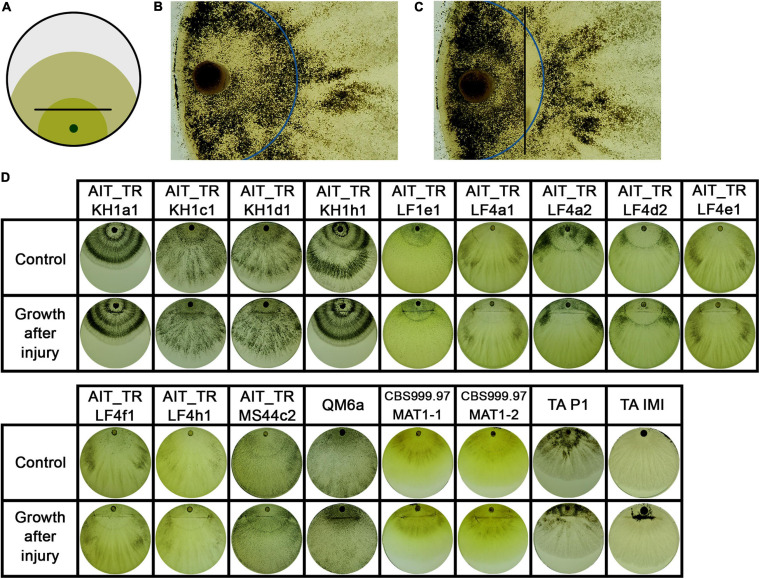
Growth after injury. **(A)** Illustration of the experimental setup. Strains were inoculated with an agar plug close to the border of the petri dish. After 2 days of growth at 28°C in darkness, the mycelium (dark green) was injured by cutting under red safety light (black line). Phenotypic changes were monitored 24 h after injury (light green). **(B, C)** The control strain *T. atroviride* P1 showed different sporulation pattern of the older compared to the younger colony area separated by the injury. Blue line indicates the growth margin at the timepoint of injury. In **(C)** the area between injury (black line) and the colony margin shows less sporulation compared to the old mycelium. **(D)** Shows phenotypic effects of early injury on the developing colonies compared to non-injured controls.

The strains were grown in constant darkness for two days and then injured by cutting (Figure 5A). *T. atroviride* IMI206040 and P1, for which a strong response to injury in terms of conidiation is well known was used as a control (Casas-Flores et al., 2004; Hernandez-Oñate et al., 2012). In many cases, the growth front at the time of injury became clearly distinguishable, because growth after the injury commenced with mycelium, in which no premature sporulation was induced (Figures 5B,C). We observed a strong response in some, but not all strains (Figures 5–7). In QM6a and CBS999.97 we found weak, but detectable induction of sporulation by injury (Figure 5).

Our results showed that the reaction to injury is highly strain-specific in *T. reesei*, and we could assign four distinguishable types of responses. We found a range from no reaction up to strong sporulation in the young mycelium present at the timepoint of injury (Figure 5). The reaction of *T. atroviride* IMI206040 with no sporulation close to the inoculum indicates a fast, coordinated signal transduction within the mycelium before the colony continues its radial growth (Figure 5C). While the morphological changes associated with sporulation are close to the injured mycelial areas in some strains, in other strains, injury triggers sporulation in the adjacent mycelium. Comparison between *type III* and *type IV* injury response (Figure 5C) suggests intracellular signal transduction in both directions for *type III* and only in direction toward the growth margin in *type IV*. Injury response of *type IV* shown here only for *T. atroviride* IMI206040 seems in addition to be medium dependent as a previous study (Hernandez-Oñate et al., 2012) described rather *type II* sporulation on PDA and minimal medium. Further research regarding the influence of the provided nutrients on intercellular injury signaling is need for a better understanding of the observed phenomenon.

Since growth and sporulation after injury were altered, we analyzed the site of injury shortly after cutting and the expected, thinner hyphae emerged from injured mycelial cells (Figure 6). Additionally, we were interested whether this growth after injury would be permanently and consistently altered. Therefore, we injured the young mycelium with one cut to be able to monitor further growth for a longer time (Figure 7A).

Interestingly, the separation of a small part of the young mycelium by injury as shown in Figure 7 did not affect the macroscopic phenotype of the emerging mycelium in all tested strains. In two strains, AIT_TRLF4d2 and *T. atroviride* P1 (Figures 7B,C), the separated, younger mycelium close to the growth margin at the timepoint of injury shows less sporulation than the emerging mycelium and the older mycelium close to the inoculum. This indicates a different influence of an injury event, either on young hyphae currently exploring new food sources compared to older hyphae, or an influence of the general size and energy reserves of the colony needed to react properly.

### Altered Response to Injury Is Not Due to Variations in Nox1, NoxR, or MAPkinase Genes

Since we saw differences in the injury response between many strains, also from the same locations, were interested if this might be reflected in the genome. Injury induced conidiation was found to be associated with an oxidative response and triggers NADPH oxidase (Nox)-dependent ROS production (Hernandez-Oñate et al., 2012). Fungi have between one and three NADPH oxidases (Aguirre et al., 2005), which are regulated by NoxR. Presence of the biosynthetic gene responsible for this process, *noxR* is required for fruiting body formation in *Aspergillus nidulans* (Lara-Ortiz et al., 2003). A function of *nox1* in female fertility and asexual development was reported for *Neurospora crassa* (Cano-Dominguez et al., 2008). Therefore we evaluated whether the varied response in our strains would be due to different gene variants of the NADPH oxidase encoding *nox1* or the regulator gene *noxR* in the strains of this study versus QM6a and CBS999.97.

For *nox1*, sequence analysis showed no alterations between QM6a, CBS999.97 and the 12 new isolates. However, the nucleotide sequences of *noxR* from the new isolates showed only a 98% identity to QM6a, but practically complete similarity with CBS999.97 (Supplementary File 1). Testing for SNPs in this sequence, which would alter the protein sequence, we found that despite numerous SNPs at the nucleotide level, the protein sequence remained the same for most strains. Or in other words, all of the SNPs were synonymous.

Interestingly, for one strain, AIT_TRLF4a2, we found alterations in the encoded protein sequence of NoxR in close proximity. In this region, no characteristic domains for NoxR are located. Also no phosphorylation sites were altered. However, testing for PEST regions, which are crucial for protein stability (Rechsteiner and Rogers, 1996) we found a potential PEST motif in QM6a and all other strains (SNAFPPTPPPEND, position 230-244, hydrophobicity index 29.94, and PEST score 8.84). In AIT_TRLF4a2, this sequence (nts marked in green in Supplementary File 1) is altered to SNVFPPTPPAEND, which causes a decrease in the PEST score to only 2.79 rendering it now a poor PEST motif. Due to the relevance of such PEST motifs for protein stability (Rechsteiner and Rogers, 1996), NoxR in AIT_TRLF4a2 may be more stable than in other strains. Since the phenotypic differences after injury are not limited to this strain, but are similar in other strains as well, the altered PEST sequence is unlikely to significantly contribute to characteristic injury response in *T. reesei*. Moreover, we also could not find indications that the minor sequence alterations would have an influence on sexual development in these strains.

Previously, the MAPkinase genes *tmk1* and *tmk3* were shown to be required for enhanced sporulation upon injury in *T. atroviride* (Medina-Castellanos et al., 2014). Therefore we analyzed the gene sequences of *tmk1* and *tmk3* for selected strains with different injury types (AIT_TRKHa1, AIT_TRKH1c1, AIT_TRLFe1, and AIT_TRMS44c2) in comparison with QM6a, RUTC30, and CBS999.97 (see Supplementary Material). Although these sequences formed groups according to their SNP patterns, they did not correlate with injury types in these strains.

We conclude that the differences in injury response that we observed for our isolates is not due to specifically altered alleles of *nox1, noxR, tmk1*, or *tmk3*.

## Discussion

*Trichoderma* spp. are among the few ascomycetes for which extensive reports on distribution in many areas all over the world are available (Druzhinina et al., 2006; Atanasova et al., 2013). These species are most commonly isolated, which has been attributed to their often mycoparasitic lifestyle and efficient enzyme production, which increase competitiveness in nature (Schuster and Schmoll, 2010; Druzhinina et al., 2011; Atanasova et al., 2013). [Early isolates originate from tropic areas, but detailed research in Europe revealed that the diversity of *Trichoderma/Hypocrea* in Europe consists of at least 75 holomorphic species (Jaklitsch, 2009, 2011) as well as several anamorphic species (Friedl and Druzhinina, 2012) and references therein]. Consequently, *Trichoderma* spp. are a major element of the mycoflora also in temperate regions. Due to their broad distribution, but also because of their efficiency in mycoparasitism and biocontrol of plant pathogens, *Trichoderma* spp. are included in commercial products for plant protection, which are applied in Europe as well. So far, no studies are available with investigations, whether strains applied with commercial biocontrol agents were possibly re-isolated during screening studies.

*Trichoderma reesei* only showed poor performance in mycoparasitism and biocontrol (Seidl et al., 2006) and is hence not commercially applied or thereby artificially distributed in nature. Consequently, isolation of *T. reesei* of non-natural origin would be due to an accidental contamination from labs working with this species and subsequent broad distribution, which is extremely unlikely. Actually, strains of *T. reesei* were not previously reported from screening studies in Austria (Wuczkowski et al., 2003; Friedl and Druzhinina, 2012) or in Europe (Jaklitsch, 2009, 2011). Unequivocal determination of species is of high and increasing importance due to the rising numbers of novel and interesting fungal isolates for diverse sources. Phylogenetic analysis using ITS sequences alone is not reliable and reproductive isolation is often difficult to confirm if sexual development cannot be achieved under laboratory conditions (Lücking et al., 2020). The strains we now describe here were confirmed to be *T. reesei* first by molecular methods using two diagnostic markers recommended for species identification, *tef1* and *rpb2* (Lücking et al., 2020) and phylogenetic analysis and second by crossing with known and accepted *T. reesei* strains. Although unisexual reproduction is known from some species and well-studied in *Cryptococcus neoformans* (Ni et al., 2011; Sun et al., 2014) there are no hints that this might occur in *T. reesei* or any other *Trichoderma* species. Complex phylogenies and reproductive strategies that render the reproductive mode unreliable for species determination are only known from lichen-associated fungi (Lücking et al., 2020), a case which is not comparable with *Trichoderma* spp. Hence, even if the molecular identification would leave room for interpretation, cross-species sexual development under laboratory conditions yielding fruiting bodies typical for *T. reesei* (Hinterdobler et al., 2020) would be an absolute novelty and can practically be excluded. Consequently, these 12 strains belong to the species *T. reesei*.

The fact that the 12 strains we investigated, originate from four individual samples raised the question whether they might be just re-isolations of one and the same strain from the respective location. Moreover, since *T. reesei* was never found in Europe before, it was important to unequivocally ensure that our screening study did not contain unwanted contaminants from *T. reesei* strains we routinely use in the lab. Also the potential introduction of *T. reesei* by the application of biocontrol products, which may be relevant for other taxonomic studies as outlined above, can rather be excluded for *T. reesei*, since commercial products are only known for the highly efficient *Trichoderma* species like *T. asperellum*, *T. harzianum* etc. UP-PCR and specific mutations clearly showed that no contaminations with lab strains had occurred and that these isolates indeed originated from Austrian agricultural soil. UP-PCR also showed that the isolates from different sites showed clearly different patterns. However, strains from the same site showed very similar patterns. In some cases, we therefore included also enzyme expression, injury response and phenotype in our analyses, which eventually allowed for discrimination between strains (Figure 2B). Therefore, we assume that the multiple strains from the separate sites are indeed individuals, albeit closely related, which suggests that sexual development and recombination likely have occurred in the past. This is particularly interesting with the female sterile strains, which are also clearly distinguishable. As they all are of the same mating type, strains of the opposite mating type can also be expected to occur in this habitat and should be female fertile.

Already in the initial study reporting sexual development with *T. reesei* under laboratory conditions (Seidl et al., 2009), the issue of female sterile strains in this species was broached. Early studies with *T. reesei* on the topic came to the conclusion, that it would be an asexually propagating, clonal species (Kuhls et al., 1996). However, the existence and evolutionary survival of exclusively clonal species was questioned (Taylor et al., 1999) and considered a rather transient phenomenon aimed to avoid the efforts to reproduce sexually. Thereby, both sexual and asexual propagation bear costs and benefits for fungi (Nieuwenhuis and James, 2016). The strains we isolated here represent interesting examples for such a strategy. We isolated both female fertile and female sterile strains, suggesting that strategies to avoid the costs of sex are occurring naturally and are unlikely to be associated with climate or a specific habitat. However, previously also loss of female fertility after repeated asexual propagation was reported and this loss was assumed to be genetic rather than epigenetic (Saleh et al., 2012). The emerging female sterile strains then propagated more efficiently via asexual reproduction without investing energy in mating and fruiting body formation and invaded the whole population (Saleh et al., 2012). Consequently, the isolation of female sterile strains could also be due to an environment in which the conditions for sexual development were not met and female fertility was lost. Thereafter the resulting female sterile strains could have been more successful in colonization of the respective habitat.

The achievement of sexual development under laboratory conditions for *T. reesei* (Seidl et al., 2009) represented already an important innovation in the field of strain improvement. However, initially the application was mainly seen with proprietary industrial strains and in academia for fungal genetics and strain improvement. With the strains we introduce here, a novel opportunity opens up. We can now use natural strains from Europe, which we can improve for their degradative performance without genetic manipulation, which is still seen critically. Particularly with (decentralized) pretreatment for biogas and biofuel production from agricultural cellulosic waste these strains will be a valuable resource. Deposition of waste does then not cause environmental issues with GMO distribution and pretreatment could even be done on site on the field. Moreover, in-process production of enzymes can be supported by natural strain improvement while alleviating environmental issues of waste disposal.

Last but not least, for the broad applications of enzymes in customer products the market pull for non-GMO ingredients is rising, and EU regulations for labeling of GMO use in production processes^4^ increase customer awareness. Hence, production without the use of GMO organisms can be an interesting future perspective for the use of natural enzyme producer strains like those introduced in this study. We could already achieve a proof of concept for natural strain improvement by crossing of these strains for enhanced enzyme production (Basyouni-Khamis and Schmoll, unpublished), which supports the applicability of this approach. First steps in this direction were previously reported for *Myceliophthora heterothallica* (Aguilar-Pontes et al., 2016). Besides practical application, we can thereby also follow up which natural mechanisms become enhanced when enzyme production is increased due to natural recombination.

While the use of these strains for improvement of enzyme production is most straight forward with *T. reesei*, also the potential for biocontrol applications should be considered. *Trichoderma* spp. have a long history in safe application in agriculture worldwide and are known as beneficial plant symbionts (Guzman-Guzman et al., 2019). Also *T. reesei* was previously shown to be effective in plant protection (Seidl et al., 2006), albeit with lower performance than known biocontrol agents, which is why it was not used commercially for this purpose so far. Moreover, previously only tropical strains of *T. reesei* were known, which were not assumed to survive in the European climate. Obviously, this assumption has to be revised, since we could isolate *T. reesei* independently from different locations in Austria. Therefore, the possibility to adapt *T. reesei* to antagonism against plant pathogens by sexual development warrants exploration, particularly with respect to oomycetes for which the cellulosic cell wall should be subject to efficient degradation by the potent enzyme machinery of *T. reesei*. First studies with *T. reesei* reference strains against *Pythium ultimum* (Seidl et al., 2006) and *Phytophthora infestans* (Schmoll, unpublished), showed already promising results.

## Materials and Methods

### Strains and Cultivation Conditions

*Trichoderma reesei* (syn. *H. jecorina*) wildtype strains QM6a [female sterile (Linke et al., 2015; Tisch et al., 2017)], MAT1-2, ATCC 13631; (Martinez et al., 2008), CBS999.97 [female fertile, both mating types; (Lieckfeldt et al., 2000; Seidl et al., 2009)] and QFS69 [female sterile, MAT1-1; (Bazafkan et al., 2015)] were applied as controls and mating partners. Strains were maintained on malt extract agar.

For determination of enzyme production, strains were grown in 24-well plates with 2 mL of Mandels Andreotti minimal medium (Mandels and Andreotti, 1978) with 1% (w/v) microcrystalline cellulose as carbon source for 72 h at 28°C in darkness. Enzyme activity was determined as described previously (Mellitzer et al., 2012) from three biological and tree technical replicates with QM6a as control. Statistical significance was calculated using Student’s *t*-Test in RStudio (compare_means, ggpubr v0.3.0). Sexual crossing was performed as described previously (Seidl et al., 2009; Schmoll, 2013). Strains were inoculated in similar distances to enable analysis of delays in fruiting body formation. Differences in growth rates between strains, which could alter the time to fruiting body formation, were negligible. Crossings were performed in three biological replicates and fruiting body formation was monitored daily.

All chemicals mentioned were supplied by Roth Lactan (Karlsruhe, Germany) unless stated otherwise.

### Isolation of Strains From Soil

Soil samples in which *T. reesei* strains were detected originated from four different locations in Austria (Table 1). One gram of soil was suspended in 10 mL of phosphate buffered saline (PBS) containing 0.1% (w/v) Triton X-100. After homogenization of 50 rpm for 30 min, the suspension was diluted 1:100 and 1:1000 with PBS and 100 μL were applied to malt extract agar (Merck, Darmstadt, Germany) containing 50 mg/L Rose Bengal sodium salt (Sigma, Steinheim, Germany). After incubation at room temperature under daylight conditions, appearing fungal colonies were isolated and subjected to single spore separation to obtain pure cultures. The strains are available for non-commercial academic use.

### Nucleic Acid Isolation and Analysis

DNA extraction from young mycelia grown on malt extract agar plates was performed as described previously (Liu et al., 2000). Mating type determination of isolates was performed by PCR as described previously (Seidl et al., 2009). A diagnostic fragment of the genomic region encoding translation elongation factor 1 alpha (*tef1*) was amplified using the primers EF1-728F (Carbone et al., 1999) and TEF1 rev (Samuels et al., 2002). Primers for amplification of *rpb1* and the INDEL/SNP containing region were designed based on the genome sequence publicly available at JGI (Martinez et al., 2008; Grigoriev et al., 2014; Li et al., 2017).

Amplification of sequences of *nox1* and *noxR* was performed using primers nox1F1, nox1R1, noxRF1, and noxRR1. For primer sequences see Table 2.

**TABLE 2:** Oligonucleotides used in this study.

**Purpose**	**Name**	**Sequence**	**References**
UP-PCR	L45	5′-GTAAAACGACGGCCAGT-3′	Bulat et al., 1998
UP-PCR	3-2	5′-TAAGGGCGGTGCCAGT-3′	Bulat et al., 1998
UP-PCR	AA2M2	5′-CTGCGACCCAGAGCGG-3′	Lübeck et al., 1998
UP-PCR	AS15inv	5′-CATTGCTGGCGAATCGG-3′	Bulat et al., 1998
UP-PCR	L15/AS19	5′-GAGGGTGGCGGCTAG-3′	Lübeck et al., 1998
*nox1* amplification	nox1F1	5′-ATCAAGAGGAGGGATTCC-3′	This study
*nox1* amplification	nox1R1	5′-TTGAGAGGCATAAAGTCAG-3′	This study
*noxR* amplicication	noxRF1	5′-AGCGAGAGATTAGGTTAAAG-3′	This study
*noxR* amplicication	noxRR1	5′-AGGGCAGTAACGTACCTC-3′	This study
*tef1* amplification	EF1-728F	5′-CAT CGA GAA GTT CGA GAA GG-3′	Carbone et al., 1999
*tef1* amplification	TEF1rev	5′-GCC ATC CTT GGA GAT ACC AGC-3′	Samuels et al., 2002
*rpb2* amplifiation	rpb2v3F	5′-CATTTCCCAGACAGAAGGTAG-3′	This study
*rpb2* amplification	rpb2v3R	5′-GGAATAGTTGGTGAGGAAGAAA-3′	This study
INDEL/SNP region	MATovF	5′-GTCTCCCCCACAAGTTCTCG-3′	This study
INDEL/SNP region	MATovR	5′-TGATCCACCTGCGTTACGAC-3′	This study

Sequences were aligned using MUSCLE and adjusted in MEGA-X [Version 10.0.5, (Kumar et al., 2018)]. Phylogenetic analysis was done using the Maximum Likelihood method and Tamura-Nei model (Tamura and Nei, 1993). Trees with highest log likelihood are shown in Figure 1 and Supplementary Figure 1 for *tef1* and *rpb1* marker sequences, respectively. The bootstrap test was carried out with 1,000 replicates (Felsenstein, 1985). Sequences were retrieved from the NCBI nucleotide database for *T. reesei*, *Trichoderma thermophilum* (Qin and Zhuang, 2016), *T. parareesei*, *Trichoderma rugosum*, *Trichoderma beinartii*, *Trichoderma aethiopicum*, *Trichoderma gracile*, *Trichoderma orientale*, *Trichoderma pinnatum*, *T. longibrachiatum*, *Trichoderma Gamsii*, and *T. atroviride*. The diagnostic sequences for *tef1* and *rpb2* of the strains investigated in this study are deposited at GenBank, for accession numbers see Table 1.

### Analysis of Injury Response

For the injury assays, round agar plugs of well grown cultures were used as inoculum. *T. atroviride* strains P1 (ATCC 74058) and IMI206040 (Kubicek et al., 2011) as well as *T. reesei* strains CBS999.97 (MAT1-2 and MAT1-1) and QM6a (Martinez et al., 2008) were used as control. For both assays, 90 mm petri dishes containing 3% (w/v) malt extract medium were used. Strains were grown in constant darkness at 28°C for two days, injured as shown in Figures 5A, 7A under red safety light. Consequences of injury were documented and analyzed 24 hours after injury. Three biological replicates were performed.

### UP-PCR Fingerprinting

For PCR using UP-PCR, DNA was isolated using the DNAeasy plant mini kit (QIAGEN, Heidelberg, Germany). UP-PCR was performed essentially as described earlier (Naeimi et al., 2011). Primers were tested individually and in combinations using gradient PCR with annealing temperatures ranging from 50–65°C. AS15inv, L45, 3-2 and a combination of L15/AS19 showed best results and were used for PCR amplification with DreamTaq Green Polymerase (ThermoFisher Scientific, Waltham, MA, United States).

## Data Availability Statement

All datasets relevant for this study are included in the article/Supplementary Material. Sequence information is available either online under the provided GenBank accession numbers or in supplementary material as noted in the text.

## Author Contributions

WH contributed to injury analysis, analysis of phenotype, and sexual development, phylogenetic analyses, statistical evaluations, and MAPkinase analysis as well as to figure design, interpretation, and writing of the manuscript. GL isolated and identified strains, and contributed experimental work for UP-PCR analysis, injury analysis, and amplification of *nox1* and *noxR* sequences. KS performed analysis of sexual development. SB-K performed analysis of enzyme production. MG performed analysis of *nox1* and *noxR* sequences. MS conceived of the study and designed experiments, contributed to analysis of *nox1*, *noxR*, and MAPkinase sequences, to figure design, interpreted results, and wrote the manuscript. All authors read and agreed to publication of the final manuscript.

## Conflict of Interest

The authors declare that the research was conducted in the absence of any commercial or financial relationships that could be construed as a potential conflict of interest.

## Footnotes

1 www.mycobank.org

2 https://ec.europa.eu/food/plant/gmo/legislation_en

3 https://mycocosm.jgi.doe.gov/Trire_Chr/Trire_Chr.home.html; scaffold 3:3896980-3896983 and scaffold 3:3897139.

4 https://ec.europa.eu/food/plant/gmo/legislation_en
