# Supplementary material for: Trichoderma reesei Isolated From Austrian Soil With High Potential for Biotechnological Application
Source: Front Microbiol. 2021 Jan 28;12:552301. doi: 10.3389/fmicb.2021.552301 (PMC7876326; doi:10.3389/fmicb.2021.552301)
Supplement: Supplementary file 1 [file Data_Sheet_1.pdf]

Trichoderma reesei isolated from Austrian soil with high potential for biotechnological application

Wolfgang Hinterdobler[1], Guofen Li[1], Katharina Spiegel[1],Samira Basyouni-Khamis[1,2], Markus Gorfer[1] and Monika Schmoll[1]*
[1]*AIT Austrian Institute of Technology GmbH, Center for Health and Bioresources, Konrad Lorenz Strasse 24, 3430 Tulln, Austria* [2]*University of Natural Resources and Life Sciences Vienna, Department of Sustainable Agricultural Systems, Institute of Agricultural Engineering, Konrad-Lorenz Strasse 24, 3430 Tulln, Austria*

Supplementary material

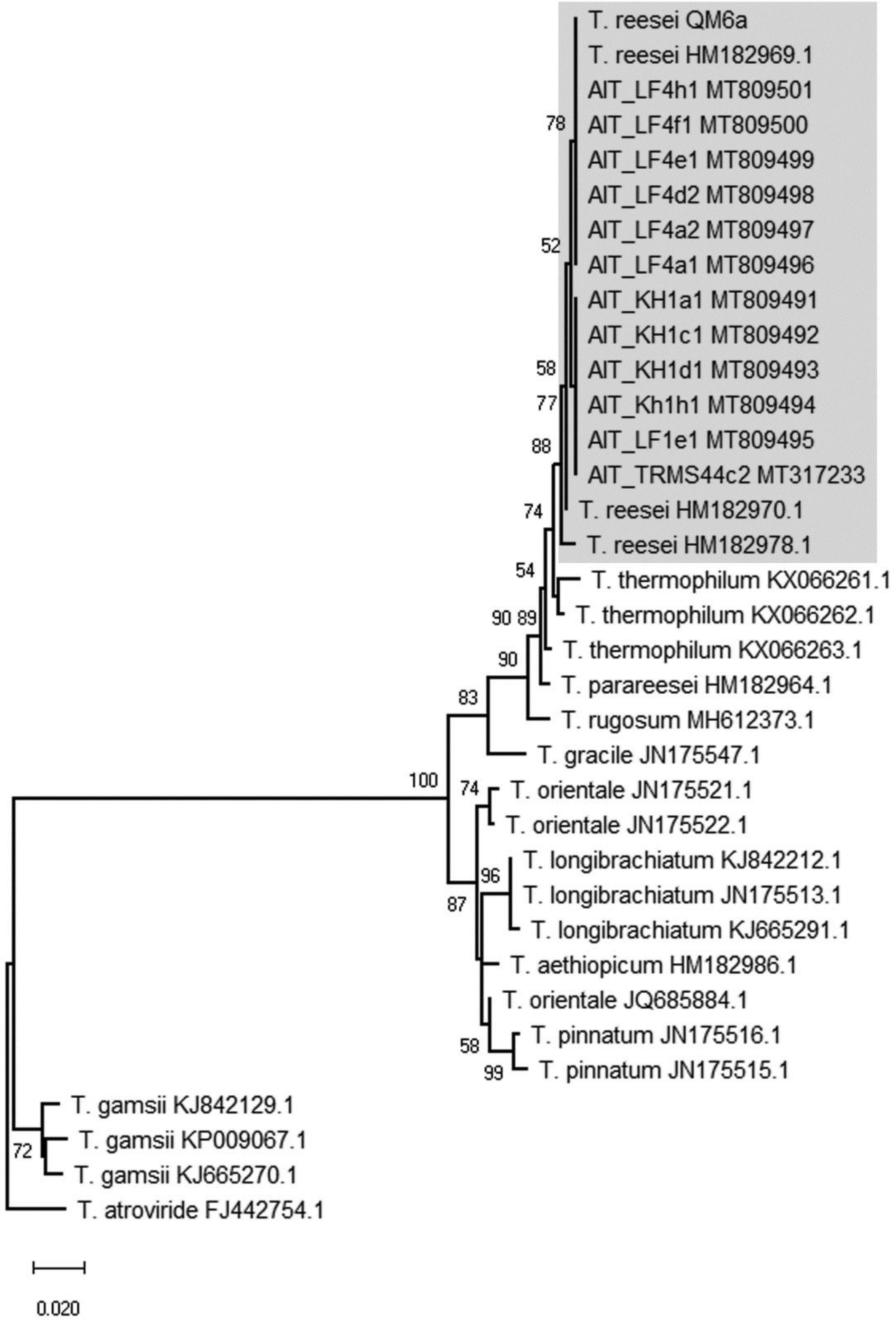

Figure S1. **Phylogram obtained from an alignment of *rpb2* loci**. The evolutionary history was inferred by using the Maximum Likelihood method. The tree with the highest log likelihood (-2069.78) is shown. The bootstrap test was carried out with 1000 replicates (Felsenstein J., 1985) and values >50 are shown.Species names are given along with the GenBank accession numbers of the sequence used for the analysis. Bootstrap values >50 are shown.

Figure S2

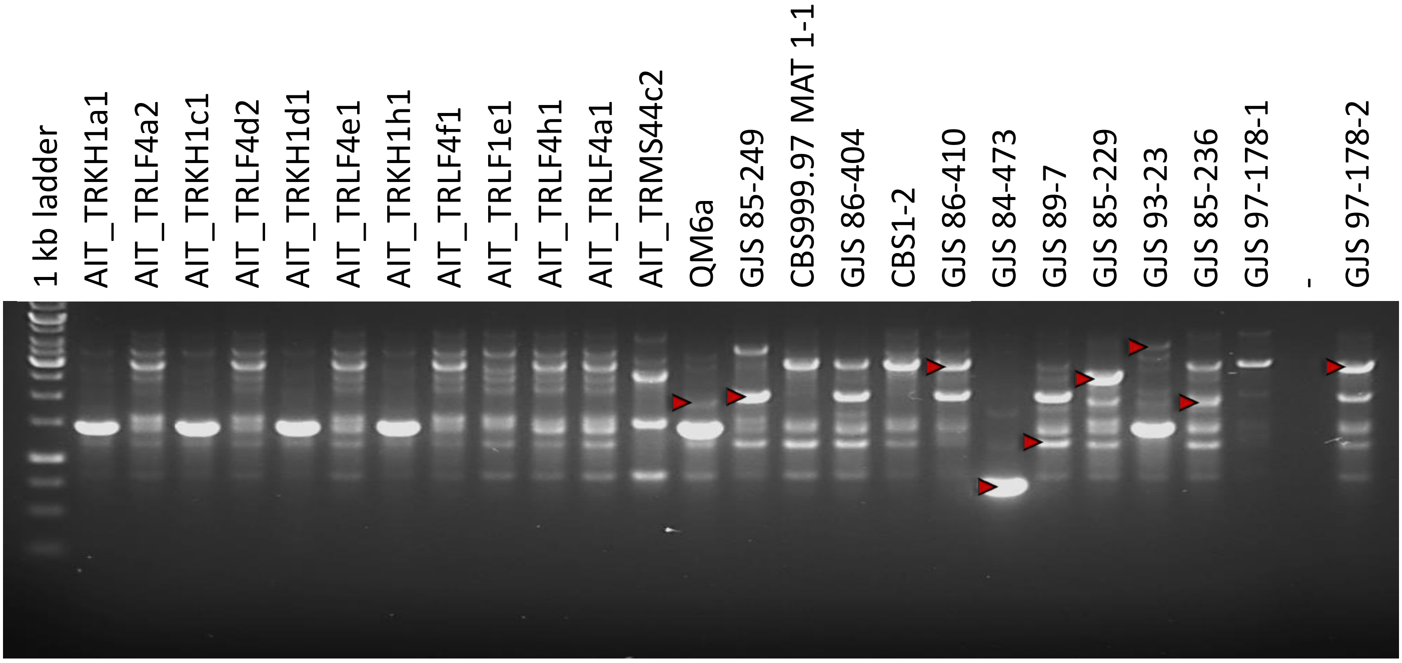

Figure S2. UP-PCR analysis and phenotypic differentiation of strains. (A) UP-PCR of Austrian isolates along with tropical isolates (Kuhls et al., 1996; Lieckfeldt et al., 2000; Seidl et al., 2009) using as15inv as primer. GJS, collection of G. J. Samuels, USDA Beltsville, USA. For analysis with additional primers resulting in different patterns for separation of individual strains see also Figure 3A, supplementary figures S3 and S4. Triangles indicates bands indicative of differences to other strains.

Figure S3

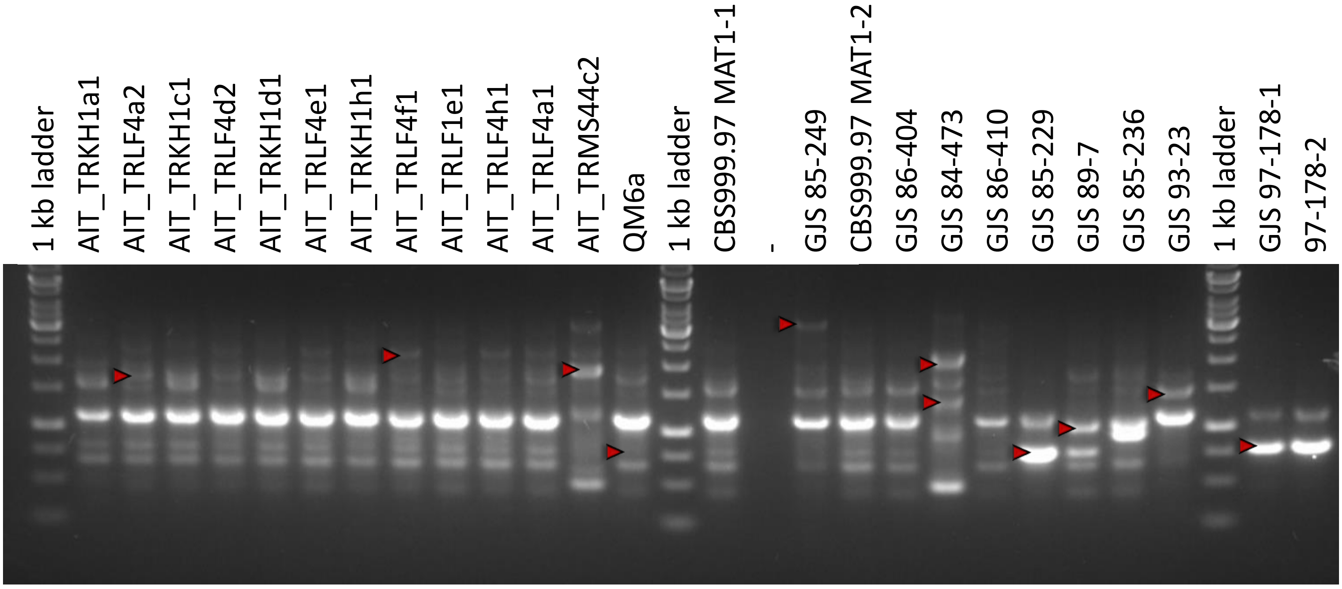

Figure S3. UP-PCR analysis and phenotypic differentiation of strains. (A) UP-PCR of Austrian isolates along with tropical isolates (Kuhls et al., 1996; Lieckfeldt et al., 2000; Seidl et al., 2009) with primers L15 andAS19. GJS, collection of G. J. Samuels, USDA Beltsville, USA. For analysis with additional primers resulting in different patterns for separation of individual strains see also Figure 3A, supplementary figures S2 and S4. Triangles indicates bands indicative of differences to other strains.

Figure S4

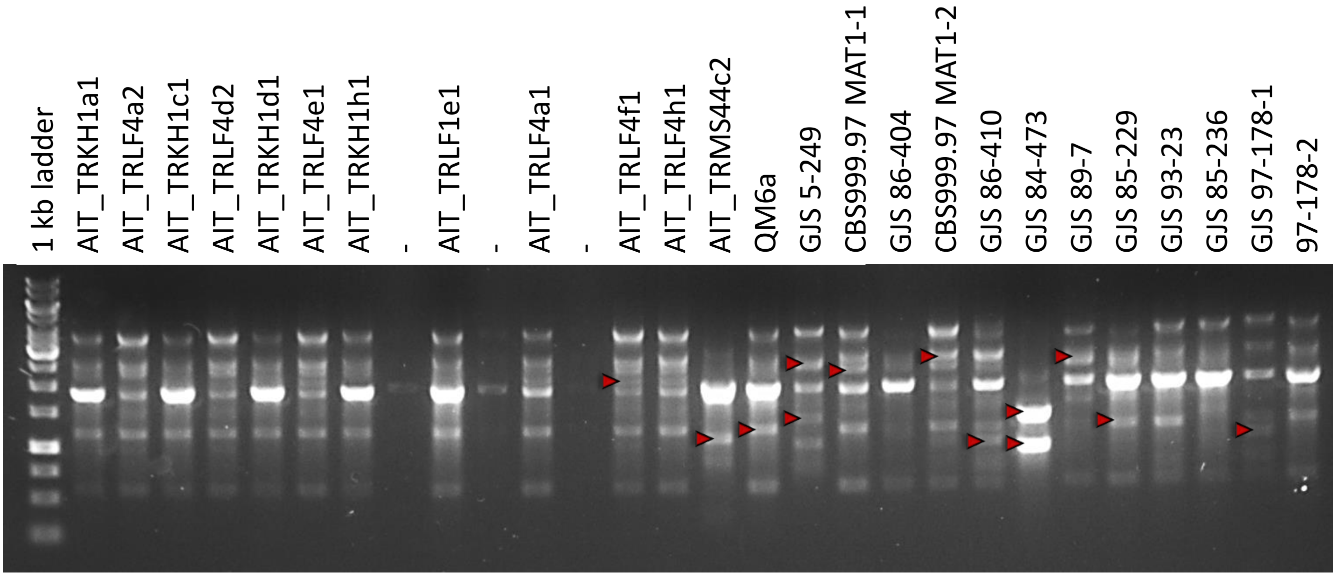

Figure S4. UP-PCR analysis and phenotypic differentiation of strains. (A) UP-PCR of Austrian isolates along with tropical isolates (Kuhls et al., 1996; Lieckfeldt et al., 2000; Seidl et al., 2009) using primer 3-2. GJS, collection of G. J. Samuels, USDA Beltsville, USA. For analysis with additional primers resulting in different patterns for separation of individual strains see also Figure 3A, supplementary figures S2 and S3. Triangles indicates bands indicative of differences to other strains.

REFERENCES

Kuhls, K., Lieckfeldt, E., Samuels, G.J., Kovacs, W., Meyer, W., Petrini, O., et al. (1996). Molecular evidence that the asexual industrial fungus *Trichoderma reesei* is a clonal derivative of the ascomycete *Hypocrea jecorina*. *Proc Natl Acad Sci U S A* 93(15)**,** 7755-7760.

Lieckfeldt, E., Kullnig, C.M., Samuels, G.J., and Kubicek, C.P. (2000). Sexually competent, sucrose- and nitrate-assimilating strains of *Hypocrea jecorina* (*Trichoderma reesei*) from South American soils. *Mycologia* 92(3)**,** 374 - 380.

Seidl, V., Seibel, C., Kubicek, C.P., and Schmoll, M. (2009). Sexual development in the industrial workhorse *Trichoderma reesei*. *Proc Natl Acad Sci U S A* 106(33)**,** 13909-13914.

Sequence analysis of *T. reesei* NoxR

```
                             *        20         *        40         *        60         *        80
CBS1-2_nox : TTCTTTACACTGCAGCACAGAGGCGTTACACGGACGGAGCGCCCCTGCGAAAAACATAATAAGTCGGGTGACATACAGATCAGCAT :   86
MS44c2_nox : TTCTTTACACTGCAGCACAGAGGCGTTACACGGACGGAGCGCCCCTGCGAAAAACATAATAAGTCGGGTGACATACAGATCAGCAT :   86
KH1a1_noxR : TTCTTTACACTGCAGCACAGAGGCGTTACACGGACGGAGCGCCCCTGCGAAAAACATAATAAGTCGGGTGACATACAGATCAGCAT :   86
CBS1-1_nox : TTCTTTACACTGCAGCACAGAGGCGTTACACGGACGGAGCGCCCCTGCGAAAAACATAATAAGTCGGGTGACATACAGATCAGCAT :   86
LF4f1_noxR : TTCTTTACACTGCAGCACAGAGGCGTTACACGGACGGAGCGCCCCTGCGAAAAACATAATAAGTCGGGTGACATACAGATCAGCAT :   86
LF4e1_noxR : TTCTTTACACTGCAGCACAGAGGCGTTACACGGACGGAGCGCCCCTGCGAAAAACATAATAAGTCGGGTGACATACAGATCAGCAT :   86
LF4d2_noxR : TTCTTTACACTGCAGCACAGAGGCGTTACACGGACGGAGCGCCCCTGCGAAAAACATAATAAGTCGGGTGACATACAGATCAGCAT :   86
LF4a1_noxR : TTCTTTACACTGCAGCACAGAGGCGTTACACGGACGGAGCGCCCCTGCGAAAAACATAATAAGTCGGGTGACATACAGATCAGCAT :   86
LF1e1_noxR : TTCTTTACACTGCAGCACAGAGGCGTTACACGGACGGAGCGCCCCTGCGAAAAACATAATAAGTCGGGTGACATACAGATCAGCAT :   86
KH1d1_noxR : TTCTTTACACTGCAGCACAGAGGCGTTACACGGACGGAGCGCCCCTGCGAAAAACATAATAAGTCGGGTGACATACAGATCAGCAT :   86
KH1c1_noxR : TTCTTTACACTGCAGCACAGAGGCGTTACACGGACGGAGCGCCCCTGCGAAAAACATAATAAGTCGGGTGACATACAGATCAGCAT :   86
KH1h1_noxR : TTCTTTACACTGCAGCACAGAGGCGTTACACGGACGGAGCGCCCCTGCGAAAAACATAATAAGTCGGGTGACATACAGATCAGCAT :   86
LF4a2_noxR : TTCTTTACACTGCAGCACAGAGGCGTTACACGGACGGAGCGCCCCTGCGAAAAACATAATAAGTCGGGTGACATACAGATCAGCAT :   86
LF4h1_noxR : TTCTTTACACTGCAGCACAGAGGCGTTACACGGACGGAGCGCCCCTGCGAAAAACATAATAAGTCGGGTGACATACAGATCAGCAT :   86
QM6a_cDNA  : TTCTTTACACTGCAGCACAGAGGCGTTACACGGACGGAGCGCCCCTGCGAAAAACATAATAAGTCGGGTGACATACAGATCAGCAT :   86
QM6a_noxR  : TTCTTTACACTGCAGCACAGAGGCGTTACACGGACGGAGCGCCCCTGCGAAAAACATAATAAGTCGGGTGACATACAGATCAGCAT :   86
consensus    TTCTTTACACTGCAGCACAGAGGCGTTACACGGACGGAGCGCCCCTGCGAAAAACATAATAAGTCGGGTGACATACAGATCAGCAT

                   *       100         *       120         *       140         *       160         *
CBS1-2_nox : CGCTTGTACGAAATACACATACCCCACGAATCTGCTCTCCTTCTGGGGATGCACGGCGCACATAACGACATCTTCTAACATTAGAA :  172
MS44c2_nox : CGCTTGTACGAAATACACATACCCCACGAATCTGCTCTCCTTCTGGGGATGCACGGCGCACATAACGACATCTTCTAACATTAGAA :  172
KH1a1_noxR : CGCTTGTACGAAATACACATACCCCACGAATCTGCTCTCCTTCTGGGGATGCACGGCGCACATAACGACATCTTCTAACATTAGAA :  172
CBS1-1_nox : CGCTTGTACGAAATACACATACCCCACGAATCTGCTCTCCTTCTGGGGATGCACGGCGCACATAACGACATCTTCTAACATTAGAA :  172
LF4f1_noxR : CGCTTGTACGAAATACACATACCCCACGAATCTGCTCTCCTTCTGGGGATGCACGGCGCACATAACGACATCTTCTAACATTAGAA :  172
LF4e1_noxR : CGCTTGTACGAAATACACATACCCCACGAATCTGCTCTCCTTCTGGGGATGCACGGCGCACATAACGACATCTTCTAACATTAGAA :  172
LF4d2_noxR : CGCTTGTACGAAATACACATACCCCACGAATCTGCTCTCCTTCTGGGGATGCACGGCGCACATAACGACATCTTCTAACATTAGAA :  172
LF4a1_noxR : CGCTTGTACGAAATACACATACCCCACGAATCTGCTCTCCTTCTGGGGATGCACGGCGCACATAACGACATCTTCTAACATTAGAA :  172
LF1e1_noxR : CGCTTGTACGAAATACACATACCCCACGAATCTGCTCTCCTTCTGGGGATGCACGGCGCACATAACGACATCTTCTAACATTAGAA :  172
KH1d1_noxR : CGCTTGTACGAAATACACATACCCCACGAATCTGCTCTCCTTCTGGGGATGCACGGCGCACATAACGACATCTTCTAACATTAGAA :  172
KH1c1_noxR : CGCTTGTACGAAATACACATACCCCACGAATCTGCTCTCCTTCTGGGGATGCACGGCGCACATAACGACATCTTCTAACATTAGAA :  172
KH1h1_noxR : CGCTTGTACGAAATACACATACCCCACGAATCTGCTCTCCTTCTGGGGATGCACGGCGCACATAACGACATCTTCTAACATTAGAA :  172
LF4a2_noxR : CGCTTGTACGAAATACACATACCCCACGAATCTGCTCTCCTTCTGGGGATGCACGGCGCACATAACGACATCTTCTAACATTAGAA :  172
LF4h1_noxR : CGCTTGTACGAAATACACATACCCCACGAATCTGCTCTCCTTCTGGGGATGCACGGCGCACATAACGACATCTTCTAACATTAGAA :  172
QM6a_cDNA  : CGCTT--------------------------------------------------------------------------------- :   91
QM6a_noxR  : CGCTTGTACGAAATACACATACCCCACGAATCTGCTCTCCTTCTGGGGATGCACGGCGCACATAACGACATCTTCTAACATTAGAA :  172
consensus    CGCTTgtacgaaatacacataccccacgaatctgctctccttctggggatgcacggcgcacataacgacatcttctaacattagaa

                  180         *       200         *       220         *       240         *       2
CBS1-2_nox : CAGCTTTTTCTCAGAGCTTGCGGAAGCAGACATTGTCAGGCTGATCAAAGCCAGAATTCGGGAACAGCTTTTGTCGGGCGATTCCA :  258
MS44c2_nox : CAGCTTTTTCTCAGAGCTTGCGGAAGCAGACATTGTCAGGCTGATCAAAGCCAGAATTCGGGAACAGCTTTTGTCGGGCGATTCCA :  258
KH1a1_noxR : CAGCTTTTTCTCAGAGCTTGCGGAAGCAGACATTGTCAGGCTGATCAAAGCCAGAATTCGGGAACAGCTTTTGTCGGGCGATTCCA :  258
CBS1-1_nox : CAGCTTTTTCTCAGAGCTTGCGGAAGCAGACATTGTCAGGCTGATCAAAGCCAGAATTCGGGARCAGCTTTTGTCGGGCGATTCCA :  258
LF4f1_noxR : CAGCTTTTTCTCAGAGCTTGCGGAAGCAGACATTGTCAGGCTGATCAAAGCCAGAATTCGGGAACAGCTTTTGTCGGGCGATTCCA :  258
LF4e1_noxR : CAGCTTTTTCTCAGAGCTTGCGGAAGCAGACATTGTCAGGCTGATCAAAGCCAGAATTCGGGAACAGCTTTTGTCGGGCGATTCCA :  258
LF4d2_noxR : CAGCTTTTTCTCAGAGCTTGCGGAAGCAGACATTGTCAGGCTGATCAAAGCCAGAATTCGGGAACAGCTTTTGTCGGGCGATTCCA :  258
LF4a1_noxR : CAGCTTTTTCTCAGAGCTTGCGGAAGCAGACATTGTCAGGCTGATCAAAGCCAGAATTCGGGAACAGCTTTTGTCGGGCGATTCCA :  258
LF1e1_noxR : CAGCTTTTTCTCAGAGCTTGCGGAAGCAGACATTGTCAGGCTGATCAAAGCCAGAATTCGGGAACAGCTTTTGTCGGGCGATTCCA :  258
KH1d1_noxR : CAGCTTTTTCTCAGAGCTTGCGGAAGCAGACATTGTCAGGCTGATCAAAGCCAGAATTCGGGAACAGCTTTTGTCGGGCGATTCCA :  258
KH1c1_noxR : CAGCTTTTTCTCAGAGCTTGCGGAAGCAGACATTGTCAGGCTGATCAAAGCCAGAATTCGGGAACAGCTTTTGTCGGGCGATTCCA :  258
KH1h1_noxR : CAGCTTTTTCTCAGAGCTTGCGGAAGCAGACATTGTCAGGCTGATCAAAGCCAGAATTCGGGAACAGCTTTTGTCGGGCGATTCCA :  258
LF4a2_noxR : CAGCTTTTTCTCAGAGCTTGCGGAAGCAGACATTGTCAGGCTGATCAAAGCCAGAATTCGGGAACAGCTTTTGTCGGGCGATTCCA :  258
LF4h1_noxR : CAGCTTTTTCTCAGAGCTTGCGGAAGCAGACATTGTCAGGCTGATCAAAGCCAGAATTCGGGAACAGCTTTTGTCGGGCGATTCCA :  258
QM6a_cDNA  : ---CTTTTTCTCAGAGCTTGCGGAAGCAGACATTGTCAGGTTGATCAAAGCCAGAATTCGGGAACAGCTTTTGTCGGGCAATTCCA :  174
QM6a_noxR  : CAGCTTTTTCTCAGAGCTTGCGGAAGCAGACATTGTCAGGTTGATCAAAGCCAGAATTCGGGAACAGCTTTTGTCGGGCAATTCCA :  258
consensus    cagCTTTTTCTCAGAGCTTGCGGAAGCAGACATTGTCAGGcTGATCAAAGCCAGAATTCGGGAACAGCTTTTGTCGGGCgATTCCA

             60         *       280         *       300         *       320         *       340
CBS1-2_nox : GGAGTCGGTGGCTTTGAGCATTATCACAGGATCCTTTCTTGGCTGGCGCCACACGCTTCGCCAAGGTCTAGCCCGCAGCTTGCACA :  344
MS44c2_nox : GGAGTCGGTGGCTTTGAGCATTATCACAGGATCCTTTCTTGGCTGGCGCCACACGCTTCGCCAAGGTCTAGCCCGCAGCTTGCACA :  344
KH1a1_noxR : GGAGTCGGTGGCTTTGAGCATTATCACAGGATCCTTTCTTGGCTGGCGCCACACGCTTCGCCAAGGTCTAGCCCGCAGCTTGCACA :  344
CBS1-1_nox : GGAGTCGGTGGCTTTGAGCATTATCACAGGATCCTTTCTTGGCTGGCGCCACACGCTTCGCCAAGGTCTAGCCCGCAGCTTGCACA :  344
LF4f1_noxR : GGAGTCGGTGGCTTTGAGCATTATCACAGGATCCTTTCTTGGCTGGCGCCACACGCTTCGCCAAGGTCTAGCCCGCAGCTTGCACA :  344
LF4e1_noxR : GGAGTCGGTGGCTTTGAGCATTATCACAGGATCCTTTCTTGGCTGGCGCCACACGCTTCGCCAAGGTCTAGCCCGCAGCTTGCACA :  344
LF4d2_noxR : GGAGTCGGTGGCTTTGAGCATTATCACAGGATCCTTTCTTGGCTGGCGCCACACGCTTCGCCAAGGTCTAGCCCGCAGCTTGCACA :  344
LF4a1_noxR : GGAGTCGGTGGCTTTGAGCATTATCACAGGATCCTTTCTTGGCTGGCGCCACACGCTTCGCCAAGGTCTAGCCCGCAGCTTGCACA :  344
LF1e1_noxR : GGAGTCGGTGGCTTTGAGCATTATCACAGGATCCTTTCTTGGCTGGCGCCACACGCTTCGCCAAGGTCTAGCCCGCAGCTTGCACA :  344
KH1d1_noxR : GGAGTCGGTGGCTTTGAGCATTATCACAGGATCCTTTCTTGGCTGGCGCCACACGCTTCGCCAAGGTCTAGCCCGCAGCTTGCACA :  344
KH1c1_noxR : GGAGTCGGTGGCTTTGAGCATTATCACAGGATCCTTTCTTGGCTGGCGCCACACGCTTCGCCAAGGTCTAGCCCGCAGCTTGCACA :  344
KH1h1_noxR : GGAGTCGGTGGCTTTGAGCATTATCACAGGATCCTTTCTTGGCTGGCGCCACACGCTTCGCCAAGGTCTAGCCCGCAGCTTGCACA :  344
LF4a2_noxR : GGAGTCGGTGGCTTTGAGCATTATCACAGGATCCTTTCTTGGCTGGCGCCACACGCTTCGCCAAGGTCTAGCCCGCAGCTTGCACA :  344
```

```
LF4h1_noxR : GGAGTCGGTGGCTTTGAGCATTATCACAGGATCCTTTCTTGGCTGGCGCCACACGCTTCGCCAAGGTCTAGCCCGCAGCTTGCACA :  344
QM6a_cDNA  : GGAGTCGGTGGCTTTGAGCATCATCACAGGATCCTCTCTTGGCTGGCGCCACACGCTTCGCCAAGGTCTAGCCCGCAGCTTGCACA :  260
QM6a_noxR  : GGAGTCGGTGGCTTTGAGCATCATCACAGGATCCTCTCTTGGCTGGCGCCACACGCTTCGCCAAGGTCTAGCCCGCAGCTTGCACA :  344
consensus    GGAGTCGGTGGCTTTGAGCATtATCACAGGATCCTtTCTTGGCTGGCGCCACACGCTTCGCCAAGGTCTAGCCCGCAGCTTGCACA

                    *       360         *       380         *       400         *       420         *
CBS1-2_nox : CTTTTCCGACCCAAAGCAAACAAAGGCGGGGGGTACCGACGTCTTTTTGGGATCCAAGAGGCTTCGTACTCTGCTCTGACAAGACA :  430
MS44c2_nox : CTTTTCCGACCCAAAGCAAACAAAGGCGGGGGGTACCGACGTCTTTTTGGGATCCAAGAGGCTTCGTACTCTGCTCTGACAAGACA :  430
KH1a1_noxR : CTTTTCCGACCCAAAGCAAACAAAGGCGGGGGGTACCGACGTCTTTTTGGGATCCAAGAGGCTTCGTACTCTGCTCTGACAAGACA :  430
CBS1-1_nox : CTTTTCCGACCCAAAGCAAACAAAGGCGGGGGGTACCGACGTCTTTTTGGGATCCAAGAGGCTTCGTACTCTGCTCTGACAAGACA :  430
LF4f1_noxR : CTTTTCCGACCCAAAGCAAACAAAGGCGGGGGGTACCGACGTCTTTTTGGGATCCAAGAGGCTTCGTACTCTGCTCTGACAAGACA :  430
LF4e1_noxR : CTTTTCCGACCCAAAGCAAACAAAGGCGGGGGGTACCGACGTCTTTTTGGGATCCAAGAGGCTTCGTACTCTGCTCTGACAAGACA :  430
LF4d2_noxR : CTTTTCCGACCCAAAGCAAACAAAGGCGGGGGGTACCGACGTCTTTTTGGGATCCAAGAGGCTTCGTACTCTGCTCTGACAAGACA :  430
LF4a1_noxR : CTTTTCCGACCCAAAGCAAACAAAGGCGGGGGGTACCGACGTCTTTTTGGGATCCAAGAGGCTTCGTACTCTGCTCTGACAAGACA :  430
LF1e1_noxR : CTTTTCCGACCCAAAGCAAACAAAGGCGGGGGGTACCGACGTCTTTTTGGGATCCAAGAGGCTTCGTACTCTGCTCTGACAAGACA :  430
KH1d1_noxR : CTTTTCCGACCCAAAGCAAACAAAGGCGGGGGGTACCGACGTCTTTTTGGGATCCAAGAGGCTTCGTACTCTGCTCTGACAAGACA :  430
KH1c1_noxR : CTTTTCCGACCCAAAGCAAACAAAGGCGGGGGGTACCGACGTCTTTTTGGGATCCAAGAGGCTTCGTACTCTGCTCTGACAAGACA :  430
KH1h1_noxR : CTTTTCCGACCCAAAGCAAACAAAGGCGGGGGGTACCGACGTCTTTTTGGGATCCAAGAGGCTTCGTACTCTGCTCTGACAAGACA :  430
LF4a2_noxR : CTTTTCCGACCCAAAGCAAACAAAGGCGGGGGGTACCGACGTCTTTTTGGGATCCAAGAGGCTTCGTACTCTGCTCTGACAAGACA :  430
LF4h1_noxR : CTTTTCCGACCCAAAGCAAACAAAGGCGGGGGGTACCGACGTCTTTTTGGGATCCAAGAGGCTTCGTACTCTGCTCTGACAAGACA :  430
QM6a_cDNA  : CTTTTCCGACCCAAAGCAAACAAAGGCGGGGGGTACCGACGTCTTTT-GGAATCCAAGAGGCTTCGTACTCTGCTCTGACAAGACA :  345
QM6a_noxR  : CTTTTCCGACCCAAAGCAAACAAAGGCGGGGGGTACCGACGTCTTTT-GGAATCCAAGAGGCTTCGTACTCTGCTCTGACAAGACA :  429
consensus    CTTTTCCGACCCAAAGCAAACAAAGGCGGGGGGTACCGACGTCTTTTtGGgATCCAAGAGGCTTCGTACTCTGCTCTGACAAGACA


                      440         *       460         *       480         *       500         *
CBS1-2_nox : CGTCGTTGCAATTTTGGCTGGAGCTCCTCTCCCGATAGGCCGTGGTATTCCTTTGTCTCTCTCAGCCCCAGCATTCTGAGGCGCCA :  516
MS44c2_nox : CGTCGTTGCAATTTTGGCTGGAGCTCCTCTCCCGATAGGCCGTGGTATTCCTTTGTCTCTCTCAGCCCCAGCATTCTGAGGCGCCA :  516
KH1a1_noxR : CGTCGTTGCAATTTTGGCTGGAGCTCCTCTCCCGATAGGCCGTGGTATTCCTTTGTCTCTCTCAGCCCCAGCATTCTGAGGCGCCA :  516
CBS1-1_nox : CGTCGTTGCAATTTTGGCTGGAGCTCCTCTCCCGATAGGCCGTGGTATTCCTTTGTCTCTCTCAGCCCCAGCATTCTGAGGCGCCA :  516
LF4f1_noxR : CGTCGTTGCAATTTTGGCTGGAGCTCCTCTCCCGATAGGCCGTGGTATTCCTTTGTCTCTCTCAGCCCCAGCATTCTGAGGCGCCA :  516
LF4e1_noxR : CGTCGTTGCAATTTTGGCTGGAGCTCCTCTCCCGATAGGCCGTGGTATTCCTTTGTCTCTCTCAGCCCCAGCATTCTGAGGCGCCA :  516
LF4d2_noxR : CGTCGTTGCAATTTTGGCTGGAGCTCCTCTCCCGATAGGCCGTGGTATTCCTTTGTCTCTCTCAGCCCCAGCATTCTGAGGCGCCA :  516
LF4a1_noxR : CGTCGTTGCAATTTTGGCTGGAGCTCCTCTCCCGATAGGCCGTGGTATTCCTTTGTCTCTCTCAGCCCCAGCATTCTGAGGCGCCA :  516
LF1e1_noxR : CGTCGTTGCAATTTTGGCTGGAGCTCCTCTCCCGATAGGCCGTGGTATTCCTTTGTCTCTCTCAGCCCCAGCATTCTGAGGCGCCA :  516
KH1d1_noxR : CGTCGTTGCAATTTTGGCTGGAGCTCCTCTCCCGATAGGCCGTGGTATTCCTTTGTCTCTCTCAGCCCCAGCATTCTGAGGCGCCA :  516
KH1c1_noxR : CGTCGTTGCAATTTTGGCTGGAGCTCCTCTCCCGATAGGCCGTGGTATTCCTTTGTCTCTCTCAGCCCCAGCATTCTGAGGCGCCA :  516
KH1h1_noxR : CGTCGTTGCAATTTTGGCTGGAGCTCCTCTCCCGATAGGCCGTGGTATTCCTTTGTCTCTCTCAGCCCCAGCATTCTGAGGCGCCA :  516
LF4a2_noxR : CGTCGTTGCAATTTTGGCTGGAGCTCCTCTCCCGATAGGCCGTGGTATTCCTTTGTCTCTCTCAGCCCCAGCATTCTGAGGCGCCA :  516
LF4h1_noxR : CGTCGTTGCAATTTTGGCTGGAGCTCCTCTCCCGATAGGCCGTGGTATTCCTTTGTCTCTCTCAGCCCCAGCATTCTGAGGCGCCA :  516
QM6a_cDNA  : CCTCGTTGCAATTTTGGCTGGAGCTCCTCTCCCGATAGGCCGTGGTATTCCTTTGTCTCTCTCAGCCCCAGCATTCTGAGGCGCCA :  431
QM6a_noxR  : CCTCGTTGCAATTTTGGCTGGAGCTCCTCTCCCGATAGGCCGTGGTATTCCTTTGTCTCTCTCAGCCCCAGCATTCTGAGGCGCCA :  515
consensus    CgTCGTTGCAATTTTGGCTGGAGCTCCTCTCCCGATAGGCCGTGGTATTCCTTTGTCTCTCTCAGCCCCAGCATTCTGAGGCGCCA


                 520         *       540         *       560         *       580         *       600
CBS1-2_nox : GCCCTTCCGTGGGCTGTAAATAGATCGTCCTCCATATACAATTGCCCAGCCAGCTACTCGACCGTCATCCTGTGCTTTCGCGCATA :  602
MS44c2_nox : GCCCTTCCGTGGGCTGTAAATAGATCGTCCTCCATATACAATTGCCCAGCCAGCTACTCGACCGTCATCCTGTGCTTTCGCGCATA :  602
KH1a1_noxR : GCCCTTCCGTGGGCTGTAAATAGATCGTCCTCCATATACAATTGCCCAGCCAGCTACTCGACCGTCATCCTGTGCTTTCGCGCATA :  602
CBS1-1_nox : GCCCTTCCGTGGGCTGTAAATAGATCGTCCTCCATATACAATTGCCCAGCCAGCTACTCGACCGTCATCCTGTGCTTTCGCGCATA :  602
LF4f1_noxR : GCCCTTCCGTGGGCTGTAAATAGATCGTCCTCCATATACAATTGCCCAGCCAGCTACTCGACCGTCATCCTGTGCTTTCGCGCATA :  602
LF4e1_noxR : GCCCTTCCGTGGGCTGTAAATAGATCGTCCTCCATATACAATTGCCCAGCCAGCTACTCGACCGTCATCCTGTGCTTTCGCGCATA :  602
LF4d2_noxR : GCCCTTCCGTGGGCTGTAAATAGATCGTCCTCCATATACAATTGCCCAGCCAGCTACTCGACCGTCATCCTGTGCTTTCGCGCATA :  602
LF4a1_noxR : GCCCTTCCGTGGGCTGTAAATAGATCGTCCTCCATATACAATTGCCCAGCCAGCTACTCGACCGTCATCCTGTGCTTTCGCGCATA :  602
LF1e1_noxR : GCCCTTCCGTGGGCTGTAAATAGATCGTCCTCCATATACAATTGCCCAGCCAGCTACTCGACCGTCATCCTGTGCTTTCGCGCATA :  602
KH1d1_noxR : GCCCTTCCGTGGGCTGTAAATAGATCGTCCTCCATATACAATTGCCCAGCCAGCTACTCGACCGTCATCCTGTGCTTTCGCGCATA :  602
KH1c1_noxR : GCCCTTCCGTGGGCTGTAAATAGATCGTCCTCCATATACAATTGCCCAGCCAGCTACTCGACCGTCATCCTGTGCTTTCGCGCATA :  602
KH1h1_noxR : GCCCTTCCGTGGGCTGTAAATAGATCGTCCTCCATATACAATTGCCCAGCCAGCTACTCGACCGTCATCCTGTGCTTTCGCGCATA :  602
LF4a2_noxR : GCCCTTCCGTGGGCTGTAAATAGATCGTCCTCCATATACAATTGCCCAGCCAGCTACTCGACCGTCATCCTGTGCTTTCGCGCATA :  602
LF4h1_noxR : GCCCTTCCGTGGGCTGTAAATAGATCGTCCTCCATATACAATTGCCCAGCCAGCTACTCGACCGTCATCCTGTGCTTTCGCGCATA :  602
QM6a_cDNA  : GCCCTTCCGTGGGCTGTAAATAGATCGTCCTCCATATACAATTGCCCAGCCAGCTACTCGACCGTCTTCCTGTGCTTTCGCGCATA :  517
QM6a_noxR  : GCCCTTCCGTGGGCTGTAAATAGATCGTCCTCCATATACAATTGCCCAGCCAGCTACTCGACCGTCTTCCTGTGCTTTCGCGCATA :  601
consensus    GCCCTTCCGTGGGCTGTAAATAGATCGTCCTCCATATACAATTGCCCAGCCAGCTACTCGACCGTCaTCCTGTGCTTTCGCGCATA


                      *       620         *       640         *       660         *       680
CBS1-2_nox : GTCGGGTACGATGTCTCTGAAACAGGTAAGAGAAGTCGGCCTCGTGGTCTTGTCGGAAGAAGGCGTGGCGTATGCTGATACCCTTG :  688
MS44c2_nox : GTCGGGTACGATGTCTCTGAAACAGGTAAGAGAAGTCGGCCTCGTGGTCTTGTCGGAAGAAGGCGTGGCGTATGCTGATACCCTTG :  688
KH1a1_noxR : GTCGGGTACGATGTCTCTGAAACAGGTAAGAGAAGTCGGCCTCGTGGTCTTGTCGGAAGAAGGCGTGGCGTATGCTGATACCCTTG :  688
CBS1-1_nox : GTCGGGTACGATGTCTCTGAAACAGGTAAGAGAAGTCGGCCTCGTGGTCTTGTCGGAAGAAGGCGTGGCGTATGCTGATACCCTTG :  688
LF4f1_noxR : GTCGGGTACGATGTCTCTGAAACAGGTAAGAGAAGTCGGCCTCGTGGTCTTGTCGGAAGAAGGCGTGGCGTATGCTGATACCCTTG :  688
LF4e1_noxR : GTCGGGTACGATGTCTCTGAAACAGGTAAGAGAAGTCGGCCTCGTGGTCTTGTCGGAAGAAGGCGTGGCGTATGCTGATACCCTTG :  688
LF4d2_noxR : GTCGGGTACGATGTCTCTGAAACAGGTAAGAGAAGTCGGCCTCGTGGTCTTGTCGGAAGAAGGCGTGGCGTATGCTGATACCCTTG :  688
LF4a1_noxR : GTCGGGTACGATGTCTCTGAAACAGGTAAGAGAAGTCGGCCTCGTGGTCTTGTCGGAAGAAGGCGTGGCGTATGCTGATACCCTTG :  688
LF1e1_noxR : GTCGGGTACGATGTCTCTGAAACAGGTAAGAGAAGTCGGCCTCGTGGTCTTGTCGGAAGAAGGCGTGGCGTATGCTGATACCCTTG :  688
KH1d1_noxR : GTCGGGTACGATGTCTCTGAAACAGGTAAGAGAAGTCGGCCTCGTGGTCTTGTCGGAAGAAGGCGTGGCGTATGCTGATACCCTTG :  688
```

```
KH1c1_noxR : GTCGGGTACGATGTCTCTGAAACAGGTAAGAGAAGTCGGCCTCGTGGTCTTGTCGGAAGAAGGCGTGGCGTATGCTGATACCCTTG :  688
KH1h1_noxR : GTCGGGTACGATGTCTCTGAAACAGGTAAGAGAAGTCGGCCTCGTGGTCTTGTCGGAAGAAGGCGTGGCGTATGCTGATACCCTTG :  688
LF4a2_noxR : GTCGGGTACGATGTCTCTGAAACAGGTAAGAGAAGTCGGCCTCGTGGTCTTGTCGGAAGAAGGCGTGGCGTATGCTGATACCCTTG :  688
LF4h1_noxR : GTCGGGTACGATGTCTCTGAAACAGGTAAGAGAAGTCGGCCTCGTGGTCTTGTCGGAAGAAGGCGTGGCGTATGCTGATACCCTTG :  688
QM6a_cDNA  : GTCGGGTACGATGTCTCTGAAACAGG------------------------------------------------------------ :  543
QM6a_noxR  : GTCGGGTACGATGTCTCTGAAACAGGTAAGAGAAGTCGGCCTCGTGGTCTTGTCGGAAGGCGTGGCGTATGCTGATACCCTTGGCT :  687
consensus    GTCGGGTACGATGTCTCTGAAACAGGtaagagaagtcggcctcgtggtcttgtcggaagaaggcgtggcgtatgctgatacccttg

              *       700         *       720         *       740         *       760         *
CBS1-2_nox : GCTTGTGTAGGAAATTGAGACCTGGGTCGCGGCCCTTGGTCGCTACGACAATAATGAGTTCGACGAAGCTCTTGCCGAGTTCGACA :  774
MS44c2_nox : GCTTGTGTAGGAAATTGAGACCTGGGTCGCGGCCCTTGGTCGCTACGACAATAATGAGTTCGACGAAGCTCTTGCCGAGTTCGACA :  774
KH1a1_noxR : GCTTGTGTAGGAAATTGAGACCTGGGTCGCGGCCCTTGGTCGCTACGACAATAATGAGTTCGACGAAGCTCTTGCCGAGTTCGACA :  774
CBS1-1_nox : GCTTGTGTAGGAAATTGAGACCTGGGTCGCGGCCCTTGGTCGCTACGACAATAATGAGTTCGACGAAGCTCTTGCCGAGTTCGACA :  774
LF4f1_noxR : GCTTGTGTAGGAAATTGAGACCTGGGTCGCGGCCCTTGGTCGCTACGACAATAATGAGTTCGACGAAGCTCTTGCCGAGTTCGACA :  774
LF4e1_noxR : GCTTGTGTAGGAAATTGAGACCTGGGTCGCGGCCCTTGGTCGCTACGACAATAATGAGTTCGACGAAGCTCTTGCCGAGTTCGACA :  774
LF4d2_noxR : GCTTGTGTAGGAAATTGAGACCTGGGTCGCGGCCCTTGGTCGCTACGACAATAATGAGTTCGACGAAGCTCTTGCCGAGTTCGACA :  774
LF4a1_noxR : GCTTGTGTAGGAAATTGAGACCTGGGTCGCGGCCCTTGGTCGCTACGACAATAATGAGTTCGACGAAGCTCTTGCCGAGTTCGACA :  774
LF1e1_noxR : GCTTGTGTAGGAAATTGAGACCTGGGTCGCGGCCCTTGGTCGCTACGACAATAATGAGTTCGACGAAGCTCTTGCCGAGTTCGACA :  774
KH1d1_noxR : GCTTGTGTAGGAAATTGAGACCTGGGTCGCGGCCCTTGGTCGCTACGACAATAATGAGTTCGACGAAGCTCTTGCCGAGTTCGACA :  774
KH1c1_noxR : GCTTGTGTAGGAAATTGAGACCTGGGTCGCGGCCCTTGGTCGCTACGACAATAATGAGTTCGACGAAGCTCTTGCCGAGTTCGACA :  774
KH1h1_noxR : GCTTGTGTAGGAAATTGAGACCTGGGTCGCGGCCCTTGGTCGCTACGACAATAATGAGTTCGACGAAGCTCTTGCCGAGTTCGACA :  774
LF4a2_noxR : GCTTGTGTAGGAAATTGAGACCTGGGTCGCGGCCCTTGGTCGCTACGACAATAATGAGTTCGACGAAGCTCTTGCCGAGTTCGACA :  774
LF4h1_noxR : GCTTGTGTAGGAAATTGAGACCTGGGTCGCGGCCCTTGGTCGCTACGACAATAATGAGTTCGACGAAGCTCTTGCCGAGTTCGACA :  774
QM6a_cDNA  : -----------AAATTGAGACCTGGGTCGCGGCCCTTGGTCGCTACGACAATAATGAGTTCGACGAAGCTCTTGCCGAGTTCGACA :  618
QM6a_noxR  : TGTGTAGG---AAATTGAGACCTGGGTCGCGGCCCTTGGTCGCTACGACAATAATGAGTTCGACGAAGCTCTTGCCGAGTTCGACA :  770
consensus    gcttgtgtaggAAATTGAGACCTGGGTCGCGGCCCTTGGTCGCTACGACAATAATGAGTTCGACGAAGCTCTTGCCGAGTTCGACA

                780         *       800         *       820         *       840         *       860
CBS1-2_nox : AGATTGGCGATACAAGCAAGATTCTCTTCAACATGGGTGTCATCCACGCCACTCTGGGCGAGCACGAGAAAGCTGTGGGTTGTGCC :  860
MS44c2_nox : AGATTGGCGATACAAGCAAGATTCTCTTCAACATGGGTGTCATCCACGCCACTCTGGGCGAGCACGAGAAAGCTGTGGGTTGTGCC :  860
KH1a1_noxR : AGATTGGCGATACAAGCAAGATTCTCTTCAACATGGGTGTCATCCACGCCACTCTGGGCGAGCACGAGAAAGCTGTGGGTTGTGCC :  860
CBS1-1_nox : AGATTGGCGATACAAGCAAGATTCTCTTCAACATGGGTGTCATCCACGCCACTCTGGGCGAGCACGAGAAAGCTGTGGGTTGTGCC :  860
LF4f1_noxR : AGATTGGCGATACAAGCAAGATTCTCTTCAACATGGGTGTCATCCACGCCACTCTGGGCGAGCACGAGAAAGCTGTGGGTTGTGCC :  860
LF4e1_noxR : AGATTGGCGATACAAGCAAGATTCTCTTCAACATGGGTGTCATCCACGCCACTCTGGGCGAGCACGAGAAAGCTGTGGGTTGTGCC :  860
LF4d2_noxR : AGATTGGCGATACAAGCAAGATTCTCTTCAACATGGGTGTCATCCACGCCACTCTGGGCGAGCACGAGAAAGCTGTGGGTTGTGCC :  860
LF4a1_noxR : AGATTGGCGATACAAGCAAGATTCTCTTCAACATGGGTGTCATCCACGCCACTCTGGGCGAGCACGAGAAAGCTGTGGGTTGTGCC :  860
LF1e1_noxR : AGATTGGCGATACAAGCAAGATTCTCTTCAACATGGGTGTCATCCACGCCACTCTGGGCGAGCACGAGAAAGCTGTGGGTTGTGCC :  860
KH1d1_noxR : AGATTGGCGATACAAGCAAGATTCTCTTCAACATGGGTGTCATCCACGCCACTCTGGGCGAGCACGAGAAAGCTGTGGGTTGTGCC :  860
KH1c1_noxR : AGATTGGCGATACAAGCAAGATTCTCTTCAACATGGGTGTCATCCACGCCACTCTGGGCGAGCACGAGAAAGCTGTGGGTTGTGCC :  860
KH1h1_noxR : AGATTGGCGATACAAGCAAGATTCTCTTCAACATGGGTGTCATCCACGCCACTCTGGGCGAGCACGAGAAAGCTGTGGGTTGTGCC :  860
LF4a2_noxR : AGATTGGCGATACAAGCAAGATTCTCTTCAACATGGGTGTCATCCACGCCACTCTGGGCGAGCACGAGAAAGCTGTGGGTTGTGCC :  860
LF4h1_noxR : AGATTGGCGATACAAGCAAGATTCTCTTCAACATGGGTGTCATCCACGCCACTCTGGGCGAGCACGAGAAAGCTGTGGGTTGTGCC :  860
QM6a_cDNA  : AGATTGGCGATACAAGCAAGATTCTCTTCAACATGGGTGTCATCCACGCCACTCTGGGCGAGCACGAGAAAGCTGT---------- :  694
QM6a_noxR  : AGATTGGCGATACAAGCAAGATTCTCTTCAACATGGGTGTCATCCACGCCACTCTGGGCGAGCACGAGAAAGCTGTGGGTTGTGCC :  856
consensus    AGATTGGCGATACAAGCAAGATTCTCTTCAACATGGGTGTCATCCACGCCACTCTGGGCGAGCACGAGAAAGCTGTgggttgtgcc

                      *       880         *       900         *       920         *       940
CBS1-2_nox : ATATTCCCAGCACCGCCTCGACCCTGCCATGCTAACACATGCTGTGCGCAATACCTAGGTCGAGTGTTACCAACGAGCCATCCGAT :  946
MS44c2_nox : ATATTCCCAGCACCGCCTCGACCCTGCCATGCTAACACATGCTGTGCGCAATACCTAGGTCGAGTGTTACCAACGAGCCATCCGAT :  946
KH1a1_noxR : ATATTCCCAGCACCGCCTCGACCCTGCCATGCTAACACATGCTGTGCGCAATACCTAGGTCGAGTGTTACCAACGAGCCATCCGAT :  946
CBS1-1_nox : ATATTCCCAGCACCGCCTCGACCCTGCCATGCTAACACATGCTGTGCGCAATACCTAGGTCGAGTGTTACCAACGAGCCATCCGAT :  946
LF4f1_noxR : ATATTCCCAGCACCGCCTCGACCCTGCCATGCTAACACATGCTGTGCGCAATACCTAGGTCGAGTGTTACCAACGAGCCATCCGAT :  946
LF4e1_noxR : ATATTCCCAGCACCGCCTCGACCCTGCCATGCTAACACATGCTGTGCGCAATACCTAGGTCGAGTGTTACCAACGAGCCATCCGAT :  946
LF4d2_noxR : ATATTCCCAGCACCGCCTCGACCCTGCCATGCTAACACATGCTGTGCGCAATACCTAGGTCGAGTGTTACCAACGAGCCATCCGAT :  946
LF4a1_noxR : ATATTCCCAGCACCGCCTCGACCCTGCCATGCTAACACATGCTGTGCGCAATACCTAGGTCGAGTGTTACCAACGAGCCATCCGAT :  946
LF1e1_noxR : ATATTCCCAGCACCGCCTCGACCCTGCCATGCTAACACATGCTGTGCGCAATACCTAGGTCGAGTGTTACCAACGAGCCATCCGAT :  946
KH1d1_noxR : ATATTCCCAGCACCGCCTCGACCCTGCCATGCTAACACATGCTGTGCGCAATACCTAGGTCGAGTGTTACCAACGAGCCATCCGAT :  946
KH1c1_noxR : ATATTCCCAGCACCGCCTCGACCCTGCCATGCTAACACATGCTGTGCGCAATACCTAGGTCGAGTGTTACCAACGAGCCATCCGAT :  946
KH1h1_noxR : ATATTCCCAGCACCGCCTCGACCCTGCCATGCTAACACATGCTGTGCGCAATACCTAGGTCGAGTGTTACCAACGAGCCATCCGAT :  946
LF4a2_noxR : ATATTCCCAGCACCGCCTCGACCCTGCCATGCTAACACATGCTGTGCGCAATACCTAGGTCGAGTGTTACCAACGAGCCATCCGAT :  946
LF4h1_noxR : ATATTCCCAGCACCGCCTCGACCCTGCCATGCTAACACATGCTGTGCGCAATACCTAGGTCGAGTGTTACCAACGAGCCATCCGAT :  946
QM6a_cDNA  : ------------------------------------------------------------CGAGTGTTACCAACGAGCCATCCGAT :  720
QM6a_noxR  : ATATTCCCAGCACCGCCTCGACCCTGCCATGCTAACACATGCTGTGCGCAATACCTAGGTCGAGTGTTACCAACGAGCCATCCGAT :  942
consensus    atattcccagcaccgcctcgaccctgccatgctaacacatgctgtgcgcaatacctaggtCGAGTGTTACCAACGAGCCATCCGAT

                *       960         *       980         *      1000         *      1020         *
CBS1-2_nox : TAGATCAGTACCTGGCCGTCGCCTACTTCCAGCAGGGCGTGTCCAATTTCTTGCTGGGTGACTTCGAAGAGGCCCTCGCCAACTTC : 1032
MS44c2_nox : TAGATCAGTACCTGGCCGTCGCCTACTTCCAGCAGGGCGTGTCCAATTTCTTGCTGGGTGACTTCGAAGAGGCCCTCGCCAACTTC : 1032
KH1a1_noxR : TAGATCAGTACCTGGCCGTCGCCTACTTCCAGCAGGGCGTGTCCAATTTCTTGCTGGGTGACTTCGAAGAGGCCCTCGCCAACTTC : 1032
CBS1-1_nox : TAGATCAGTACCTGGCCGTCGCCTACTTCCAGCAGGGCGTGTCCAATTTCTTGCTGGGTGACTTCGAAGAGGCCCTCGCCAACTTC : 1032
LF4f1_noxR : TAGATCAGTACCTGGCCGTCGCCTACTTCCAGCAGGGCGTGTCCAATTTCTTGCTGGGTGACTTCGAAGAGGCCCTCGCCAACTTC : 1032
LF4e1_noxR : TAGATCAGTACCTGGCCGTCGCCTACTTCCAGCAGGGCGTGTCCAATTTCTTGCTGGGTGACTTCGAAGAGGCCCTCGCCAACTTC : 1032
LF4d2_noxR : TAGATCAGTACCTGGCCGTCGCCTACTTCCAGCAGGGCGTGTCCAATTTCTTGCTGGGTGACTTCGAAGAGGCCCTCGCCAACTTC : 1032
```

```
LF4a1_noxR : TAGATCAGTACCTGGCCGTCGCCTACTTCCAGCAGGGCGTGTCCAATTTCTTGCTGGGTGACTTCGAAGAGGCCCTCGCCAACTTC : 1032
LF1e1_noxR : TAGATCAGTACCTGGCCGTCGCCTACTTCCAGCAGGGCGTGTCCAATTTCTTGCTGGGTGACTTCGAAGAGGCCCTCGCCAACTTC : 1032
KH1d1_noxR : TAGATCAGTACCTGGCCGTCGCCTACTTCCAGCAGGGCGTGTCCAATTTCTTGCTGGGTGACTTCGAAGAGGCCCTCGCCAACTTC : 1032
KH1c1_noxR : TAGATCAGTACCTGGCCGTCGCCTACTTCCAGCAGGGCGTGTCCAATTTCTTGCTGGGTGACTTCGAAGAGGCCCTCGCCAACTTC : 1032
KH1h1_noxR : TAGATCAGTACCTGGCCGTCGCCTACTTCCAGCAGGGCGTGTCCAATTTCTTGCTGGGTGACTTCGAAGAGGCCCTCGCCAACTTC : 1032
LF4a2_noxR : TAGATCAGTACCTGGCCGTCGCCTACTTCCAGCAGGGCGTGTCCAATTTCTTGCTGGGTGACTTCGAAGAGGCCCTCGCCAACTTC : 1032
LF4h1_noxR : TAGATCAGTACCTGGCCGTCGCCTACTTCCAGCAGGGCGTGTCCAATTTCTTGCTGGGTGACTTCGAAGAGGCCCTCGCCAACTTC : 1032
QM6a_cDNA  : TAGATCAGTACCTGGCCGTCGCCTACTTCCAACAAGGCGTGTCCAACTTTTTGCTGGGTGACTTCGAAGAGGCCCTCGCCAACTTC :  806
QM6a_noxR  : TAGATCAGTACCTGGCCGTCGCCTACTTCCAACAAGGCGTGTCCAACTTTTTGCTGGGTGACTTCGAAGAGGCCCTCGCCAACTTC : 1028
consensus    TAGATCAGTACCTGGCCGTCGCCTACTTCCAgCAgGGCGTGTCCAAtTTcTTGCTGGGTGACTTCGAAGAGGCCCTCGCCAACTTC


                1040         *       1060         *       1080         *       1100         *       11
CBS1-2_nox : AACGACACGCTGCTGTATCTGCGAGGCAACAGCGTCATCGACTATGCCCAGCTCGGTCTTCTGTTCAAACTGTACTCGTGCGAGGT : 1118
MS44c2_nox : AACGACACGCTGCTGTATCTGCGAGGCAACAGCGTCATCGACTATGCCCAGCTCGGTCTTCTGTTCAAACTGTACTCGTGCGAGGT : 1118
KH1a1_noxR : AACGACACGCTGCTGTATCTGCGAGGCAACAGCGTCATCGACTATGCCCAGCTCGGTCTTCTGTTCAAACTGTACTCGTGCGAGGT : 1118
CBS1-1_nox : AACGACACGCTGCTGTATCTGCGAGGCAACAGCGTCATCGACTATGCCCAGCTCGGTCTTCTGTTCAAACTGTACTCGTGCGAGGT : 1118
LF4f1_noxR : AACGACACGCTGCTGTATCTGCGAGGCAACAGCGTCATCGACTATGCCCAGCTCGGTCTTCTGTTCAAACTGTACTCGTGCGAGGT : 1118
LF4e1_noxR : AACGACACGCTGCTGTATCTGCGAGGCAACAGCGTCATCGACTATGCCCAGCTCGGTCTTCTGTTCAAACTGTACTCGTGCGAGGT : 1118
LF4d2_noxR : AACGACACGCTGCTGTATCTGCGAGGCAACAGCGTCATCGACTATGCCCAGCTCGGTCTTCTGTTCAAACTGTACTCGTGCGAGGT : 1118
LF4a1_noxR : AACGACACGCTGCTGTATCTGCGAGGCAACAGCGTCATCGACTATGCCCAGCTCGGTCTTCTGTTCAAACTGTACTCGTGCGAGGT : 1118
LF1e1_noxR : AACGACACGCTGCTGTATCTGCGAGGCAACAGCGTCATCGACTATGCCCAGCTCGGTCTTCTGTTCAAACTGTACTCGTGCGAGGT : 1118
KH1d1_noxR : AACGACACGCTGCTGTATCTGCGAGGCAACAGCGTCATCGACTATGCCCAGCTCGGTCTTCTGTTCAAACTGTACTCGTGCGAGGT : 1118
KH1c1_noxR : AACGACACGCTGCTGTATCTGCGAGGCAACAGCGTCATCGACTATGCCCAGCTCGGTCTTCTGTTCAAACTGTACTCGTGCGAGGT : 1118
KH1h1_noxR : AACGACACGCTGCTGTATCTGCGAGGCAACAGCGTCATCGACTATGCCCAGCTCGGTCTTCTGTTCAAACTGTACTCGTGCGAGGT : 1118
LF4a2_noxR : AACGACACGCTGCTGTATCTGCGAGGCAACAGCGTCATCGACTATGCCCAGCTCGGTCTTCTGTTCAAACTGTACTCGTGCGAGGT : 1118
LF4h1_noxR : AACGACACGCTGCTGTATCTGCGAGGCAACAGCGTCATCGACTATGCCCAGCTCGGTCTTCTGTTCAAACTGTACTCGTGCGAGGT : 1118
QM6a_cDNA  : AACGACACGCTGCTGTATCTGCGAGGCAACAGCGTCATCGACTATGCCCAGCTCGGCCTTCTGTTCAAACTGTACTCGTGCGAGGT :  892
QM6a_noxR  : AACGACACGCTGCTGTATCTGCGAGGCAACAGCGTCATCGACTATGCCCAGCTCGGCCTTCTGTTCAAACTGTACTCGTGCGAGGT : 1114
consensus    AACGACACGCTGCTGTATCTGCGAGGCAACAGCGTCATCGACTATGCCCAGCTCGGtCTTCTGTTCAAACTGTACTCGTGCGAGGT


             20         *       1140         *       1160         *       1180         *       1200
CBS1-2_nox : CCTGTTCAACCGAGGATTATGCTACATCTACCTGCAGCAGAAGGAGGCGGGCATGCAGGATCTGGCGTATGCTGTTAAGGAGAAGG : 1204
MS44c2_nox : CCTGTTCAACCGAGGATTATGCTACATCTACCTGCAGCAGAAGGAGGCGGGCATGCAGGATCTGGCGTATGCTGTTAAGGAGAAGG : 1204
KH1a1_noxR : CCTGTTCAACCGAGGATTATGCTACATCTACCTGCAGCAGAAGGAGGCGGGCATGCAGGATCTGGCGTATGCTGTTAAGGAGAAGG : 1204
CBS1-1_nox : CCTGTTCAACCGAGGATTATGCTACATCTACCTGCAGCAGAAGGAGGCGGGCATGCAGGATCTGGCGTATGCTGTTAAGGAGAAGG : 1204
LF4f1_noxR : CCTGTTCAACCGAGGATTATGCTACATCTACCTGCAGCAGAAGGAGGCGGGCATGCAGGATCTGGCGTATGCTGTTAAGGAGAAGG : 1204
LF4e1_noxR : CCTGTTCAACCGAGGATTATGCTACATCTACCTGCAGCAGAAGGAGGCGGGCATGCAGGATCTGGCGTATGCTGTTAAGGAGAAGG : 1204
LF4d2_noxR : CCTGTTCAACCGAGGATTATGCTACATCTACCTGCAGCAGAAGGAGGCGGGCATGCAGGATCTGGCGTATGCTGTTAAGGAGAAGG : 1204
LF4a1_noxR : CCTGTTCAACCGAGGATTATGCTACATCTACCTGCAGCAGAAGGAGGCGGGCATGCAGGATCTGGCGTATGCTGTTAAGGAGAAGG : 1204
LF1e1_noxR : CCTGTTCAACCGAGGATTATGCTACATCTACCTGCAGCAGAAGGAGGCGGGCATGCAGGATCTGGCGTATGCTGTTAAGGAGAAGG : 1204
KH1d1_noxR : CCTGTTCAACCGAGGATTATGCTACATCTACCTGCAGCAGAAGGAGGCGGGCATGCAGGATCTGGCGTATGCTGTTAAGGAGAAGG : 1204
KH1c1_noxR : CCTGTTCAACCGAGGATTATGCTACATCTACCTGCAGCAGAAGGAGGCGGGCATGCAGGATCTGGCGTATGCTGTTAAGGAGAAGG : 1204
KH1h1_noxR : CCTGTTCAACCGAGGATTATGCTACATCTACCTGCAGCAGAAGGAGGCGGGCATGCAGGATCTGGCGTATGCTGTTAAGGAGAAGG : 1204
LF4a2_noxR : CCTGTTCAACCGAGGATTATGCTACATCTACCTGCAGCAGAAGGAGGCGGGCATGCAGGATCTGGCGTATGCTGTTAAGGAGAAGG : 1204
LF4h1_noxR : CCTGTTCAACCGAGGATTATGCTACATCTACCTGCAGCAGAAGGAGGCGGGCATGCAGGATCTGGCGTATGCTGTTAAGGAGAAGG : 1204
QM6a_cDNA  : CCTGTTCAACCGAGGATTATGCTACATCTACCTGCAGCAGAAGGAGGCGGGCATGCAGGATCTGGCGTATGCTGTTAAGGAGAAGG :  978
QM6a_noxR  : CCTGTTCAACCGAGGATTATGCTACATCTACCTGCAGCAGAAGGAGGCGGGCATGCAGGATCTGGCGTATGCTGTTAAGGAGAAGG : 1200
consensus    CCTGTTCAACCGAGGATTATGCTACATCTACCTGCAGCAGAAGGAGGCGGGCATGCAGGATCTGGCGTATGCTGTTAAGGAGAAGG


                  *       1220         *       1240         *       1260         *       1280         *
CBS1-2_nox : TTGTCGAAGACCACAACGTCATTGACGAGGCCATTCGCGAGGAAGCAGAGGTGAGGGCTCTCGAACGTTCCCGTGGCCAAAGACAC : 1290
MS44c2_nox : TTGTCGAAGACCACAACGTCATTGACGAGGCCATTCGCGAGGAAGCAGAGGTGAGGGCTCTCGAACGTTCCCGTGGCCAAAGACAC : 1290
KH1a1_noxR : TTGTCGAAGACCACAACGTCATTGACGAGGCCATTCGCGAGGAAGCAGAGGTGAGGGCTCTCGAACGTTCCCGTGGCCAAAGACAC : 1290
CBS1-1_nox : TTGTCGAAGACCACAACGTCATTGACGAGGCCATTCGCGAGGAAGCAGAGGTGAGGGCTCTCGAACGTTCCCGTGGCCAAAGACAC : 1290
LF4f1_noxR : TTGTCGAAGACCACAACGTCATTGACGAGGCCATTCGCGAGGAAGCAGAGGTGAGGGCTCTCGAACGTTCCCGTGGCCAAAGACAC : 1290
LF4e1_noxR : TTGTCGAAGACCACAACGTCATTGACGAGGCCATTCGCGAGGAAGCAGAGGTGAGGGCTCTCGAACGTTCCCGTGGCCAAAGACAC : 1290
LF4d2_noxR : TTGTCGAAGACCACAACGTCATTGACGAGGCCATTCGCGAGGAAGCAGAGGTGAGGGCTCTCGAACGTTCCCGTGGCCAAAGACAC : 1290
LF4a1_noxR : TTGTCGAAGACCACAACGTCATTGACGAGGCCATTCGCGAGGAAGCAGAGGTGAGGGCTCTCGAACGTTCCCGTGGCCAAAGACAC : 1290
LF1e1_noxR : TTGTCGAAGACCACAACGTCATTGACGAGGCCATTCGCGAGGAAGCAGAGGTGAGGGCTCTCGAACGTTCCCGTGGCCAAAGACAC : 1290
KH1d1_noxR : TTGTCGAAGACCACAACGTCATTGACGAGGCCATTCGCGAGGAAGCAGAGGTGAGGGCTCTCGAACGTTCCCGTGGCCAAAGACAC : 1290
KH1c1_noxR : TTGTCGAAGACCACAACGTCATTGACGAGGCCATTCGCGAGGAAGCAGAGGTGAGGGCTCTCGAACGTTCCCGTGGCCAAAGACAC : 1290
KH1h1_noxR : TTGTCGAAGACCACAACGTCATTGACGAGGCCATTCGCGAGGAAGCAGAGGTGAGGGCTCTCGAACGTTCCCGTGGCCAAAGACAC : 1290
LF4a2_noxR : TTGTCGAAGACCACAACGTCATTGACGAGGCCATTCGCGAGGAAGCAGAGGTGAGGGCTCTCGAACGTTCCCGTGGCCAAAGACAC : 1290
LF4h1_noxR : TTGTCGAAGACCACAACGTCATTGACGAGGCCATTCGCGAGGAAGCAGAGGTGAGGGCTCTCGAACGTTCCCGTGGCCAAAGACAC : 1290
QM6a_cDNA  : TTGTCGAAGATCACAACGTCATTGACGAGGCCATTCGCGAGGAAGCAGAGG----------------------------------- : 1029
QM6a_noxR  : TTGTCGAAGATCACAACGTCATTGACGAGGCCATTCGCGAGGAAGCAGAGGTGAGGACTCTCGAACGTTTCCGTGGTCAAAGACAC : 1286
consensus    TTGTCGAAGAcCACAACGTCATTGACGAGGCCATTCGCGAGGAAGCAGAGGtgagggctctcgaacgttcccgtggccaaagacac


                  1300         *       1320         *       1340         *       1360         *
CBS1-2_nox : CGGCTGACTTGCGTTACAACAGGGCTACACTGTGTTTTCCATCCCCGTGGGCGTCGTATATCGACCAAACGAAGCCAAGGTTCGCA : 1376
MS44c2_nox : CGGCTGACTTGCGTTACAACAGGGCTACACTGTGTTTTCCATCCCCGTGGGCGTCGTATATCGACCAAACGAAGCCAAGGTTCGCA : 1376
KH1a1_noxR : CGGCTGACTTGCGTTACAACAGGGCTACACTGTGTTTTCCATCCCCGTGGGCGTCGTATATCGACCAAACGAAGCCAAGGTTCGCA : 1376
```

```
CBS1-1_nox : CGGCTGACTTGCGTTACAACAGGGCTACACTGTGTTTTCCATCCCCGTGGGCGTCGTATATCGACCAAACGAAGCCAAGGTTCGCA : 1376
LF4f1_noxR : CGGCTGACTTGCGTTACAACAGGGCTACACTGTGTTTTCCATCCCCGTGGGCGTCGTATATCGACCAAACGAAGCCAAGGTTCGCA : 1376
LF4e1_noxR : CGGCTGACTTGCGTTACAACAGGGCTACACTGTGTTTTCCATCCCCGTGGGCGTCGTATATCGACCAAACGAAGCCAAGGTTCGCA : 1376
LF4d2_noxR : CGGCTGACTTGCGTTACAACAGGGCTACACTGTGTTTTCCATCCCCGTGGGCGTCGTATATCGACCAAACGAAGCCAAGGTTCGCA : 1376
LF4a1_noxR : CGGCTGACTTGCGTTACAACAGGGCTACACTGTGTTTTCCATCCCCGTGGGCGTCGTATATCGACCAAACGAAGCCAAGGTTCGCA : 1376
LF1e1_noxR : CGGCTGACTTGCGTTACAACAGGGCTACACTGTGTTTTCCATCCCCGTGGGCGTCGTATATCGACCAAACGAAGCCAAGGTTCGCA : 1376
KH1d1_noxR : CGGCTGACTTGCGTTACAACAGGGCTACACTGTGTTTTCCATCCCCGTGGGCGTCGTATATCGACCAAACGAAGCCAAGGTTCGCA : 1376
KH1c1_noxR : CGGCTGACTTGCGTTACAACAGGGCTACACTGTGTTTTCCATCCCCGTGGGCGTCGTATATCGACCAAACGAAGCCAAGGTTCGCA : 1376
KH1h1_noxR : CGGCTGACTTGCGTTACAACAGGGCTACACTGTGTTTTCCATCCCCGTGGGCGTCGTATATCGACCAAACGAAGCCAAGGTTCGCA : 1376
LF4a2_noxR : CGGCTGACTTGCGTTACAACAGGGCTACACTGTGTTTTCCATCCCCGTGGGCGTCGTATATCGACCAAACGAAGCCAAGGTTCGCA : 1376
LF4h1_noxR : CGGCTGACTTGCGTTACAACAGGGCTACACTGTGTTTTCCATCCCCGTGGGCGTCGTATATCGACCAAACGAAGCCAAGGTTCGCA : 1376
QM6a_cDNA  : -----------------------GATACACAGTGTTTTCCATTCCCGTGGGCGTCGTATATCGACCAAACGAAGCCAAGGTTCGCA : 1092
QM6a_noxR  : TGGCTGACTTGCGTTACAACAGGGATACACAGTGTTTTCCATTCCCGTGGGCGTCGTATATCGACCAAACGAAGCCAAGGTTCGCA : 1372
consensus    cggctgacttgcgttacaacaggGcTACACtGTGTTTTCCATcCCCGTGGGCGTCGTATATCGACCAAACGAAGCCAAGGTTCGCA

               1380         *       1400         *       1420         *       1440         *       1460
CBS1-2_nox : ATTTGAAGACCAAGGACTATCTTGGCAAGGCCAAGCTGGTTGCAGCCTCGGATCGATCCAATGCATTTACTGGATTTGCCGGTTCC : 1462
MS44c2_nox : ATTTGAAGACCAAGGACTATCTTGGCAAGGCCAAGCTGGTTGCAGCCTCGGATCGATCCAATGCATTTACTGGATTTGCCGGTTCC : 1462
KH1a1_noxR : ATTTGAAGACCAAGGACTATCTTGGCAAGGCCAAGCTGGTTGCAGCCTCGGATCGATCCAATGCATTTACTGGATTTGCCGGTTCC : 1462
CBS1-1_nox : ATTTGAAGACCAAGGACTATCTTGGCAAGGCCAAGCTGGTTGCAGCCTCGGATCGATCCAATGCATTTACTGGATTTGCCGGTTCC : 1462
LF4f1_noxR : ATTTGAAGACCAAGGACTATCTTGGCAAGGCCAAGCTGGTTGCAGCCTCGGATCGATCCAATGCATTTACTGGATTTGCCGGTTCC : 1462
LF4e1_noxR : ATTTGAAGACCAAGGACTATCTTGGCAAGGCCAAGCTGGTTGCAGCCTCGGATCGATCCAATGCATTTACTGGATTTGCCGGTTCC : 1462
LF4d2_noxR : ATTTGAAGACCAAGGACTATCTTGGCAAGGCCAAGCTGGTTGCAGCCTCGGATCGATCCAATGCATTTACTGGATTTGCCGGTTCC : 1462
LF4a1_noxR : ATTTGAAGACCAAGGACTATCTTGGCAAGGCCAAGCTGGTTGCAGCCTCGGATCGATCCAATGCATTTACTGGATTTGCCGGTTCC : 1462
LF1e1_noxR : ATTTGAAGACCAAGGACTATCTTGGCAAGGCCAAGCTGGTTGCAGCCTCGGATCGATCCAATGCATTTACTGGATTTGCCGGTTCC : 1462
KH1d1_noxR : ATTTGAAGACCAAGGACTATCTTGGCAAGGCCAAGCTGGTTGCAGCCTCGGATCGATCCAATGCATTTACTGGATTTGCCGGTTCC : 1462
KH1c1_noxR : ATTTGAAGACCAAGGACTATCTTGGCAAGGCCAAGCTGGTTGCAGCCTCGGATCGATCCAATGCATTTACTGGATTTGCCGGTTCC : 1462
KH1h1_noxR : ATTTGAAGACCAAGGACTATCTTGGCAAGGCCAAGCTGGTTGCAGCCTCGGATCGATCCAATGCATTTACTGGATTTGCCGGTTCC : 1462
LF4a2_noxR : ATTTGAAGACCAAGGACTATCTTGGCAAGGCCAAGCTGGTTGCAGCCTCGGATCGATCCAATGCATTTACTGRATTTGCCGKTTCC : 1462
LF4h1_noxR : ATTTGAAGACCAAGGACTATCTTGGCAAGGCCAAGCTGGTTGCAGCCTCGGATCGATCCAATGCATTTACTGGATTTGCCGGTTCC : 1462
QM6a_cDNA  : ATCTCAAAACCAAGGACTATCTCGGCAAGGCCAAGCTGGTTGCAGCCTCGGATCGATCCAATGCATTCACTGGATTCGCCGGTTCC : 1178
QM6a_noxR  : ATCTCAAAACCAAGGACTATCTCGGCAAGGCCAAGCTGGTTGCAGCCTCGGATCGATCCAATGCATTCACTGGATTCGCCGGTTCC : 1458
consensus    ATtTgAAgACCAAGGACTATCTtGGCAAGGCCAAGCTGGTTGCAGCCTCGGATCGATCCAATGCATTtACTGGATTtGCCGGTTCC

                    *       1480         *       1500         *       1520         *       1540
CBS1-2_nox : GAGATCAAAAATGTGAGCACCTGTGCCCCCAGCAATGTGTTTCTCTTGCTTATACACCCCACCTCTAGGCCTTGACGGAAGCCAAA : 1548
MS44c2_nox : GAGATCAAAAATGTGAGCACCTGTGCCCCCAGCAATGTGTTTCTCTTGCTTATACACCCCACCTCTAGGCCTTGACGGAAGCCAAA : 1548
KH1a1_noxR : GAGATCAAAAATGTGAGCACCTGTGCCCCCAGCAATGTGTTTCTCTTGCTTATACACCCCACCTCTAGGCCTTGACGGAAGCCAAA : 1548
CBS1-1_nox : GAGATCAAAAATGTGAGCACCTGTGCCCCCAGCAATGTGTTTCTCTTGCTTATACACCCCACCTCTAGGCCTTGACGGAAGCCAAA : 1548
LF4f1_noxR : GAGATCAAAAATGTGAGCACCTGTGCCCCCAGCAATGTGTTTCTCTTGCTTATACACCCCACCTCTAGGCCTTGACGGAAGCCAAA : 1548
LF4e1_noxR : GAGATCAAAAATGTGAGCACCTGTGCCCCCAGCAATGTGTTTCTCTTGCTTATACACCCCACCTCTAGGCCTTGACGGAAGCCAAA : 1548
LF4d2_noxR : GAGATCAAAAATGTGAGCACCTGTGCCCCCAGCAATGTGTTTCTCTTGCTTATACACCCCACCTCTAGGCCTTGACGGAAGCCAAA : 1548
LF4a1_noxR : GAGATCAAAAATGTGAGCACCTGTGCCCCCAGCAATGTGTTTCTCTTGCTTATACACCCCACCTCTAGGCCTTGACGGAAGCCAAA : 1548
LF1e1_noxR : GAGATCAAAAATGTGAGCACCTGTGCCCCCAGCAATGTGTTTCTCTTGCTTATACACCCCACCTCTAGGCCTTGACGGAAGCCAAA : 1548
KH1d1_noxR : GAGATCAAAAATGTGAGCACCTGTGCCCCCAGCAATGTGTTTCTCTTGCTTATACACCCCACCTCTAGGCCTTGACGGAAGCCAAA : 1548
KH1c1_noxR : GAGATCAAAAATGTGAGCACCTGTGCCCCCAGCAATGTGTTTCTCTTGCTTATACACCCCACCTCTAGGCCTTGACGGAAGCCAAA : 1548
KH1h1_noxR : GAGATCAAAAATGTGAGCACCTGTGCCCCCAGCAATGTGTTTCTCTTGCTTATACACCCCACCTCTAGGCCTTGACGGAAGCCAAA : 1548
LF4a2_noxR : RAGATCAAAAATGTGAGCACCTGTGCCCCCAGCAATGTGTTTCTCTTGCTTATACACCCCACCTCTAGGCCTTGACGGAAGCCAAA : 1548
LF4h1_noxR : GAGATCAAAAATGTGAGCACCTGTGCCCCCAGCAATGTGTTTCTCTTGCTTATACACCCCACCTCTAGGCCTTGACGGAAGCCAAA : 1548
QM6a_cDNA  : GAGATCAAAAATG--------------------------------------------------------CCTTGACAGAAGCCAAA : 1208
QM6a_noxR  : GAGATCAAAAATGTGAGCACCTGTGTGCCCAGTAATGTATTTCTCTTACTTATCCAAACCCCCTCTAGGCCTTGACAGAAGCCAAA : 1544
consensus    GAGATCAAAAATGtgagcacctgtgcccccagcaatgtgtttctcttgcttatacaccccacctctaggCCTTGACgGAAGCCAAA

                 *       1560         *       1580         *       1600         *       1620         *
CBS1-2_nox : GACGACAGACCAAGCGAGAACCTGTCCTTCGCGGCGACAAACCTGGTCAAGCCTGGCCTTCAGTCACGACGACAGCAGTCCGAGCC : 1634
MS44c2_nox : GACGACAGACCAAGCGAGAACCTGTCCTTCGCGGCGACAAACCTGGTCAAGCCTGGCCTTCAGTCACGACGACAGCAGTCCGAGCC : 1634
KH1a1_noxR : GACGACAGACCAAGCGAGAACCTGTCCTTCGCGGCGACAAACCTGGTCAAGCCTGGCCTTCAGTCACGACGACAGCAGTCCGAGCC : 1634
CBS1-1_nox : GACGACAGACCAAGCGAGAACCTGTCCTTCGCGGCGACAAACCTGGTCAAGCCTGGCCTTCAGTCACGACGACAGCAGTCCGAGCC : 1634
LF4f1_noxR : GACGACAGACCAAGCGAGAACCTGTCCTTCGCGGCGACAAACCTGGTCAAGCCTGGCCTTCAGTCACGACGACAGCAGTCCGAGCC : 1634
LF4e1_noxR : GACGACAGACCAAGCGAGAACCTGTCCTTCGCGGCGACAAACCTGGTCAAGCCTGGCCTTCAGTCACGACGACAGCAGTCCGAGCC : 1634
LF4d2_noxR : GACGACAGACCAAGCGAGAACCTGTCCTTCGCGGCGACAAACCTGGTCAAGCCTGGCCTTCAGTCACGACGACAGCAGTCCGAGCC : 1634
LF4a1_noxR : GACGACAGACCAAGCGAGAACCTGTCCTTCGCGGCGACAAACCTGGTCAAGCCTGGCCTTCAGTCACGACGACAGCAGTCCGAGCC : 1634
LF1e1_noxR : GACGACAGACCAAGCGAGAACCTGTCCTTCGCGGCGACAAACCTGGTCAAGCCTGGCCTTCAGTCACGACGACAGCAGTCCGAGCC : 1634
KH1d1_noxR : GACGACAGACCAAGCGAGAACCTGTCCTTCGCGGCGACAAACCTGGTCAAGCCTGGCCTTCAGTCACGACGACAGCAGTCCGAGCC : 1634
KH1c1_noxR : GACGACAGACCAAGCGAGAACCTGTCCTTCGCGGCGACAAACCTGGTCAAGCCTGGCCTTCAGTCACGACGACAGCAGTCCGAGCC : 1634
KH1h1_noxR : GACGACAGACCAAGCGAGAACCTGTCCTTCGCGGCGACAAACCTGGTCAAGCCTGGCCTTCAGTCACGACGACAGCAGTCCGAGCC : 1634
LF4a2_noxR : GACGACAGACCAAGCGAGAACCTGTCCTTCGCGGCGACAAACCTGGTCAAGCCTGGCCTTCAGTCACGACGACAGCAGTCCGAGCC : 1634
LF4h1_noxR : GACGACAGACCAAGCGAGAACCTGTCCTTCGCGGCGACAAACCTGGTCAAGCCTGGCCTTCAGTCACGACGACAGCAGTCCGAGCC : 1634
QM6a_cDNA  : GACGACCGACCAAGCGAGAATCTGTCCTTCGCGGCGACAAACCTGGTCAAGCCTGGCCTTCAGTCACGACGACAGCAGTCCGAGCC : 1294
QM6a_noxR  : GACGACCGACCAAGCGAGAATCTGTCCTTCGCGGCGACAAACCTGGTCAAGCCTGGCCTTCAGTCACGACGACAGCAGTCCGAGCC : 1630
consensus    GACGACaGACCAAGCGAGAAcCTGTCCTTCGCGGCGACAAACCTGGTCAAGCCTGGCCTTCAGTCACGACGACAGCAGTCCGAGCC
```

```
                 1640         *      1660         *      1680         *      1700         *      1720
CBS1-2_nox : GCCGACCGATCGCAGCAACGCGTTCCCTCCCACTCCTCCTCCGGAGAACGACCGTCTCAACCGGGGTGCTTCCGAACGGAACTCTG : 1720
MS44c2_nox : GCCGACCGATCGCAGCAACGCGTTCCCTCCCACTCCTCCTCCGGAGAACGACCGTCTCAACCGGGGTGCTTCCGAACGGAACTCTG : 1720
KH1a1_noxR : GCCGACCGATCGCAGCAACGCGTTCCCTCCCACTCCTCCTCCGGAGAACGACCGTCTCAACCGGGGTGCTTCCGAACGGAACTCTG : 1720
CBS1-1_nox : GCCGACCGATCGCAGCAACGCGTTCCCTCCCACTCCTCCTCCGGAGAACGACCGTCTCAACCGGGGTGCTTCCGAACGGAACTCTG : 1720
LF4f1_noxR : GCCGACCGATCGCAGCAACGCGTTCCCTCCCACTCCTCCTCCGGAGAACGACCGTCTCAACCGGGGTGCTTCCGAACGGAACTCTG : 1720
LF4e1_noxR : GCCGACCGATCGCAGCAACGCGTTCCCTCCCACTCCTCCTCCGGAGAACGACCGTCTCAACCGGGGTGCTTCCGAACGGAACTCTG : 1720
LF4d2_noxR : GCCGACCGATCGCAGCAACGCGTTCCCTCCCACTCCTCCTCCGGAGAACGACCGTCTCAACCGGGGTGCTTCCGAACGGAACTCTG : 1720
LF4a1_noxR : GCCGACCGATCGCAGCAACGCGTTCCCTCCCACTCCTCCTCCGGAGAACGACCGTCTCAACCGGGGTGCTTCCGAACGGAACTCTG : 1720
LF1e1_noxR : GCCGACCGATCGCAGCAACGCGTTCCCTCCCACTCCTCCTCCGGAGAACGACCGTCTCAACCGGGGTGCTTCCGAACGGAACTCTG : 1720
KH1d1_noxR : GCCGACCGATCGCAGCAACGCGTTCCCTCCCACTCCTCCTCCGGAGAACGACCGTCTCAACCGGGGTGCTTCCGAACGGAACTCTG : 1720
KH1c1_noxR : GCCGACCGATCGCAGCAACGCGTTCCCTCCCACTCCTCCTCCGGAGAACGACCGTCTCAACCGGGGTGCTTCCGAACGGAACTCTG : 1720
KH1h1_noxR : GCCGACCGATCGCAGCAACGCGTTCCCTCCCACTCCTCCTCCGGAGAACGACCGTCTCAACCGGGGTGCTTCCGAACGGAACTCTG : 1720
LF4a2_noxR : GCCGACCGATCGCAGCAACGTGTTCCCTCCCACTCCTCCTGCGGAGAACGACCGTCTCAACCGGGGTGCTTCCGAACGGAACTCTG : 1720
LF4h1_noxR : GCCGACCGATCGCAGCAACGCGTTCCCTCCCACTCCTCCTCCGGAGAACGACCGTCTCAACCGGGGTGCTTCCGAACGGAACTCTG : 1720
QM6a_cDNA  : GCCGACCGATCGCAGCAACGCGTTCCCTCCCACTCCTCCTCCGGAGAACGACCGTCTCAACCGGGGTGCTTCCGAACGGAACTCTG : 1380
QM6a_noxR  : GCCGACCGATCGCAGCAACGCGTTCCCTCCCACTCCTCCTCCGGAGAACGACCGTCTCAACCGGGGTGCTTCCGAACGGAACTCTG : 1716
consensus    GCCGACCGATCGCAGCAACGcGTTCCCTCCCACTCCTCCTcCGGAGAACGACCGTCTCAACCGGGGTGCTTCCGAACGGAACTCTG

                         *      1740         *      1760         *      1780         *      1800
CBS1-2_nox : GAGGGAGAGGGAAACTTAGCATCCAGACGCAAGAGCCCAGCCGGAGATATGAGAAGGCAATTTCGCCACAAGAACGCTCTAGGCAC : 1806
MS44c2_nox : GAGGGAGAGGGAAACTTAGCATCCAGACGCAAGAGCCCAGCCGGAGATATGAGAAGGCAATTTCGCCACAAGAACGCTCTAGGCAC : 1806
KH1a1_noxR : GAGGGAGAGGGAAACTTAGCATCCAGACGCAAGAGCCCAGCCGGAGATATGAGAAGGCAATTTCGCCACAAGAACGCTCTAGGCAC : 1806
CBS1-1_nox : GAGGGAGAGGGAAACTTAGCATCCAGACGCAAGAGCCCAGCCGGAGATATGAGAAGGCAATTTCGCCACAAGAACGCTCTAGGCAC : 1806
LF4f1_noxR : GAGGGAGAGGGAAACTTAGCATCCAGACGCAAGAGCCCAGCCGGAGATATGAGAAGGCAATTTCGCCACAAGAACGCTCTAGGCAC : 1806
LF4e1_noxR : GAGGGAGAGGGAAACTTAGCATCCAGACGCAAGAGCCCAGCCGGAGATATGAGAAGGCAATTTCGCCACAAGAACGCTCTAGGCAC : 1806
LF4d2_noxR : GAGGGAGAGGGAAACTTAGCATCCAGACGCAAGAGCCCAGCCGGAGATATGAGAAGGCAATTTCGCCACAAGAACGCTCTAGGCAC : 1806
LF4a1_noxR : GAGGGAGAGGGAAACTTAGCATCCAGACGCAAGAGCCCAGCCGGAGATATGAGAAGGCAATTTCGCCACAAGAACGCTCTAGGCAC : 1806
LF1e1_noxR : GAGGGAGAGGGAAACTTAGCATCCAGACGCAAGAGCCCAGCCGGAGATATGAGAAGGCAATTTCGCCACAAGAACGCTCTAGGCAC : 1806
KH1d1_noxR : GAGGGAGAGGGAAACTTAGCATCCAGACGCAAGAGCCCAGCCGGAGATATGAGAAGGCAATTTCGCCACAAGAACGCTCTAGGCAC : 1806
KH1c1_noxR : GAGGGAGAGGGAAACTTAGCATCCAGACGCAAGAGCCCAGCCGGAGATATGAGAAGGCAATTTCGCCACAAGAACGCTCTAGGCAC : 1806
KH1h1_noxR : GAGGGAGAGGGAAACTTAGCATCCAGACGCAAGAGCCCAGCCGGAGATATGAGAAGGCAATTTCGCCACAAGAACGCTCTAGGCAC : 1806
LF4a2_noxR : GAGGGAGAGGGAAACTTAGCATCCAGACGCAAGAGCCCAGCCGGAGATATGAGAAGGCAATTTCGCCACAAGAACGCTCTAGGCAC : 1806
LF4h1_noxR : GAGGGAGAGGGAAACTTAGCATCCAGACGCAAGAGCCCAGCCGGAGATATGAGAAGGCAATTTCGCCACAAGAACGCTCTAGGCAC : 1806
QM6a_cDNA  : GAGGGAGAGGGAAACTTAGCATCCAAACGCAAGAGCCCAGCCGGAGATATGAGAAGGCAATCTCGCCACAAGAACGCTCTAGGCAC : 1466
QM6a_noxR  : GAGGGAGAGGGAAACTTAGCATCCAAACGCAAGAGCCCAGCCGGAGATATGAGAAGGCAATCTCGCCACAAGAACGCTCTAGGCAC : 1802
consensus    GAGGGAGAGGGAAACTTAGCATCCAgACGCAAGAGCCCAGCCGGAGATATGAGAAGGCAATtTCGCCACAAGAACGCTCTAGGCAC

                  *      1820         *      1840         *      1860         *      1880         *
CBS1-2_nox : GGGCGATCGGCATCAGCTGCCCCTGCGAGAGGCCTTTCGACGAGGGATCCCCAGCGGAGACAGAGGCCTATCGAGGAGGACTATCC : 1892
MS44c2_nox : GGGCGATCGGCATCAGCTGCCCCTGCGAGAGGCCTTTCGACGAGGGATCCCCAGCGGAGACAGAGGCCTATCGAGGAGGACTATCC : 1892
KH1a1_noxR : GGGCGATCGGCATCAGCTGCCCCTGCGAGAGGCCTTTCGACGAGGGATCCCCAGCGGAGACAGAGGCCTATCGAGGAGGACTATCC : 1892
CBS1-1_nox : GGGCGATCGGCATCAGCTGCCCCTGCGAGAGGCCTTTCGACGAGGGATCCCCAGCGGAGACAGAGGCCTATCGAGGAGGACTATCC : 1892
LF4f1_noxR : GGGCGATCGGCATCAGCTGCCCCTGCGAGAGGCCTTTCGACGAGGGATCCCCAGCGGAGACAGAGGCCTATCGAGGAGGACTATCC : 1892
LF4e1_noxR : GGGCGATCGGCATCAGCTGCCCCTGCGAGAGGCCTTTCGACGAGGGATCCCCAGCGGAGACAGAGGCCTATCGAGGAGGACTATCC : 1892
LF4d2_noxR : GGGCGATCGGCATCAGCTGCCCCTGCGAGAGGCCTTTCGACGAGGGATCCCCAGCGGAGACAGAGGCCTATCGAGGAGGACTATCC : 1892
LF4a1_noxR : GGGCGATCGGCATCAGCTGCCCCTGCGAGAGGCCTTTCGACGAGGGATCCCCAGCGGAGACAGAGGCCTATCGAGGAGGACTATCC : 1892
LF1e1_noxR : GGGCGATCGGCATCAGCTGCCCCTGCGAGAGGCCTTTCGACGAGGGATCCCCAGCGGAGACAGAGGCCTATCGAGGAGGACTATCC : 1892
KH1d1_noxR : GGGCGATCGGCATCAGCTGCCCCTGCGAGAGGCCTTTCGACGAGGGATCCCCAGCGGAGACAGAGGCCTATCGAGGAGGACTATCC : 1892
KH1c1_noxR : GGGCGATCGGCATCAGCTGCCCCTGCGAGAGGCCTTTCGACGAGGGATCCCCAGCGGAGACAGAGGCCTATCGAGGAGGACTATCC : 1892
KH1h1_noxR : GGGCGATCGGCATCAGCTGCCCCTGCGAGAGGCCTTTCGACGAGGGATCCCCAGCGGAGACAGAGGCCTATCGAGGAGGACTATCC : 1892
LF4a2_noxR : GGGCGATCGGCATCAGCTGCCCCTGCGAGAGGCCTTTCGACGAGGGATCCCCAGCGGAGACAGAGGCCTATCGAGGAGGACTATCC : 1892
LF4h1_noxR : GGGCGATCGGCATCAGCTGCCCCTGCGAGAGGCCTTTCGACGAGGGATCCCCAGCGGAGACAGAGGCCTATCGAGGAGGACTATCC : 1892
QM6a_cDNA  : GGGCGATCGGCATCAGCTGCCCCTGCGAGAGGCCTTTCGACGAGGGATCCCCAGCGGAGACAGAGGCCTATCGAGGAGGACTATCC : 1552
QM6a_noxR  : GGGCGATCGGCATCAGCTGCCCCTGCGAGAGGCCTTTCGACGAGGGATCCCCAGCGGAGACAGAGGCCTATCGAGGAGGACTATCC : 1888
consensus    GGGCGATCGGCATCAGCTGCCCCTGCGAGAGGCCTTTCGACGAGGGATCCCCAGCGGAGACAGAGGCCTATCGAGGAGGACTATCC

                   1900         *      1920         *      1940         *      1960         *      19
CBS1-2_nox : CTACGGCGACGAGCAATACGACCCGTACGCCGTTGGACAGCGAGGAAGCAAGGGGTCTCGATCGCGACAACAGCGATACGACGACC : 1978
MS44c2_nox : CTACGGCGACGAGCAATACGACCCGTACGCCGTTGGACAGCGAGGAAGCAAGGGGTCTCGATCGCGACAACAGCGATACGACGACC : 1978
KH1a1_noxR : CTACGGCGACGAGCAATACGACCCGTACGCCGTTGGACAGCGAGGAAGCAAGGGGTCTCGATCGCGACAACAGCGATACGACGACC : 1978
CBS1-1_nox : CTACGGCGACGAGCAATACGACCCGTACGCCGTTGGACAGCGAGGAAGCAAGGGGTCTCGATCGCGACAACAGCGATACGACGACC : 1978
LF4f1_noxR : CTACGGCGACGAGCAATACGACCCGTACGCCGTTGGACAGCGAGGAAGCAAGGGGTCTCGATCGCGACAACAGCGATACGACGACC : 1978
LF4e1_noxR : CTACGGCGACGAGCAATACGACCCGTACGCCGTTGGACAGCGAGGAAGCAAGGGGTCTCGATCGCGACAACAGCGATACGACGACC : 1978
LF4d2_noxR : CTACGGCGACGAGCAATACGACCCGTACGCCGTTGGACAGCGAGGAAGCAAGGGGTCTCGATCGCGACAACAGCGATACGACGACC : 1978
LF4a1_noxR : CTACGGCGACGAGCAATACGACCCGTACGCCGTTGGACAGCGAGGAAGCAAGGGGTCTCGATCGCGACAACAGCGATACGACGACC : 1978
LF1e1_noxR : CTACGGCGACGAGCAATACGACCCGTACGCCGTTGGACAGCGAGGAAGCAAGGGGTCTCGATCGCGACAACAGCGATACGACGACC : 1978
KH1d1_noxR : CTACGGCGACGAGCAATACGACCCGTACGCCGTTGGACAGCGAGGAAGCAAGGGGTCTCGATCGCGACAACAGCGATACGACGACC : 1978
KH1c1_noxR : CTACGGCGACGAGCAATACGACCCGTACGCCGTTGGACAGCGAGGAAGCAAGGGGTCTCGATCGCGACAACAGCGATACGACGACC : 1978
KH1h1_noxR : CTACGGCGACGAGCAATACGACCCGTACGCCGTTGGACAGCGAGGAAGCAAGGGGTCTCGATCGCGACAACAGCGATACGACGACC : 1978
LF4a2_noxR : CTACGGCGACGAGCAATACGACCCGTACGCCGTTGGACAGCGAGGAAGCAAGGGGTCTCGATCGCGACAACAGCGATACGACGACC : 1978
LF4h1_noxR : CTACGGCGACGAGCAATACGACCCGTACGCCGTTGGACAGCGAGGAAGCAAGGGGTCTCGATCGCGACAACAGCGATACGACGACC : 1978
QM6a_cDNA  : CTACGGCGACGAGCAATACGACCCGTACGCCGTTGGACAGCGAGGAAGCAAGGGGTCTCGATCGCGACAACAGCGATACGATGACC : 1638
```

```
QM6a_noxR  : CTACGGCGACGAGCAATACGACCCGTACGCCGTTGGACAGCGAGGAAGCAAGGGGTCTCGATCGCGACAACAGCGATACGATGACC : 1974
consensus    CTACGGCGACGAGCAATACGACCCGTACGCCGTTGGACAGCGAGGAAGCAAGGGGTCTCGATCGCGACAACAGCGATACGAcGACC


              80         *       2000         *       2020         *       2040         *       2060
CBS1-2_nox : GGTACGACGACCGATATGAGGATGAAGAGGACTCGGAAAATGACTATGGCTCCTTTGACGAGGGCGAGTTCGAGATGGTGTCCACG : 2064
MS44c2_nox : GGTACGACGACCGATATGAGGATGAAGAGGACTCGGAAAATGACTATGGCTCCTTTGACGAGGGCGAGTTCGAGATGGTGTCCACG : 2064
KH1a1_noxR : GGTACGACGACCGATATGAGGATGAAGAGGACTCGGAAAATGACTATGGCTCCTTTGACGAGGGCGAGTTCGAGATGGTGTCCACG : 2064
CBS1-1_nox : GGTACGACGACCGATATGAGGATGAAGAGGACTCGGAAAATGACTATGGCTCCTTTGACGAGGGCGAGTTCGAGATGGTGTCCACG : 2064
LF4f1_noxR : GGTACGACGACCGATATGAGGATGAAGAGGACTCGGAAAATGACTATGGCTCCTTTGACGAGGGCGAGTTCGAGATGGTGTCCACG : 2064
LF4e1_noxR : GGTACGACGACCGATATGAGGATGAAGAGGACTCGGAAAATGACTATGGCTCCTTTGACGAGGGCGAGTTCGAGATGGTGTCCACG : 2064
LF4d2_noxR : GGTACGACGACCGATATGAGGATGAAGAGGACTCGGAAAATGACTATGGCTCCTTTGACGAGGGCGAGTTCGAGATGGTGTCCACG : 2064
LF4a1_noxR : GGTACGACGACCGATATGAGGATGAAGAGGACTCGGAAAATGACTATGGCTCCTTTGACGAGGGCGAGTTCGAGATGGTGTCCACG : 2064
LF1e1_noxR : GGTACGACGACCGATATGAGGATGAAGAGGACTCGGAAAATGACTATGGCTCCTTTGACGAGGGCGAGTTCGAGATGGTGTCCACG : 2064
KH1d1_noxR : GGTACGACGACCGATATGAGGATGAAGAGGACTCGGAAAATGACTATGGCTCCTTTGACGAGGGCGAGTTCGAGATGGTGTCCACG : 2064
KH1c1_noxR : GGTACGACGACCGATATGAGGATGAAGAGGACTCGGAAAATGACTATGGCTCCTTTGACGAGGGCGAGTTCGAGATGGTGTCCACG : 2064
KH1h1_noxR : GGTACGACGACCGATATGAGGATGAAGAGGACTCGGAAAATGACTATGGCTCCTTTGACGAGGGCGAGTTCGAGATGGTGTCCACG : 2064
LF4a2_noxR : GGTACGACGACCGATATGAGGATGAAGAGGACTCGGAAAATGACTATGGCTCCTTTGACGAGGGCGAGTTCGAGATGGTGTCCACG : 2064
LF4h1_noxR : GGTACGACGACCGATATGAGGATGAAGAGGACTCGGAAAATGACTATGGCTCCTTTGACGAGGGCGAGTTCGAGATGGTGTCCACG : 2064
QM6a_cDNA  : GGTACGACGACCGATATGAGGATGAAGAGGACTCGGAAAATGACTACGGCTCCTTTGACGAGGGCGAGTTCGAGATGGTGTCCACG : 1724
QM6a_noxR  : GGTACGACGACCGATATGAGGATGAAGAGGACTCGGAAAATGACTACGGCTCCTTTGACGAGGGCGAGTTCGAGATGGTGTCCACG : 2060
consensus    GGTACGACGACCGATATGAGGATGAAGAGGACTCGGAAAATGACTAtGGCTCCTTTGACGAGGGCGAGTTCGAGATGGTGTCCACG


                   *       2080         *       2100         *       2120         *       2140         *
CBS1-2_nox : ATGCGACGCGGCCCCGGGTCGGTGGCGCGGCCATCGCGTGCGGCGTCGCGACGACCGCCCGAGATTCGCAAGATCCGCGTCAAGGT : 2150
MS44c2_nox : ATGCGACGCGGCCCCGGGTCGGTGGCGCGGCCATCGCGTGCGGCGTCGCGACGACCGCCCGAGATTCGCAAGATCCGCGTCAAGGT : 2150
KH1a1_noxR : ATGCGACGCGGCCCCGGGTCGGTGGCGCGGCCATCGCGTGCGGCGTCGCGACGACCGCCCGAGATTCGCAAGATCCGCGTCAAGGT : 2150
CBS1-1_nox : ATGCGACGCGGCCCCGGGTCGGTGGCGCGGCCATCGCGTGCGGCGTCGCGACGACCGCCCGAGATTCGCAAGATCCGCGTCAAGGT : 2150
LF4f1_noxR : ATGCGACGCGGCCCCGGGTCGGTGGCGCGGCCATCGCGTGCGGCGTCGCGACGACCGCCCGAGATTCGCAAGATCCGCGTCAAGGT : 2150
LF4e1_noxR : ATGCGACGCGGCCCCGGGTCGGTGGCGCGGCCATCGCGTGCGGCGTCGCGACGACCGCCCGAGATTCGCAAGATCCGCGTCAAGGT : 2150
LF4d2_noxR : ATGCGACGCGGCCCCGGGTCGGTGGCGCGGCCATCGCGTGCGGCGTCGCGACGACCGCCCGAGATTCGCAAGATCCGCGTCAAGGT : 2150
LF4a1_noxR : ATGCGACGCGGCCCCGGGTCGGTGGCGCGGCCATCGCGTGCGGCGTCGCGACGACCGCCCGAGATTCGCAAGATCCGCGTCAAGGT : 2150
LF1e1_noxR : ATGCGACGCGGCCCCGGGTCGGTGGCGCGGCCATCGCGTGCGGCGTCGCGACGACCGCCCGAGATTCGCAAGATCCGCGTCAAGGT : 2150
KH1d1_noxR : ATGCGACGCGGCCCCGGGTCGGTGGCGCGGCCATCGCGTGCGGCGTCGCGACGACCGCCCGAGATTCGCAAGATCCGCGTCAAGGT : 2150
KH1c1_noxR : ATGCGACGCGGCCCCGGGTCGGTGGCGCGGCCATCGCGTGCGGCGTCGCGACGACCGCCCGAGATTCGCAAGATCCGCGTCAAGGT : 2150
KH1h1_noxR : ATGCGACGCGGCCCCGGGTCGGTGGCGCGGCCATCGCGTGCGGCGTCGCGACGACCGCCCGAGATTCGCAAGATCCGCGTCAAGGT : 2150
LF4a2_noxR : ATGCGACGCGGCCCCGGGTCGGTGGCGCGGCCATCGCGTGCGGCGTCGCGACGACCGCCCGAGATTCGCAAGATCCGCGTCAAGGT : 2150
LF4h1_noxR : ATGCGACGCGGCCCCGGGTCGGTGGCGCGGCCATCGCGTGCGGCGTCGCGACGACCGCCCGAGATTCGCAAGATCCGCGTCAAGGT : 2150
QM6a_cDNA  : ATGCGACGCGGCCCCGGGTCGGTGGCGCGGCCCTCGCGTGCGGCGTCGCGACGACCGCCGGAGATTCGCAAGATCCGCGTCAAGGT : 1810
QM6a_noxR  : ATGCGACGCGGCCCCGGGTCGGTGGCGCGGCCCTCGCGTGCGGCGTCGCGACGACCGCCGGAGATTCGCAAGATCCGCGTCAAGGT : 2146
consensus    ATGCGACGCGGCCCCGGGTCGGTGGCGCGGCCaTCGCGTGCGGCGTCGCGACGACCGCCcGAGATTCGCAAGATCCGCGTCAAGGT


                   2160         *       2180         *       2200         *       2220
CBS1-2_nox : GCACTCAGACGATGTGAGATACATCATGATTGGCACGACGATTGAGTTCTCCGACTTTGTGGATCGCATCCGAGACAA : 2228
MS44c2_nox : GCACTCAGACGATGTGAGATACATCATGATTGGCACGACGATTGAGTTCTCCGACTTTGTGGATCGCATCCGAGACAA : 2228
KH1a1_noxR : GCACTCAGACGATGTGAGATACATCATGATTGGCACGACGATTGAGTTCTCCGACTTTGTGGATCGCATCCGAGACAA : 2228
CBS1-1_nox : GCACTCAGACGATGTGAGATACATCATGATTGGCACGACGATTGAGTTCTCCGACTTTGTGGATCGCATCCGAGACAA : 2228
LF4f1_noxR : GCACTCAGACGATGTGAGATACATCATGATTGGCACGACGATTGAGTTCTCCGACTTTGTGGATCGCATCCGAGACAA : 2228
LF4e1_noxR : GCACTCAGACGATGTGAGATACATCATGATTGGCACGACGATTGAGTTCTCCGACTTTGTGGATCGCATCCGAGACAA : 2228
LF4d2_noxR : GCACTCAGACGATGTGAGATACATCATGATTGGCACGACGATTGAGTTCTCCGACTTTGTGGATCGCATCCGAGACAA : 2228
LF4a1_noxR : GCACTCAGACGATGTGAGATACATCATGATTGGCACGACGATTGAGTTCTCCGACTTTGTGGATCGCATCCGAGACAA : 2228
LF1e1_noxR : GCACTCAGACGATGTGAGATACATCATGATTGGCACGACGATTGAGTTCTCCGACTTTGTGGATCGCATCCGAGACAA : 2228
KH1d1_noxR : GCACTCAGACGATGTGAGATACATCATGATTGGCACGACGATTGAGTTCTCCGACTTTGTGGATCGCATCCGAGACAA : 2228
KH1c1_noxR : GCACTCAGACGATGTGAGATACATCATGATTGGCACGACGATTGAGTTCTCCGACTTTGTGGATCGCATCCGAGACAA : 2228
KH1h1_noxR : GCACTCAGACGATGTGAGATACATCATGATTGGCACGACGATTGAGTTCTCCGACTTTGTGGATCGCATCCGAGACAA : 2228
LF4a2_noxR : GCACTCAGACGATGTGAGATACATCATGATTGGCACGACGATTGAGTTCTCCGACTTTGTGGATCGCATCCGAGACAA : 2228
LF4h1_noxR : GCACTCAGACGATGTGAGATACATCATGATTGGCACGACGATTGAGTTCTCCGACTTTGTGGATCGCATCCGAGACAA : 2228
QM6a_cDNA  : GCACTCAGACGATGTGCGATACATCATGATTGGCACGACGATTGAGTTTTCCGACTTTGTGGATCGTATCCGAGACAA : 1888
QM6a_noxR  : GCACTCAGACGATGTGCGATACATCATGATTGGCACGACGATTGAGTTTTCCGACTTTGTGGATCGTATCCGAGACAA : 2224
consensus    GCACTCAGACGATGTGaGATACATCATGATTGGCACGACGATTGAGTTcTCCGACTTTGTGGATCGcATCCGAGACAA
```

Sequence alignment *tmk1*

```
                        *        20         *        40         *        60         *
QM6a        : ATGTCTCGGTCGAACCCCCCCAATAACGCCTCGGCGTCGCGCAAGATCTCCTTCAACGTCAGCGAGCAGTAT :   72
RUTC30      : ATGTCTCGGTCGAACCCCCCCAATAACGCCTCGGCGTCGCGCAAGATCTCCTTCAACGTCAGCGAGCAGTAT :   72
AIT_TRLF1e  : ATGTCTCGGTCGAACCCCCCCAATAACGCCTCGGCGTCGCGCAAGATCTCCTTCAACGTCAGCGAGCAGTAT :   72
AIT_TRKH1a  : ATGTCTCGGTCGAACCCCCCCAATAACGCCTCGGCGTCGCGCAAGATCTCCTTCAACGTCAGCGAGCAGTAT :   72
CBS999.97   : ATGTCTCGGTCGAACCCCCCCAATAACGCCTCGGCGTCGCGCAAGATCTCCTTCAACGTCAGCGAGCAGTAT :   72
AIT_TRKH1c  : ATGTCTCGGTCGAACCCCCCCAATAACGCCTCGGCGTCGCGCAAGATCTCCTTCAACGTCAGCGAGCAGTAT :   72
AIT_TRMS44  : ATGTCTCGGTCGAACCCCCCCAATAACGCCTCGGCGTCGCGCAAGATCTCCTTCAACGTCAGCGAGCAGTAT :   72
               ATGTCTCGGTCGAACCCCCCCAATAACGCCTCGGCGTCGCGCAAGATCTCCTTCAACGTCAGCGAGCAGTAT

                     80         *       100         *       120         *       140
QM6a        : GACATTCAGGATGTTGTTGGCGAAGGAGCCTACGGAGTTGTCTGGTAAGTGTCTGGTGTGCTACCTCTGAAG :  144
RUTC30      : GACATTCAGGATGTTGTTGGCGAAGGAGCCTACGGAGTTGTCTGGTAAGTGTCTGGTGTGCTACCTCTGAAG :  144
AIT_TRLF1e  : GACATTCAGGATGTTGTTGGCGAAGGAGCCTACGGAGTTGTCTGGTAAGTGTCTGGTGTGCTACCTCTGAAG :  144
AIT_TRKH1a  : GACATTCAGGATGTTGTTGGCGAAGGAGCCTACGGAGTTGTCTGGTAAGTGTCTGGTGTGCTACCTCTGAAG :  144
CBS999.97   : GACATCCAGGATGTTGTTGGCGAAGGAGCCTACGGAGTTGTCTGGTAAGTGTCTGGTGTGCTACCTCTGAAG :  144
AIT_TRKH1c  : GACATCCAGGATGTTGTTGGCGAAGGAGCCTACGGAGTTGTCTGGTAAGTGTCTGGTGTGCTACCTCTGAAG :  144
AIT_TRMS44  : GACATCCAGGATGTTGTTGGCGAAGGAGCCTACGGAGTTGTCTGGTAAGTGTCTGGTGTGCTACCTCTGAAG :  144
               GACAT CAGGATGTTGTTGGCGAAGGAGCCTACGGAGTTGTCTGGTAAGTGTCTGGTGTGCTACCTCTGAAG

                    *       160         *       180         *       200         *
QM6a        : AGAGGCAGAGACCCAGGGGGTGGTGCACACGCCGTATCTAACAGCTTTTCCTACGACAAACAGCTCCGCTAT :  216
RUTC30      : AGAGGCAGAGACCCAGGGGGTGGTGCACACGCCGTATCTAACAGCTTTTCCTACGACAAACAGCTCCGCTAT :  216
AIT_TRLF1e  : AGAGGCAGAGACCCAGGGGGTGGTGCACACGCCGTATCTAACAGCTTTTCCTACGACAAACAGCTCCGCTAT :  216
AIT_TRKH1a  : AGAGGCAGAGACCCAGGGGGTGGTGCACACGCCGTATCTAACAGCTTTTCCTACGACAAACAGCTCCGCTAT :  216
CBS999.97   : AGAGGCAGAGACCCAGGGGATGGTGCACACGCCGTATCTAACAGCTTTTCCTACGACAAACAGCTCCGCTAT :  216
AIT_TRKH1c  : AGAGGCAGAGACCCAGGGGATGGTGCACACGCCGTATCTAACAGCTTTTCCTACGACAAACAGCTCCGCTAT :  216
AIT_TRMS44  : AGAGGCAGAGACCCAGGGGATGGTGCACACGCCGTATCTAACAGCTTTTCCTACGACAAACAGCTCCGCTAT :  216
               AGAGGCAGAGACCCAGGGG TGGTGCACACGCCGTATCTAACAGCTTTTCCTACGACAAACAGCTCCGCTAT

                220         *       240         *       260         *       280
QM6a        : CCACAAGCCCTCGGGACAAAAGGTTGCCATCAAGAAGATAACTCCCTTCGACCACTCCATGTTCTGCCTGAG :  288
RUTC30      : CCACAAGCCCTCGGGACAAAAGGTTGCCATCAAGAAGATAACTCCCTTCGACCACTCCATGTTCTGCCTGAG :  288
AIT_TRLF1e  : CCACAAGCCCTCGGGACAAAAGGTTGCCATCAAGAAGATAACTCCCTTCGACCACTCCATGTTCTGCCTGAG :  288
AIT_TRKH1a  : CCACAAGCCCTCGGGACAAAAGGTTGCCATCAAGAAGATAACTCCCTTCGACCACTCCATGTTCTGCCTGAG :  288
CBS999.97   : CCACAAGCCCTCGGGACAAAAGGTTGCCATCAAGAAGATTACTCCCTTCGACCACTCCATGTTCTGCCTGAG :  288
AIT_TRKH1c  : CCACAAGCCCTCGGGACAAAAGGTTGCCATCAAGAAGATTACTCCCTTCGACCACTCCATGTTCTGCCTGAG :  288
AIT_TRMS44  : CCACAAGCCCTCGGGACAAAAGGTTGCCATCAAGAAGATTACTCCCTTCGACCACTCCATGTTCTGCCTGAG :  288
               CCACAAGCCCTCGGGACAAAAGGTTGCCATCAAGAAGAT ACTCCCTTCGACCACTCCATGTTCTGCCTGAG

                *       300         *       320         *       340         *       360
QM6a        : AACCCTGCGAGAGATGAAGCTGCTGCGCTACTTCAACCACGAGAACATCATCTCCATCCTTGACATCCAGAA :  360
RUTC30      : AACCCTGCGAGAGATGAAGCTGCTGCGCTACTTCAACCACGAGAACATCATCTCCATCCTTGACATCCAGAA :  360
AIT_TRLF1e  : AACCCTGCGAGAGATGAAGCTGCTGCGCTACTTCAACCACGAGAACATCATCTCCATCCTTGACATCCAGAA :  360
AIT_TRKH1a  : AACCCTGCGAGAGATGAAGCTGCTGCGCTACTTCAACCACGAGAACATCATCTCCATCCTTGACATCCAGAA :  360
CBS999.97   : AACCCTGCGAGAGATGAAGCTGCTGCGCTACTTCAACCACGAGAACATCATCTCCATCCTTGACATCCAGAA :  360
AIT_TRKH1c  : AACCCTGCGAGAGATGAAGCTGCTGCGCTACTTCAACCACGAGAACATCATCTCCATCCTTGACATCCAGAA :  360
AIT_TRMS44  : AACCCTGCGAGAGATGAAGCTGCTGCGCTACTTCAACCACGAGAACATCATCTCCATCCTTGACATCCAGAA :  360
               AACCCTGCGAGAGATGAAGCTGCTGCGCTACTTCAACCACGAGAACATCATCTCCATCCTTGACATCCAGAA

                        *       380         *       400         *       420         *
QM6a        : GCCCCGGAGCTATGAGAGCTTTAACGAGGTCTATCTGATCCAGGTACGTCCCCAGCTGCTGTTTCGCAGGTC :  432
RUTC30      : GCCCCGGAGCTATGAGAGCTTTAACGAGGTCTATCTGATCCAGGTACGTCCCCAGCTGCTGTTTCGCAGGTC :  432
AIT_TRLF1e  : GCCCCGGAGCTATGAGAGCTTTAACGAGGTCTATCTGATCCAGGTACGTCCCCAGCTGCTGTTTCGCAGGTC :  432
AIT_TRKH1a  : GCCCCGGAGCTATGAGAGCTTTAACGAGGTCTATCTGATCCAGGTACGTCCCCAGCTGCTGTTTCGCAGGTC :  432
CBS999.97   : GCCCCGGAGCTATGAGAGCTTTAACGAGGTCTATCTGATCCAGGTACGTCCCCAGCTGCTGTTTCGCAGGTC :  432
AIT_TRKH1c  : GCCCCGGAGCTATGAGAGCTTTAACGAGGTCTATCTGATCCAGGTACGTCCCCAGCTGCTGTTTCGCAGGTC :  432
AIT_TRMS44  : GCCCCGGAGCTATGAGAGCTTTAACGAGGTCTATCTGATCCAGGTACGTCCCCAGCTGCTGTTTCGCAGGTC :  432
               GCCCCGGAGCTATGAGAGCTTTAACGAGGTCTATCTGATCCAGGTACGTCCCCAGCTGCTGTTTCGCAGGTC

                    440         *       460         *       480         *       500
QM6a        : CCAGTCAGCTCTGCCTACCTAGTATTGGCATTGCCTTGCCTAGATTGTGTGCTCACCACATCTTGCTTGTAG :  504
RUTC30      : CCAGTCAGCTCTGCCTACCTAGTATTGGCATTGCCTTGCCTAGATTGTGTGCTCACCACATCTTGCTTGTAG :  504
AIT_TRLF1e  : CCAGTCAGCTCTGCCTACCTAGTATTGGCATTGCCTTGCCTAGATTGTGTGCTCACCACATCTTGCTTGTAG :  504
AIT_TRKH1a  : CCAGTCAGCTCTGCCTACCTAGTATTGGCATTGCCTTGCCTAGATTGTGTGCTCACCACATCTTGCTTGTAG :  504
CBS999.97   : CCAGTCAGCTCTGCCTACCTAGTATTGGCCTTGCCTTGCCTAGATTGTGTGCTCACCACATCTTGCTTGTAG :  504
AIT_TRKH1c  : CCAGTCAGCTCTGCCTACCTAGTATTGGCCTTGCCTTGCCTAGATTGTGTGCTCACCACATCTTGCTTGTAG :  504
AIT_TRMS44  : CCAGTCAGCTCTGCCTACCTAGTATTGGCCTTGCCTTGCCTAGATTGTGTGCTCACCACATCTTGCTTGTAG :  504
               CCAGTCAGCTCTGCCTACCTAGTATTGGC TTGCCTTGCCTAGATTGTGTGCTCACCACATCTTGCTTGTAG

                    *       520         *       540         *       560         *
QM6a        : GAGCTCATGGAGACGGACATGCACAGAGTCATCCGAACCCAGGACCTGTCCGACGATCACTGCCAATACTTC :  576
RUTC30      : GAGCTCATGGAGACGGACATGCACAGAGTCATCCGAACCCAGGACCTGTCCGACGATCACTGCCAATACTTC :  576
AIT_TRLF1e  : GAGCTCATGGAGACGGACATGCACAGAGTCATCCGAACCCAGGACCTGTCCGACGATCACTGCCAATACTTC :  576
AIT_TRKH1a  : GAGCTCATGGAGACGGACATGCACAGAGTCATCCGAACCCAGGACCTGTCCGACGATCACTGCCAATACTTC :  576
```

```
CBS999.97  : GAGCTCATGGAGACGGACATGCACAGAGTCATCCGAACCCAGGACCTGTCCGACGATCACTGCCAATACTTC :  576
AIT_TRKH1c : GAGCTCATGGAGACGGACATGCACAGAGTCATCCGAACCCAGGACCTGTCCGACGATCACTGCCAATACTTC :  576
AIT_TRMS44 : GAGCTCATGGAGACGGACATGCACAGAGTCATCCGAACCCAGGACCTGTCCGACGATCACTGCCAATACTTC :  576
             GAGCTCATGGAGACGGACATGCACAGAGTCATCCGAACCCAGGACCTGTCCGACGATCACTGCCAATACTTC

                580         *       600         *       620         *       640
QM6a       : ATCTACCAGACGCTGCGAGCCCTCAAGGCCATGCACTCGGCAAACGTGCTGCACCGAGATCTCAAGCCGTCC :  648
RUTC30     : ATCTACCAGACGCTGCGAGCCCTCAAGGCCATGCACTCGGCAAACGTGCTGCACCGAGATCTCAAGCCGTCC :  648
AIT_TRLF1e : ATCTACCAGACGCTGCGAGCCCTCAAGGCCATGCACTCGGCAAACGTGCTGCACCGAGATCTCAAGCCGTCC :  648
AIT_TRKH1a : ATCTACCAGACGCTGCGAGCCCTCAAGGCCATGCACTCGGCAAACGTGCTGCACCGAGATCTCAAGCCGTCC :  648
CBS999.97  : ATCTACCAGACGCTGCGAGCCCTCAAGGCCATGCACTCGGCAAACGTGCTGCACCGAGATCTCAAGCCGTCC :  648
AIT_TRKH1c : ATCTACCAGACGCTGCGAGCCCTCAAGGCCATGCACTCGGCAAACGTGCTGCACCGAGATCTCAAGCCGTCC :  648
AIT_TRMS44 : ATCTACCAGACGCTGCGAGCCCTCAAGGCCATGCACTCGGCAAACGTGCTGCACCGAGATCTCAAGCCGTCC :  648
             ATCTACCAGACGCTGCGAGCCCTCAAGGCCATGCACTCGGCAAACGTGCTGCACCGAGATCTCAAGCCGTCC

                *       660         *       680         *       700         *       720
QM6a       : AACCTGTTGCTCAACGCCAACTGCGACCTCAAGGTCTGCGACTTCGGTCTGGCCCGGTCCGCCGCCTCCCAG :  720
RUTC30     : AACCTGTTGCTCAACGCCAACTGCGACCTCAAGGTCTGCGACTTCGGTCTGGCCCGGTCCGCCGCCTCCCAG :  720
AIT_TRLF1e : AACCTGTTGCTCAACGCCAACTGCGACCTCAAGGTCTGCGACTTCGGTCTGGCCCGGTCCGCCGCCTCCCAG :  720
AIT_TRKH1a : AACCTGTTGCTCAACGCCAACTGCGACCTCAAGGTCTGCGACTTCGGTCTGGCCCGGTCCGCCGCCTCCCAG :  720
CBS999.97  : AACCTGTTGCTCAACGCCAACTGCGACCTCAAGGTCTGCGACTTCGGTCTGGCCCGGTCCGCTGCCTCCCAG :  720
AIT_TRKH1c : AACCTGTTGCTCAACGCCAACTGCGACCTCAAGGTCTGCGACTTCGGTCTGGCCCGGTCCGCTGCCTCCCAG :  720
AIT_TRMS44 : AACCTGTTGCTCAACGCCAACTGCGACCTCAAGGTCTGCGACTTCGGTCTGGCCCGGTCCGCTGCCTCCCAG :  720
             AACCTGTTGCTCAACGCCAACTGCGACCTCAAGGTCTGCGACTTCGGTCTGGCCCGGTCCGC GCCTCCCAG

                        *       740         *       760         *       780         *
QM6a       : GAGGACAACTCGGGCTTCATGACGGAATATGTCGCCACGCGATGGTACCGCGCGCCCGAGATCATGTTGACC :  792
RUTC30     : GAGGACAACTCGGGCTTCATGACGGAATATGTCGCCACGCGATGGTACCGCGCGCCCGAGATCATGTTGACC :  792
AIT_TRLF1e : GAGGACAACTCGGGCTTCATGACGGAATATGTCGCCACGCGATGGTACCGCGCGCCCGAGATCATGTTGACC :  792
AIT_TRKH1a : GAGGACAACTCGGGCTTCATGACGGAATATGTCGCCACGCGATGGTACCGCGCGCCCGAGATCATGTTGACC :  792
CBS999.97  : GAGGACAACTCGGGCTTCATGACGGAATATGTCGCCACGCGATGGTACCGCGCGCCCGAGATCATGTTGACC :  792
AIT_TRKH1c : GAGGACAACTCGGGCTTCATGACGGAATATGTCGCCACGCGATGGTACCGCGCGCCCGAGATCATGTTGACC :  792
AIT_TRMS44 : GAGGACAACTCGGGCTTCATGACGGAATATGTCGCCACGCGATGGTACCGCGCGCCCGAGATCATGTTGACC :  792
             GAGGACAACTCGGGCTTCATGACGGAATATGTCGCCACGCGATGGTACCGCGCGCCCGAGATCATGTTGACC

                    800         *       820         *       840         *       860
QM6a       : TTCAAGGAGTACACCAAGGCCATTGATGTCTGGTCCGTGGGCTGCATCCTGGCTGAGATGCTCAGCGGCAAG :  864
RUTC30     : TTCAAGGAGTACACCAAGGCCATTGATGTCTGGTCCGTGGGCTGCATCCTGGCTGAGATGCTCAGCGGCAAG :  864
AIT_TRLF1e : TTCAAGGAGTACACCAAGGCCATTGATGTCTGGTCCGTGGGCTGCATCCTGGCTGAGATGCTCAGCGGCAAG :  864
AIT_TRKH1a : TTCAAGGAGTACACCAAGGCCATTGATGTCTGGTCCGTGGGCTGCATCCTGGCTGAGATGCTCAGCGGCAAG :  864
CBS999.97  : TTCAAGGAGTACACCAAGGCCATTGACGTCTGGTCCGTGGGCTGCATCCTGGCTGAGATGCTCAGCGGCAAG :  864
AIT_TRKH1c : TTCAAGGAGTACACCAAGGCCATTGACGTCTGGTCCGTGGGCTGCATCCTGGCTGAGATGCTCAGCGGCAAG :  864
AIT_TRMS44 : TTCAAGGAGTACACCAAGGCCATTGACGTCTGGTCCGTGGGCTGCATCCTGGCTGAGATGCTCAGCGGCAAG :  864
             TTCAAGGAGTACACCAAGGCCATTGA GTCTGGTCCGTGGGCTGCATCCTGGCTGAGATGCTCAGCGGCAAG

                    *       880         *       900         *       920         *
QM6a       : CCTCTGTTCCCTGGCAAGGATTGTAAGTTGGCATTGCCCGGAGCGAGATCTATGCGAAAAAATCAAGTTGGC :  936
RUTC30     : CCTCTGTTCCCTGGCAAGGATTGTAAGTTGGCATTGCCCGGAGCGAGATCTATGCGAAAAAATCAAGTTGGC :  936
AIT_TRLF1e : CCTCTGTTCCCTGGCAAGGATTGTAAGTTGGCATTGCCCGGAGCGAGATCTATGCGAAAAAATCAAGTTGGC :  936
AIT_TRKH1a : CCTCTGTTCCCTGGCAAGGATTGTAAGTTGGCATTGCCCGGAGCGAGATCTATGCGAAAAAATCAAGTTGGC :  936
CBS999.97  : CCTCTGTTTCCTGGCAAGGATTGTAAGTTGGCATTGTCCGGAGCGAGATCCATGCGAAAAAATCAAGCTGGC :  936
AIT_TRKH1c : CCTCTGTTTCCTGGCAAGGATTGTAAGTTGGCATTGTCCGGAGCGAGATCCATGCGAAAAAATCAAGCTGGC :  936
AIT_TRMS44 : CCTCTGTTTCCTGGCAAGGATTGTAAGTTGGCATTGTCCGGAGCGAGATCCATGCGAAAAAATCAAGCTGGC :  936
             CCTCTGTT CCTGGCAAGGATTGTAAGTTGGCATTG CCGGAGCGAGATC ATGCGAAAAAATCAAG TGGC

                940         *       960         *       980         *      1000
QM6a       : TAACACAGATAACCCTCCAGACCACCACCAGCTGACTCTGATCCTGGATGTGCTCGGCACGCCCCACCATGGA : 1008
RUTC30     : TAACACAGATAACCCTCCAGACCACCACCAGCTGACTCTGATCCTGGATGTGCTCGGCACGCCCCACCATGGA : 1008
AIT_TRLF1e : TAACACAGATAACCCTCCAGACCACCACCAGCTGACTCTGATCCTGGATGTGCTCGGCACGCCCCACCATGGA : 1008
AIT_TRKH1a : TAACACAGATAACCCTCCAGACCACCACCAGCTGACTCTGATCCTGGATGTGCTCGGCACGCCCCACCATGGA : 1008
CBS999.97  : TAACACAGAAAACCCTCCAGACCACCACCAGCTGACTCTGATCCTGGATGTGCTCGGCACGCCCCACCATGGA : 1008
AIT_TRKH1c : TAACACAGAAAACCCTCCAGACCACCACCAGCTGACTCTGATCCTGGATGTGCTCGGCACGCCCCACCATGGA : 1008
AIT_TRMS44 : TAACACAGAAAACCCTCCAGACCACCACCAGCTGACTCTGATCCTGGATGTGCTCGGCACGCCCCACCATGGA : 1008
             TAACACAGA AACCCTCCAGACCACCACCAGCTGACTCTGATCCTGGATGTGCTCGGCACGCCCCACCATGGA

                *      1020         *      1040         *      1060         *      1080
QM6a       : GGACTACTATGGCATCAAATCTCGACGAGCGAGGGAGTACATCCGCTCGCTGCCCTTCAAGAAGAAGGTGCC : 1080
RUTC30     : GGACTACTATGGCATCAAATCTCGACGAGCGAGGGAGTACATCCGCTCGCTGCCCTTCAAGAAGAAGGTGCC : 1080
AIT_TRLF1e : GGACTACTATGGCATCAAATCTCGACGAGCGAGGGAGTACATCCGCTCGCTGCCCTTCAAGAAGAAGGTGCC : 1080
AIT_TRKH1a : GGACTACTATGGCATCAAATCTCGACGAGCGAGGGAGTACATCCGCTCGCTGCCCTTCAAGAAGAAGGTGCC : 1080
CBS999.97  : GGACTACTATGGCATCAAGTCTCGACGAGCGAGGGAGTACATCCGCTCGCTGCCCTTCAAGAAGAAGGTGCC : 1080
AIT_TRKH1c : GGACTACTATGGCATCAAGTCTCGACGAGCGAGGGAGTACATCCGCTCGCTGCCCTTCAAGAAGAAGGTGCC : 1080
AIT_TRMS44 : GGACTACTATGGCATCAAGTCTCGACGAGCGAGGGAGTACATCCGCTCGCTGCCCTTCAAGAAGAAGGTGCC : 1080
             GGACTACTATGGCATCAA TCTCGACGAGCGAGGGAGTACATCCGCTCGCTGCCCTTCAAGAAGAAGGTGCC

                        *      1100         *      1120         *      1140         *
QM6a       : CTTCCGCACCTTGTTCCCCAAGACGTCCGACCTGGCCCTGGACCTGCTCGAGAAGCTGCTCGCGTTCAACCC : 1152
RUTC30     : CTTCCGCACCTTGTTCCCCAAGACGTCCGACCTGGCCCTGGACCTGCTCGAGAAGCTGCTCGCGTTCAACCC : 1152
AIT_TRLF1e : CTTCCGCACCTTGTTCCCCAAGACGTCCGACCTGGCCCTGGACCTGCTCGAGAAGCTGCTCGCGTTCAACCC : 1152
AIT_TRKH1a : CTTCCGCACCTTGTTCCCCAAGACGTCCGACCTGGCCCTGGACCTGCTCGAGAAGCTGCTCGCGTTCAACCC : 1152
CBS999.97  : CTTCCGCACCTTGTTCCCCAAGACGTCCGACCTGGCCCTGGACCTGCTCGAGAAGCTGCTCGCATTCAACCC : 1152
AIT_TRKH1c : CTTCCGCACCTTGTTCCCCAAGACGTCCGACCTGGCCCTGGACCTGCTCGAGAAGCTGCTCGCATTCAACCC : 1152
```

```
AIT_TRMS44 : CTTCCGCACCTTGTTCCCCAAGACGTCCGACCTGGCCCTGGACCTGCTCGAGAAGCTGCTCGCATTCAACCC : 1152
             CTTCCGCACCTTGTTCCCCAAGACGTCCGACCTGGCCCTGGACCTGCTCGAGAAGCTGCTCGC TTCAACCC

                 1160         *      1180         *      1200         *      1220
QM6a       : GGTGAAGCGAATCACGGTGGAGGACGCCCTCAAGCACCCGTACCTCGAGCCCTACCACGACCCGGATGACGA : 1224
RUTC30     : GGTGAAGCGAATCACGGTGGAGGACGCCCTCAAGCACCCGTACCTCGAGCCCTACCACGACCCGGATGACGA : 1224
AIT_TRLF1e : GGTGAAGCGAATCACGGTGGAGGACGCCCTCAAGCACCCGTACCTCGAGCCCTACCACGACCCGGATGACGA : 1224
AIT_TRKH1a : GGTGAAGCGAATCACGGTGGAGGACGCCCTCAAGCACCCGTACCTCGAGCCCTACCACGACCCGGATGACGA : 1224
CBS999.97  : GGTGAAGCGAATCACGGTGGAGGACGCCCTCAAGCACCCGTACCTCGAGCCCTACCACGACCCGGATGACGA : 1224
AIT_TRKH1c : GGTGAAGCGAATCACGGTGGAGGACGCCCTCAAGCACCCGTACCTCGAGCCCTACCACGACCCGGATGACGA : 1224
AIT_TRMS44 : GGTGAAGCGAATCACGGTGGAGGACGCCCTCAAGCACCCGTACCTCGAGCCCTACCACGACCCGGATGACGA : 1224
             GGTGAAGCGAATCACGGTGGAGGACGCCCTCAAGCACCCGTACCTCGAGCCCTACCACGACCCGGATGACGA

               *      1240         *      1260         *      1280         *
QM6a       : GCCCACCGCGCCTCCGATTCCCGAGGAGTTTTTCGATTTCGACAAGCACAAAGATACCCTGAGCAAGGAGCA : 1296
RUTC30     : GCCCACCGCGCCTCCGATTCCCGAGGAGTTTTTCGATTTCGACAAGCACAAAGATACCCTGAGCAAGGAGCA : 1296
AIT_TRLF1e : GCCCACCGCGCCTCCGATTCCCGAGGAGTTTTTCGATTTCGACAAGCACAAAGATACCCTGAGCAAGGAGCA : 1296
AIT_TRKH1a : GCCCACCGCGCCTCCGATTCCCGAGGAGTTTTTCGATTTCGACAAGCACAAAGATACCCTGAGCAAGGAGCA : 1296
CBS999.97  : GCCCACCGCGCCTCCGATTCCCGAGGAGTTTTTCGATTTCGACAAGCACAAAGATACCCTGAGCAAGGAGCA : 1296
AIT_TRKH1c : GCCCACCGCGCCTCCGATTCCCGAGGAGTTTTTCGATTTCGACAAGCACAAAGATACCCTGAGCAAGGAGCA : 1296
AIT_TRMS44 : GCCCACCGCGCCTCCGATTCCCGAGGAGTTTTTCGATTTCGACAAGCACAAAGATACCCTGAGCAAGGAGCA : 1296
             GCCCACCGCGCCTCCGATTCCCGAGGAGTTTTTCGATTTCGACAAGCACAAAGATACCCTGAGCAAGGAGCA

             1300         *      1320         *
QM6a       : GCTGAAGCAACTGATTTACCAGGAGATTATGCGGTAA : 1333
RUTC30     : GCTGAAGCAACTGATTTACCAGGAGATTATGCGGTAA : 1333
AIT_TRLF1e : GCTGAAGCAACTGATTTACCAGGAGATTATGCGGTAA : 1333
AIT_TRKH1a : GCTGAAGCAACTGATTTACCAGGAGATTATGCGGTAA : 1333
CBS999.97  : GCTGAAGCAACTGATTTACCAGGAGATTATGCGGTAA : 1333
AIT_TRKH1c : GCTGAAGCAACTGATTTACCAGGAGATTATGCGGTAA : 1333
AIT_TRMS44 : GCTGAAGCAACTGATTTACCAGGAGATTATGCGGTAA : 1333
             GCTGAAGCAACTGATTTACCAGGAGATTATGCGGTAA
```

Sequence alignment *tmk3*

```
                     *        20         *        40         *        60         *
AIT_TRKH1a : ATGGCCGAGTTTGTGCGAGCGCAGATCTTTGGCACCACCTTCGAGATCACCTCACGGTAAGGCCAGCCAGCA :   72
AIT_TRLF1e : ATGGCCGAGTTTGTGCGAGCGCAGATCTTTGGCACCACCTTCGAGATCACCTCACGGTAAGGCCAGCCAGCA :   72
QM6a       : ATGGCCGAGTTTGTGCGAGCGCAGATCTTTGGCACCACCTTCGAGATCACCTCACGGTAAGGCCAGCCAGCA :   72
RUTC30     : ATGGCCGAGTTTGTGCGAGCGCAGATCTTTGGCACCACCTTCGAGATCACCTCACGGTAAGGCCAGCCAGCA :   72
AIT_TRKH1c : ATGGCCGAGTTTGTGCGAGCGCAGATCTTTGGCACCACCTTCGAGATCACCTCACGGTAAGGCCAGCCAGCA :   72
CBS999.97  : ATGGCCGAGTTTGTGCGAGCGCAGATCTTTGGCACCACCTTCGAGATCACCTCACGGTAAGGCCAGCCAGCA :   72
AIT_TRMS44 : ATGGCCGAGTTTGTGCGAGCGCAGATCTTTGGCACCACCTTCGAGATCACCTCACGGTAAGGCCAGCCAGCA :   72
             ATGGCCGAGTTTGTGCGAGCGCAGATCTTTGGCACCACCTTCGAGATCACCTCACGGTAAGGCCAGCCAGCA

                  80         *       100         *       120         *       140
AIT_TRKH1a : GCCAGCAGCCAGGGAGTCCGCGCCCGACCGGATCACGCAGGCGGCGTTGACGAGACGCAATTTGAAGCTGAC :  144
AIT_TRLF1e : GCCAGCAGCCAGGGAGTCCGCGCCCGACCGGATCACGCAGGCGGCGTTGACGAGACGCAATTTGAAGCTGAC :  144
QM6a       : GCCAGCAGCCAGGGAGTCCGCGCCCGACCGGATCACGCAGGCGGCGTTGACGAGACGCAATTTGAAGCTGAC :  144
RUTC30     : GCCAGCAGCCAGGGAGTCCGCGCCCGACCGGATCACGCAGGCGGCGTTGACGAGACGCAATTTGAAGCTGAC :  144
AIT_TRKH1c : GCCAGCAGCCAGGGAGTCCGCGCCCGACCGGATCACGCAGGCGGCGTTGACGAGACGCAATTTGAAGCTGAC :  144
CBS999.97  : GCCAGCAGCCAGGGAGTCCGCGCCCGACCGGATCACGCAGGCGGCGTTGACGAGACGCAATATGAGGCTGAC :  144
AIT_TRMS44 : GCCAGCAGCCAGGGAGTCCGCGCCCGACCGGATCACGCAGGCGGCGTTGACGAGACGCAATATGAGGCTGAC :  144
             GCCAGCAGCCAGGGAGTCCGCGCCCGACCGGATCACGCAGGCGGCGTTGACGAGACGCAAT TGA GCTGAC

               *       160         *       180         *       200         *
AIT_TRKH1a : TTGACAATTCGTCTAGGTACTCGGACCTCCAGCCCGTGGGCATGGGAGCCTTTGGCCTCGTCTGGTGAGTTT :  216
AIT_TRLF1e : TTGACAATTCGTCTAGGTACTCGGACCTCCAGCCCGTGGGCATGGGAGC-----------TCTGGTGAGTTT :  205
QM6a       : TTGACAATTCGTCTAGGTACTCGGACCTCCAGCCCGTGGGCATGGGAGCCTTTGGCCTCGTCTGGTGAGTTT :  216
RUTC30     : TTGACAATTCGTCTAGGTACTCGGACCTCCAGCCCGTGGGCATGGGAGCCTTTGGCCTCGTCTGGTGAGTTT :  216
AIT_TRKH1c : TTGACAATTCGTCTAGGTACTCGGACCTCCAGCCCGTGGGCATGGGAGCCTTTGGCCTCGTCTGGTGAGTTT :  216
CBS999.97  : TTGACAATTCGTCCAGGTACTCGGACCTCCAGCCCGTGGGCATGGGAGCCTTTGGCCTCGTCTGGTGAGTTT :  216
AIT_TRMS44 : TTGACAATTCGTCCAGGTACTCGGACCTCCAGCCCGTGGGCATGGGAGCCTTTGGCCTCGTCTGGTGAGTTT :  216
             TTGACAATTCGTC AGGTACTCGGACCTCCAGCCCGTGGGCATGGGAGCctttggcctcgTCTGGTGAGTTT

              220         *       240         *       260         *       280
AIT_TRKH1a : TCGCCTCCCCCCTCTC---------CCCCCCTCCTCCGTCCAATTGTCAAGATGGCCTTTTCTGCGGCCGGG :  279
AIT_TRLF1e : TCGCCTCCCCCCTCTC---------CCCCCCTCCTCCGTCCAATTGTCAAGATGGCCTTTTCTGCGGCCGGG :  268
QM6a       : TCGCCTCCCCCCTCTC---------CCCCCCTCCTCCGTCCAATTGTCAAGATGGCCTTTTCTGCGGCCGGG :  279
RUTC30     : TCGCCTCCCCCCTCTC---------CCCCCCTCCTCCGTCCAATTGTCAAGATGGCCTTTTCTGCGGCCGGG :  279
AIT_TRKH1c : TCGCCTCCCCCCTCTCNNNNNNNNNNCCCCCTCCTCCGTCCAATTGTCAAGATGGCCTTTTCTGCGGCCGGG :  288
CBS999.97  : TCGCCTCCCCCCTCTC---------CCCCC-TCCTCCGTCCAATTGTCAAGATGGCCTTTTCTGCGGCCGGG :  278
AIT_TRMS44 : TCGCCTCCCCCCTCTC---------CCCCCCTCCTCCGTCCAATTGTCAAGATGGCCTTTTCTGCGGCCGGG :  279
             TCGCCTCCCCCCTCTC         CCCCCcTCCTCCGTCCAATTGTCAAGATGGCCTTTTCTGCGGCCGGG

              *       300         *       320         *       340         *       360
```

```
AIT_TRKH1a : CGAATCGAACCCATGCTAATTCGCGGCGCTTCCCCTCCCCCGCCAAACAGCTCTGCGCGAGACCAGCTCACC :  351
AIT_TRLF1e : CGAATCGAACCCATGCTAATTCGCGGCGCTTCCCCTCCCCCGCCAAACAGCTCTGCGCGAGACCAGCTCACC :  340
QM6a       : CGAATCGAACCCATGCTAATTCGCGGCGCTTCCCCTCCCCCGCCAAACAGCTCTGCGCGAGACCAGCTCACC :  351
RUTC30     : CGAATCGAACCCATGCTAATTCGCGGCGCTTCCCCTCCCCCGCCAAACAGCTCTGCGCGAGACCAGCTCACC :  351
AIT_TRKH1c : CGAATCGAACCCATGCTAATTCGCGGCGCTTCCCCTCCCCCGCCAAACAGCTCTGCGCGAGACCAGCTCACC :  360
CBS999.97  : CGAATCGAACCCATGCTAATTCGCGGCGCTTCCCCTCCCCCGCCAAACAGCTCTGCGCGAGACCAGCTCACC :  350
AIT_TRMS44 : CGAATCGAACCCATGCTAATTCGCGGCGCTTCCCCTCCCCCGCCAAACAGCTCTGCGCGAGACCAGCTCACC :  351
             CGAATCGAACCCATGCTAATTCGCGGCGCTTCCCCTCCCCCGCCAAACAGCTCTGCGCGAGACCAGCTCACC

                      *       380         *       400         *       420         *
AIT_TRKH1a : AACCAAAATGTCGCCGTCAAGAAGATCATGAAGCCCTTCAGCACGCCCGTACTCGCTAAGCGGACGTACCGT :  423
AIT_TRLF1e : AACCAAAATGTCGCCGTCAAGAAGATCATGAAGCCCTTCAGCACGCCCGTACTCGCTAAGCGGACGTACCGT :  412
QM6a       : AACCAAAATGTCGCCGTCAAGAAGATCATGAAGCCCTTCAGCACGCCCGTACTCGCTAAGCGGACGTACCGT :  423
RUTC30     : AACCAAAATGTCGCCGTCAAGAAGATCATGAAGCCCTTCAGCACGCCCGTACTCGCTAAGCGGACGTACCGT :  423
AIT_TRKH1c : AACCAAAATGTCGCCGTCAAGAAGATCATGAAGCCCTTCAGCACGCCCGTACTCGCTAAGCGGACGTACCGT :  432
CBS999.97  : AACCAAAATGTCGCCGTCAAGAAGATCATGAAGCCCTTCAGCACGCCCGTACTCGCTAAGCGGACGTACCGT :  422
AIT_TRMS44 : AACCAAAATGTCGCCGTCAAGAAGATCATGAAGCCCTTCAGCACGCCCGTACTCGCTAAGCGGACGTACCGT :  423
             AACCAAAATGTCGCCGTCAAGAAGATCATGAAGCCCTTCAGCACGCCCGTACTCGCTAAGCGGACGTACCGT

                  440         *       460         *       480         *       500
AIT_TRKH1a : GAGCTGAAGCTGCTCAAGCACCTCCGACACGAAAATGTATGCGCTGTGCCTCGATTTTTTTTTTCT-TTTTT :  494
AIT_TRLF1e : GAGCTGAAGCTGCTCAAGCACCTCCGACACGAAAATGTATGCGCTGTGCCTCGATTTTTTTTTTCT-TTTTT :  483
QM6a       : GAGCTGAAGCTGCTCAAGCACCTCCGACACGAAAATGTATGCGCTGTGCCTCGATTTTTTTTTTCT-TTTTT :  494
RUTC30     : GAGCTGAAGCTGCTCAAGCACCTCCGACACGAAAATGTATGCGCTGTGCCTCGATTTTTTTTTTCT-TTTTT :  494
AIT_TRKH1c : GAGCTGAAGCTGCTCAAGCACCTCCGACACGAAAATGTATGCGCTGTGCCTCGATTTTTTTTTTCT-TTTTT :  503
CBS999.97  : GAGCTGAAGCTGCTCAAGCACCTCCGACACGAAAATGTATGCGCTATCCCTCGTTTTTTTTTTTTTNTTTTT :  494
AIT_TRMS44 : GAGCTGAAGCTGCTCAAGCACCTCCGACACGAAAATGTATGCGCTATCCCTCGANTTTTTTTTTT-TTTTT :  494
             GAGCTGAAGCTGCTCAAGCACCTCCGACACGAAAATGTATGCGCT T CCTCGaTTTTTTTTTT T TTTTT

                  *       520         *       540         *       560         *
AIT_TRKH1a : TGATGGGCGATTTATTGCATCGCTGGGTTCGGCTGACGATTGACGTCCGCTGGATCTGGGCCTGCCCATAAG :  566
AIT_TRLF1e : TGATGGGCGATTTATTGCATCGCTGGGTTCGGCTGACGATTGACGTCCGCTGGATCTGGGCCTGCCCATAAG :  555
QM6a       : TGATGGGCGATTTATTGCATCGCTGGGTTCGGCTGACGATTGACGTCCGCTGGATCTGGGCCTGCCCATAAG :  566
RUTC30     : TGATGGGCGATTTATTGCATCGCTGGGTTCGGCTGACGATTGACGTCCGCTGGATCTGGGCCTGCCCATAAG :  566
AIT_TRKH1c : TGATGGGCGATTTATTGCATCGCTGGGTTCGGCTGACGATTGACGTCCGCTGGATCTGGGCCTGCCCATAAG :  575
CBS999.97  : TGATGGGCGATTTATTGCATCGCTGGGTTCGGCTGACGAT-GACGTCCGCTGGATCTGGGCCTGCCCATAAG :  565
AIT_TRMS44 : TGATGGGCGATTTATTGCATCGCTGGGTTCGGCTGACGA-TGACGTCCGCTGGATCTGGGCCTGCCCATAAG :  565
             TGATGGGCGATTTATTGCATCGCTGGGTTCGGCTGACGAttGACGTCCGCTGGATCTGGGCCTGCCCATAAG

              580         *       600         *       620         *       640
AIT_TRKH1a : CTACTACTTGCGTGGTGCTGACACTGCGATGCTGCATAGGTCATTTCTCTCAGCGACATCTTCATCTCTCCC :  638
AIT_TRLF1e : CTACTACTTGCGTGGTGCTGACACTGCGATGCTGCATAGGTCATTTCTCTCAGCGACATCTTCATCTCTCCC :  627
QM6a       : CTACTACTTGCGTGGTGCTGACACTGCGATGCTGCATAGGTCATTTCTCTCAGCGACATCTTCATCTCTCCC :  638
RUTC30     : CTACTACTTGCGTGGTGCTGACACTGCGATGCTGCATAGGTCATTTCTCTCAGCGACATCTTCATCTCTCCC :  638
AIT_TRKH1c : CTACTACTTGCGTGGTGCTGACACTGCGATGCTGCATAGGTCATTTCTCTCAGCGACATCTTCATCTCTCCC :  647
CBS999.97  : CTACTACTTGCGTGGTGCTGACACTGCGATGCTGCATAGGTCATCTCTCTCAGCGACATCTTCATCTCTCCC :  637
AIT_TRMS44 : CTACTACTTGCGTGGTGCTGACACTGCGATGCTGCATAGGTCATCTCTCTCAGCGACATCTTCATCTCTCCC :  637
             CTACTACTTGCGTGGTGCTGACACTGCGATGCTGCATAGGTCAT TCTCTCAGCGACATCTTCATCTCTCCC

              *       660         *       680         *       700         *       720
AIT_TRKH1a : CTCGAGGACATGTACGTATAATTGCATGCATAGTCTTGGTTGATGTTGTGCCGTTCTAACATTGTTTCAGCT :  710
AIT_TRLF1e : CTCGAGGACATGTACGTATAATTGCATGCATAGTCTTGGTTGATGTTGTGCCGTTCTAACATTGTTTCAGCT :  699
QM6a       : CTCGAGGACATGTACGTATAATTGCATGCATAGTCTTGGTTGATGTTGTGCCGTTCTAACATTGTTTCAGCT :  710
RUTC30     : CTCGAGGACATGTACGTATAATTGCATGCATAGTCTTGGTTGATGTTGTGCCGTTCTAACATTGTTTCAGCT :  710
AIT_TRKH1c : CTCGAGGACATGTACGTATAATTGCATGCATAGTCTTGGTTGATGTTGTGCCGTTCTAACATTGTTTCAGCT :  719
CBS999.97  : CTCGAGGACATGTACGTATAATTGCATGCATAGTCTTGGTTGATGTTGTGCCGTTCTAACATTGTTTCAGCT :  709
AIT_TRMS44 : CTCGAGGACATGTACGTATAATTGCATGCATAGTCTTGGTTGATGTTGTGCCGTTCTAACATTGTTTCAGCT :  709
             CTCGAGGACATGTACGTATAATTGCATGCATAGTCTTGGTTGATGTTGTGCCGTTCTAACATTGTTTCAGCT

                      *       740         *       760         *       780         *
AIT_TRKH1a : ACTTCGTCACAGAGCTTCTTGGCACCGACTTGCACCGATTATTAACCTCCCGACCCCTCGAGAAACAATTCA :  782
AIT_TRLF1e : ACTTCGTCACAGAGCTTCTTGGCACCGACTTGCACCGATTATTAACCTCCCGACCCCTCGAGAAACAATTCA :  771
QM6a       : ACTTCGTCACAGAGCTTCTTGGCACCGACTTGCACCGATTATTAACCTCCCGACCCCTCGAGAAACAATTCA :  782
RUTC30     : ACTTCGTCACAGAGCTTCTTGGCACCGACTTGCACCGATTATTAACCTCCCGACCCCTCGAGAAACAATTCA :  782
AIT_TRKH1c : ACTTCGTCACAGAGCTTCTTGGCACCGACTTGCACCGATTATTAACCTCCCGACCCCTCGAGAAACAATTCA :  791
CBS999.97  : ACTTCGTCACAGAGCTTCTTGGCACCGACTTGCACCGATTATTAACCTCCCGACCCCTCGAGAAACAATTCA :  781
AIT_TRMS44 : ACTTCGTCACAGAGCTTCTTGGCACCGACTTGCACCGATTATTAACCTCCCGACCCCTCGAGAAACAATTCA :  781
             ACTTCGTCACAGAGCTTCTTGGCACCGACTTGCACCGATTATTAACCTCCCGACCCCTCGAGAAACAATTCA

                  800         *       820         *       840         *       860
AIT_TRKH1a : TCCAATACTTCCTCTACCAGATCATGGTATGCGATGGCACAACCCCCCCCTTGGCCAGCGGCAACCAGCATG :  854
AIT_TRLF1e : TCCAATACTTCCTCTACCAGATCATGGTATGCGATGGCACAACCCCCCCCTTGGCCAGCGGCAACCAGCATG :  843
QM6a       : TCCAATACTTCCTCTACCAGATCATGGTATGCGATGGCACAACCCCCCCCTTGGCCAGCGGCAACCAGCATG :  854
RUTC30     : TCCAATACTTCCTCTACCAGATCATGGTATGCGATGGCACAACCCCCCCCTTGGCCAGCGGCAACCAGCATG :  854
AIT_TRKH1c : TCCAATACTTCCTCTACCAGATCATGGTATGCGATGGCACAACCCCCCCCTTGGCCAGCGGCAACCAGCATG :  863
CBS999.97  : TCCAATACTTCCTCTACCAGATCATGGTATGCGATGGCACAACCCCCCCATTGGCCAGCGGCAACCAGCATG :  853
AIT_TRMS44 : TCCAATACTTCCTCTACCAGATCATGGTATGCGATGGCACAACCCCCCCATTGGCCAGCGGCAACCAGCATG :  853
             TCCAATACTTCCTCTACCAGATCATGGTATGCGATGGCACAACCCCCCC TTGGCCAGCGGCAACCAGCATG

                  *       880         *       900         *       920         *
AIT_TRKH1a : ATTCCAAAATAAAGCCCTGCAGCTAACAGATGCGTGTCTCATAGCGAGGCCTGAAATATGTCCACTCGGCCG :  926
AIT_TRLF1e : ATTCCAAAATAAAGCCCTGCAGCTAACAGATGCGTGTCTCATAGCGAGGCCTGAAATATGTCCACTCGGCCG :  915
```

```
QM6a        : ATTCCAAAATAAAGCCCTGCAGCTAACAGATGCGTGTCTCATAGCGAGGCCTGAAATATGTCCACTCGGCCG :  926
RUTC30      : ATTCCAAAATAAAGCCCTGCAGCTAACAGATGCGTGTCTCATAGCGAGGCCTGAAATATGTCCACTCGGCCG :  926
AIT_TRKH1c  : ATTCCAAAATAAAGCCCTGCAGCTAACAGATGCGTGTCTCATAGCGAGGCCTGAAATATGTCCACTCGGCCG :  935
CBS999.97   : ATTCCAAAATAGAGCCCTGCAGCTAACAGATGCGTGTCTCATAGCGAGGCCTGAAATATGTCCACTCGGCCG :  925
AIT_TRMS44  : ATTCCAAAATAGAGCCCTGCAGCTAACAGATGCGTGTCTCATAGCGAGGCCTGAAATATGTCCACTCGGCCG :  925
              ATTCCAAAATA AGCCCTGCAGCTAACAGATGCGTGTCTCATAGCGAGGCCTGAAATATGTCCACTCGGCCG

                940         *       960         *       980         *      1000
AIT_TRKH1a  : GCGTCGTCCACCGCGATCTCAAGCCCAGCAACATCCTCGTCAACGAAAACTGCGATCTCAAGATTTGCGACT :  998
AIT_TRLF1e  : GCGTCGTCCACCGCGATCTCAAGCCCAGCAACATCCTCGTCAACGAAAACTGCGATCTCAAGATTTGCGACT :  987
QM6a        : GCGTCGTCCACCGCGATCTCAAGCCCAGCAACATCCTCGTCAACGAAAACTGCGATCTCAAGATTTGCGACT :  998
RUTC30      : GCGTCGTCCACCGCGATCTCAAGCCCAGCAACATCCTCGTCAACGAAAACTGCGATCTCAAGATTTGCGACT :  998
AIT_TRKH1c  : GCGTCGTCCACCGCGATCTCAAGCCCAGCAACATCCTCGTCAACGAAAACTGCGATCTCAAGATTTGCGACT : 1007
CBS999.97   : GCGTCGTCCACCGCGATCTCAAGCCCAGCAACATCCTCGTCAACGAAAACTGCGATCTCAAGATTTGCGACT :  997
AIT_TRMS44  : GCGTCGTCCACCGCGATCTCAAGCCCAGCAACATCCTCGTCAACGAAAACTGCGATCTCAAGATTTGCGACT :  997
              GCGTCGTCCACCGCGATCTCAAGCCCAGCAACATCCTCGTCAACGAAAACTGCGATCTCAAGATTTGCGACT

                *      1020         *      1040         *      1060         *      1080
AIT_TRKH1a  : TTGGTCTTGCCCGAATCCAGGACCCGCAGATGACGGGCTATGTTTCAACACGATACTACCGCGCCCCCGAGA : 1070
AIT_TRLF1e  : TTGGTCTTGCCCGAATCCAGGACCCGCAGATGACGGGCTATGTTTCAACACGATACTACCGCGCCCCCGAGA : 1059
QM6a        : TTGGTCTTGCCCGAATCCAGGACCCGCAGATGACGGGCTATGTTTCAACACGATACTACCGCGCCCCCGAGA : 1070
RUTC30      : TTGGTCTTGCCCGAATCCAGGACCCGCAGATGACGGGCTATGTTTCAACACGATACTACCGCGCCCCCGAGA : 1070
AIT_TRKH1c  : TTGGTCTTGCCCGAATCCAGGACCCGCAGATGACGGGCTATGTTTCAACACGATACTACCGCGCCCCCGAGA : 1079
CBS999.97   : TTGGTCTTGCCCGAATCCAGGACCCGCAGATGACGGGCTATGTTTCAACACGATACTACCGCGCCCCCGAGA : 1069
AIT_TRMS44  : TTGGTCTTGCCCGAATCCAGGACCCGCAGATGACGGGCTATGTTTCAACACGATACTACCGCGCCCCCGAGA : 1069
              TTGGTCTTGCCCGAATCCAGGACCCGCAGATGACGGGCTATGTTTCAACACGATACTACCGCGCCCCCGAGA

                        *      1100         *      1120         *      1140         *
AIT_TRKH1a  : TCATGCTCACGTGGCAAAAGTACGACGTCGAGGTCGACATCTGGAGCGCCGGGTGCATCTTCGCCGAGATGC : 1142
AIT_TRLF1e  : TCATGCTCACGTGGCAAAAGTACGACGTCGAGGTCGACATCTGGAGCGCCGGGTGCATCTTCGCCGAGATGC : 1131
QM6a        : TCATGCTCACGTGGCAAAAGTACGACGTCGAGGTCGACATCTGGAGCGCCGGGTGCATCTTCGCCGAGATGC : 1142
RUTC30      : TCATGCTCACGTGGCAAAAGTACGACGTCGAGGTCGACATCTGGAGCGCCGGGTGCATCTTCGCCGAGATGC : 1142
AIT_TRKH1c  : TCATGCTCACGTGGCAAAAGTACGACGTCGAGGTCGACATCTGGAGCGCCGGGTGCATCTTCGCCGAGATGC : 1151
CBS999.97   : TCATGCTCACGTGGCAAAAGTACGACGTCGAGGTCGACATCTGGAGCGCCGGGTGCATCTTCGCCGAGATGC : 1141
AIT_TRMS44  : TCATGCTCACGTGGCAAAAGTACGACGTCGAGGTCGACATCTGGAGCGCCGGGTGCATCTTCGCCGAGATGC : 1141
              TCATGCTCACGTGGCAAAAGTACGACGTCGAGGTCGACATCTGGAGCGCCGGGTGCATCTTCGCCGAGATGC

                  1160         *      1180         *      1200         *      1220
AIT_TRKH1a  : TCGAGGGTAAGCCCCTGTTCCCGGGCAAGGACCACGTGAACCAGTTCTCCATCATCACCGAGCTGCTGGGCA : 1214
AIT_TRLF1e  : TCGAGGGTAAGCCCCTGTTCCCGGGCAAGGACCACGTGAACCAGTTCTCCATCATCACCGAGCTGCTGGGCA : 1203
QM6a        : TCGAGGGTAAGCCCCTGTTCCCGGGCAAGGACCACGTGAACCAGTTCTCCATCATCACCGAGCTGCTGGGCA : 1214
RUTC30      : TCGAGGGTAAGCCCCTGTTCCCGGGCAAGGACCACGTGAACCAGTTCTCCATCATCACCGAGCTGCTGGGCA : 1214
AIT_TRKH1c  : TCGAGGGTAAGCCCCTGTTCCCGGGCAAGGACCACGTGAACCAGTTCTCCATCATCACCGAGCTGCTGGGCA : 1223
CBS999.97   : TCGAGGGTAAGCCCCTGTTCCCGGGCAAGGACCACGTGAACCAGTTCTCCATCATCACCGAGCTGCTGGGCA : 1213
AIT_TRMS44  : TCGAGGGTAAGCCCCTGTTCCCGGGCAAGGACCACGTGAACCAGTTCTCCATCATCACCGAGCTGCTGGGCA : 1213
              TCGAGGGTAAGCCCCTGTTCCCGGGCAAGGACCACGTGAACCAGTTCTCCATCATCACCGAGCTGCTGGGCA

                    *      1240         *      1260         *      1280         *
AIT_TRKH1a  : CGCCACCGGACGATGTCATCAACACTATTGCTAGCGAGAATGTGAGTTTCCCAGGGACATGATTGGGGCGCC : 1286
AIT_TRLF1e  : CGCCACCGGACGATGTCATCAACACTATTGCTAGCGAGAATGTGAGTTTCCCAGGGACATGATTGGGGCGCC : 1275
QM6a        : CGCCACCGGACGATGTCATCAACACTATTGCTAGCGAGAATGTGAGTTTCCCAGGGACATGATTGGGGCGCC : 1286
RUTC30      : CGCCACCGGACGATGTCATCAACACTATTGCTAGCGAGAATGTGAGTTTCCCAGGGACATGATTGGGGCGCC : 1286
AIT_TRKH1c  : CGCCACCGGACGATGTCATCAACACTATTGCTAGCGAGAATGTGAGTTTCCCAGGGACATGATTGGGGCGCC : 1295
CBS999.97   : CGCCACCGGACGATGTCATCAACACTATTGCTAGCGAGAATGTGAGTTTCCCAGGGACATGATTGGGGCGCC : 1285
AIT_TRMS44  : CGCCACCGGACGATGTCATCAACACTATTGCTAGCGAGAATGTGAGTTTCCCAGGGACATGATTGGGGCGCC : 1285
              CGCCACCGGACGATGTCATCAACACTATTGCTAGCGAGAATGTGAGTTTCCCAGGGACATGATTGGGGCGCC

              1300         *      1320         *      1340         *      1360
AIT_TRKH1a  : GGCGGGGAACTGATTTTGTCGGTAGACGTTGCGGTTCGTCAAGTCGCTGCCCAAGCGTGAGAGGCAGCCTCT : 1358
AIT_TRLF1e  : GGCGGGGAACTGATTTTGTCGGTAGACGTTGCGGTTCGTCAAGTCGCTGCCCAAGCGTGAGAGGCAGCCTCT : 1347
QM6a        : GGCGGGGAACTGATTTTGTCGGTAGACGTTGCGGTTCGTCAAGTCGCTGCCCAAGCGTGAGAGGCAGCCTCT : 1358
RUTC30      : GGCGGGGAACTGATTTTGTCGGTAGACGTTGCGGTTCGTCAAGTCGCTGCCCAAGCGTGAGAGGCAGCCTCT : 1358
AIT_TRKH1c  : GGCGGGGAACTGATTTTGTCGGTAGACGTTGCGGTTCGTCAAGTCGCTGCCCAAGCGTGAGAGGCAGCCTCT : 1367
CBS999.97   : GGCGGGGAACTGATTTTGTCGGTAGACGTTGCGGTTCGTCAAGTCGCTGCCCAAGCGTGAGAGGCAGCCTCT : 1357
AIT_TRMS44  : GGCGGGGAACTGATTTTGTCGGTAGACGTTGCGGTTCGTCAAGTCGCTGCCCAAGCGTGAGAGGCAGCCTCT : 1357
              GGCGGGGAACTGATTTTGTCGGTAGACGTTGCGGTTCGTCAAGTCGCTGCCCAAGCGTGAGAGGCAGCCTCT

                *      1380         *      1400         *      1420         *      1440
AIT_TRKH1a  : GCGAAACAAGTTCAAGAATGCAGACGATTCGGGTAAGAAACAACAAGGGTCATGACTCCTATACGCCGTGTA : 1430
AIT_TRLF1e  : GCGAAACAAGTTCAAGAATGCAGACGATTCGGGTAAGAAACAACAAGGGTCATGACTCCTATACGCCGTGTA : 1419
QM6a        : GCGAAACAAGTTCAAGAATGCAGACGATTCGGGTAAGAAACAACAAGGGTCATGACTCCTATACGCCGTGTA : 1430
RUTC30      : GCGAAACAAGTTCAAGAATGCAGACGATTCGGGTAAGAAACAACAAGGGTCATGACTCCTATACGCCGTGTA : 1430
AIT_TRKH1c  : GCGAAACAAGTTCAAGAATGCAGACGATTCGGGTAAGAAACAACAAGGGTCATGACTCCTATACGCCGTGTA : 1439
CBS999.97   : GCGAAACAAGTTCAAGAATGCAGACGATTCGGGTAAGGAACAACAAGGGTCATGACTCCTATACACCGTGTA : 1429
AIT_TRMS44  : GCGAAACAAGTTCAAGAATGCAGACGATTCGGGTAAGGAACAACAAGGGTCATGACTCCTATACACCGTGTA : 1429
              GCGAAACAAGTTCAAGAATGCAGACGATTCGGGTAAG AACAACAAGGGTCATGACTCCTATAC CCGTGTA

                        *      1460         *      1480         *      1500         *
AIT_TRKH1a  : GATGCTGACCCAAGGCAGCTGTCGATCTCTTGGAGCGCATGCTCGTCTTCGACCCCAAGAAGCGAATCACGG : 1502
AIT_TRLF1e  : GATGCTGACCCAAGGCAGCTGTCGATCTCTTGGAGCGCATGCTCGTCTTCGACCCCAAGAAGCGAATCACGG : 1491
QM6a        : GATGCTGACCCAAGGCAGCTGTCGATCTCTTGGAGCGCATGCTCGTCTTCGACCCCAAGAAGCGAATCACGG : 1502
RUTC30      : GATGCTGACCCAAGGCAGCTGTCGATCTCTTGGAGCGCATGCTCGTCTTCGACCCCAAGAAGCGAATCACGG : 1502
```

```
AIT_TRKH1c : GATGCTGACCCAAGGCAGCTGTCGATCTCTTGGAGCGCATGCTCGTCTTCGACCCCAAGAAGCGAATCACGG : 1511
CBS999.97  : GATGCTGACCCAAGGCAGCTGTCGATCTCTTGGAGCGCATGCTCGTCTTCGACCCCAAGAAGCGAATCACGG : 1501
AIT_TRMS44 : GATGCTGACCCAAGGCAGCTGTCGATCTCTTGGAGCGCATGCTCGTCTTCGACCCCAAGAAGCGAATCACGG : 1501
             GATGCTGACCCAAGGCAGCTGTCGATCTCTTGGAGCGCATGCTCGTCTTCGACCCCAAGAAGCGAATCACGG

                 1520         *      1540         *      1560         *      1580
AIT_TRKH1a : CCACCGAAGCCCTGGCCCACGAGTACCTCGCGCCATACCACGACCCTACTGACGAGCCGGTTGCCGAGGAAA : 1574
AIT_TRLF1e : CCACCGAAGCCCTGGCCCACGAGTACCTCGCGCCATACCACGACCCTACTGACGAGCCGGTTGCCGAGGAAA : 1563
QM6a       : CCACCGAAGCCCTGGCCCACGAGTACCTCGCGCCATACCACGACCCTACTGACGAGCCGGTTGCCGAGGAAA : 1574
RUTC30     : CCACCGAAGCCCTGGCCCACGAGTACCTCGCGCCATACCACGACCCTACTGACGAGCCGGTTGCCGAGGAAA : 1574
AIT_TRKH1c : CCACCGAAGCCCTGGCCCACGAGTACCTCGCGCCATACCACGACCCTACTGACGAGCCGGTTGCCGAGGAAA : 1583
CBS999.97  : CCACCGAAGCCCTGGCCCACGAGTACCTCGCGCCATACCACGACCCTACTGACGAGCCGGTTGCCGAGGAAA : 1573
AIT_TRMS44 : CCACCGAAGCCCTGGCCCACGAGTACCTCGCGCCATACCACGACCCTACTGACGAGCCGGTTGCCGAGGAAA : 1573
             CCACCGAAGCCCTGGCCCACGAGTACCTCGCGCCATACCACGACCCTACTGACGAGCCGGTTGCCGAGGAAA

                  *      1600         *      1620         *      1640         *
AIT_TRKH1a : AGTTTGACTGGAGCTTCAACGACGCCGACCTTCCTGTTGACACCTGGAAGATTATGATGTACGTCAACGTCC : 1646
AIT_TRLF1e : AGTTTGACTGGAGCTTCAACGACGCCGACCTTCCTGTTGACACCTGGAAGATTATGATGTACGTCAACGTCC : 1635
QM6a       : AGTTTGACTGGAGCTTCAACGACGCCGACCTTCCTGTTGACACCTGGAAGATTATGATGTACGTCAACGTCC : 1646
RUTC30     : AGTTTGACTGGAGCTTCAACGACGCCGACCTTCCTGTTGACACCTGGAAGATTATGATGTACGTCAACGTCC : 1646
AIT_TRKH1c : AGTTTGACTGGAGCTTCAACGACGCCGACCTTCCTGTTGACACCTGGAAGATTATGATGTACGTCAACGTCC : 1655
CBS999.97  : AGTTTGACTGGAGCTTCAACGACGCCGACCTTCCTGTTGATACCTGGAAGATTATGATGTACGTCAACGTCC : 1645
AIT_TRMS44 : AGTTTGACTGGAGCTTCAACGACGCCGACCTTCCTGTTGATACCTGGAAGATTATGATGTACGTCAACGTCC : 1645
             AGTTTGACTGGAGCTTCAACGACGCCGACCTTCCTGTTGA ACCTGGAAGATTATGATGTACGTCAACGTCC

             1660         *      1680         *      1700         *      1720
AIT_TRKH1a : ACCAATGCTCCATCGTGTCTCTTTGGTCTGACTTGAACACAGGTACTCGGAAATTCTGGACTACCACAACAT : 1718
AIT_TRLF1e : ACCAATGCTCCATCGTGTCTCTTTGGTCTGACTTGAACACAGGTACTCGGAAATTCTGGACTACCACAACAT : 1707
QM6a       : ACCAATGCTCCATCGTGTCTCTTTGGTCTGACTTGAACACAGGTACTCGGAAATTCTGGACTACCACAACAT : 1718
RUTC30     : ACCAATGCTCCATCGTGTCTCTTTGGTCTGACTTGAACACAGGTACTCGGAAATTCTGGACTACCACAACAT : 1718
AIT_TRKH1c : ACCAATGCTCCATCGTGTCTCTTTGGTCTGACTTGAACACAGGTACTCGGAAATTCTGGACTACCACAACAT : 1727
CBS999.97  : ACCAATGCTCCGTCGTGTCTCTTTGGTCTGACTTGAACACAGGTACTCGGAAATTCTGGACTACCACAACAT : 1717
AIT_TRMS44 : ACCAATGCTCCGTCGTGTCTCTTTGGTCTGACTTGAACACAGGTACTCGGAAATTCTGGACTACCACAACAT : 1717
             ACCAATGCTCC TCGTGTCTCTTTGGTCTGACTTGAACACAGGTACTCGGAAATTCTGGACTACCACAACAT

               *      1740         *      1760         *
AIT_TRKH1a : AGAGGGTGGTGTCCCAAACATGGACGAGCAGTTCCCGCCGCAGTAG : 1764
AIT_TRLF1e : AGAGGGTGGTGTCCCAAACATGGACGAGCAGTTCCCGCCGCAGTAG : 1753
QM6a       : AGAGGGTGGTGTCCCAAACATGGACGAGCAGTTCCCGCCGCAGTAG : 1764
RUTC30     : AGAGGGTGGTGTCCCAAACATGGACGAGCAGTTCCCGCCGCAGTAG : 1764
AIT_TRKH1c : AGAGGGTGGTGTCCCAAACATGGACGAGCAGTTCCCGCCGCAGTAG : 1773
CBS999.97  : AGAGGGTGGTGTCCCAAACATGGACGAGCAGTTCCCGCCGCAGTAG : 1763
AIT_TRMS44 : AGAGGGTGGTGTCCCAAACATGGACGAGCAGTTCCCGCCGCAGTAG : 1763
             AGAGGGTGGTGTCCCAAACATGGACGAGCAGTTCCCGCCGCAGTAG
```
